# A Wood Plasticine With Controlled Phase‐Change Behavior and Malleability for Energy‐Closed‐Loop and Conformally Adaptive Thermal Management

**DOI:** 10.1002/advs.75001

**Published:** 2026-03-25

**Authors:** Jiazuo Zhou, Yifan Liu, Lei Qiao, Yuehe Gu, Taikun Yao, Wenbo Zhang, Fangmiao Wang, Yudong Li, Xinyao Ji, Lingyi Kong, Haiyue Yang, Yao Xiao, Chengyu Wang

**Affiliations:** ^1^ Key Laboratory of Bio‐based Material Science and Technology of Ministry of Education Northeast Forestry University Harbin P. R. China; ^2^ College of Chemistry and Materials Engineering Wenzhou University Wenzhou P. R. China

**Keywords:** phase change material, wood, controlled heat release, geometric conformability, interfacial heat transfer

## Abstract

Solar heating enables energy conservation and carbon mitigation. However, this heating method struggles with the spontaneous heat loss, geometrically diverse heat‐demanding objects, and inefficient interfacial heat transfer. Herein, we fabricate a wood plasticine with controlled phase‐change and malleable characteristics, achieved through the synergistic effects of molecular‐scale and nano/micro‐scale hydrogen bonding networks. The supercooled wood plasticine enables stable room‐temperature solar heat storage for 48 h, owing to a raised nucleation energy barrier resulting from molecular‐scale hydrogen bonding networks between erythritol and glycerol. Through a force‐induced supercooling‐crystallization phase change, the wood plasticine can controllably release 108 J g^−1^ of stored solar heat. The wood‐derived cellulose dispersed in erythritol and glycerol enables the formation of nano/micro‐scale hydrogen bonding networks. The synergy between glycerol's fluidity and the spatial confinement of cellulose fibers enables the reversible disruption and reconstruction of multiscale hydrogen bonding networks under external pressure. Driven by this dynamic property, the wood plasticine achieves conformal contact with geometrically diverse heat‐demanding objects, reducing contact thermal resistance by 28.7%. An energy‐closed‐loop system based on a harvest‐storage‐release mechanism utilizing wood plasticine is conceptually demonstrated to enable versatile applications, such as thermoelectric conversion and battery/personal thermal management, providing a new paradigm for on‐demand and low‐loss solar heating.

## Graphical abstract

A photothermal wood plasticine exhibiting controlled phase‐change behavior and malleable property is developed. The developed wood plasticine can provide all‐day, on‐demand, and low‐loss solar heating for geometrically diverse objects, including thermoelectric generator, human body, and battery, through its controlled crystallization‐melting‐supercooling phase change and malleability‐induced interfacial conformability. This research introduces a new paradigm for the solar heat‐driven clean energy revolution.

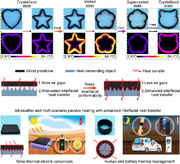

## 1 Introduction

Utilizing clean solar energy for heating through photothermal conversion provides a viable strategy to alleviate dependence on fossil fuel combustion [1, 2]. Photothermal composite phase change materials (PCMs) are considered efficient solar heat carriers that address the intermittent availability of solar energy caused by temporal, climatic, and geographical factors [3, 4]. In the absence of sunlight, they can release the solar heat stored as latent heat during the photothermal process through a melting‐crystallization phase change [5, 6]. The application domains of photothermal composite PCMs are transitioning from traditional environmental space thermal management (e.g., building energy conservation) to advanced microclimate‐based interventions (e.g., personal thermal management, temperature control of photovoltaic thermal systems, and battery thermal management) [7, 8, 9, 10, 11]. Nevertheless, this transition also presents substantial challenges. Specifically, the latent heat release resulting from the melting‐crystallization phase change occurs uncontrollably and spontaneously due to the low nucleation energy barrier, leading to a mismatch between heat supply and demand [12]. Additionally, due to the limited geometric conformability of traditional photothermal composite PCMs, the heat generated through photothermal conversion or melting‐crystallization phase change can only be transferred to shape‐specific heat‐demanding objects. Moreover, the heat transfer is significantly hindered by the tiny interfacial air gaps between photothermal composite PCMs and heat‐demanding objects [13]. These challenges limit the effectiveness and universality of solar heat utilization for complex and variable heat‐demanding applications.

To address the above‐mentioned challenges, an effective method is to endow composite PCMs with controlled phase‐change behavior and malleable property. Controlled phase‐change behavior is primarily achieved through two strategies: molecular design and compositional design of PCMs. Molecular design typically leverages classical aliphatic PCMs, such as n‐alkanes, fatty alcohols, and fatty acids [14, 15, 16]. Specifically, azobenzene dopants are covalently bound to these PCMs’ molecules. This enables the photoisomerization of composite PCMs under UV light, thereby primarily suppressing spontaneous melting‐crystallization phase change through van der Waals forces. Compositional design frequently utilizes erythritol and mannitol as PCMs. Unlike molecular design, this strategy relies on hydrogen bonding interactions between these PCMs and a non‐phase‐change component to suppress spontaneous phase change. Reported hydrogen‐bonding partners include carrageenan, polyvinyl alcohol, polyacrylamide, and sodium hydroxide [12, 17, 18, 19]. The two design strategies essentially suppress spontaneous crystallization by increasing the nucleation energy barrier. The increased energy barrier can be reduced by external stimuli, such as visible light irradiation or heating, enabling controlled crystallization of composite PCMs.

Under the premise of controlled phase change, to endow composite PCMs with excellent malleability, the constructed hydrogen bonding networks require further compositional optimization to achieve reversible disruption‐reconstruction under external stimuli. Cellulose, a biomacromolecule derived from wood and other plants, consists of molecular chains composed of repeating D‐glucose units linked by covalent bonds. Owing to the inherent hydroxyl groups on D‐glucose units, cellulose has been widely utilized as a raw material to establish hydrogen bonding networks for the fabrication of various malleable materials [20, 21]. For example, when cellulosic material is exposed to water, water molecules can weaken the hydrogen bonds between cellulose molecular chains, resulting in notable hydroplastic behavior (reversible disruption and reconstruction of hydrogen bonding) under external force [22]. Few studies achieve both controlled phase change and malleability.

**FIGURE 1:**
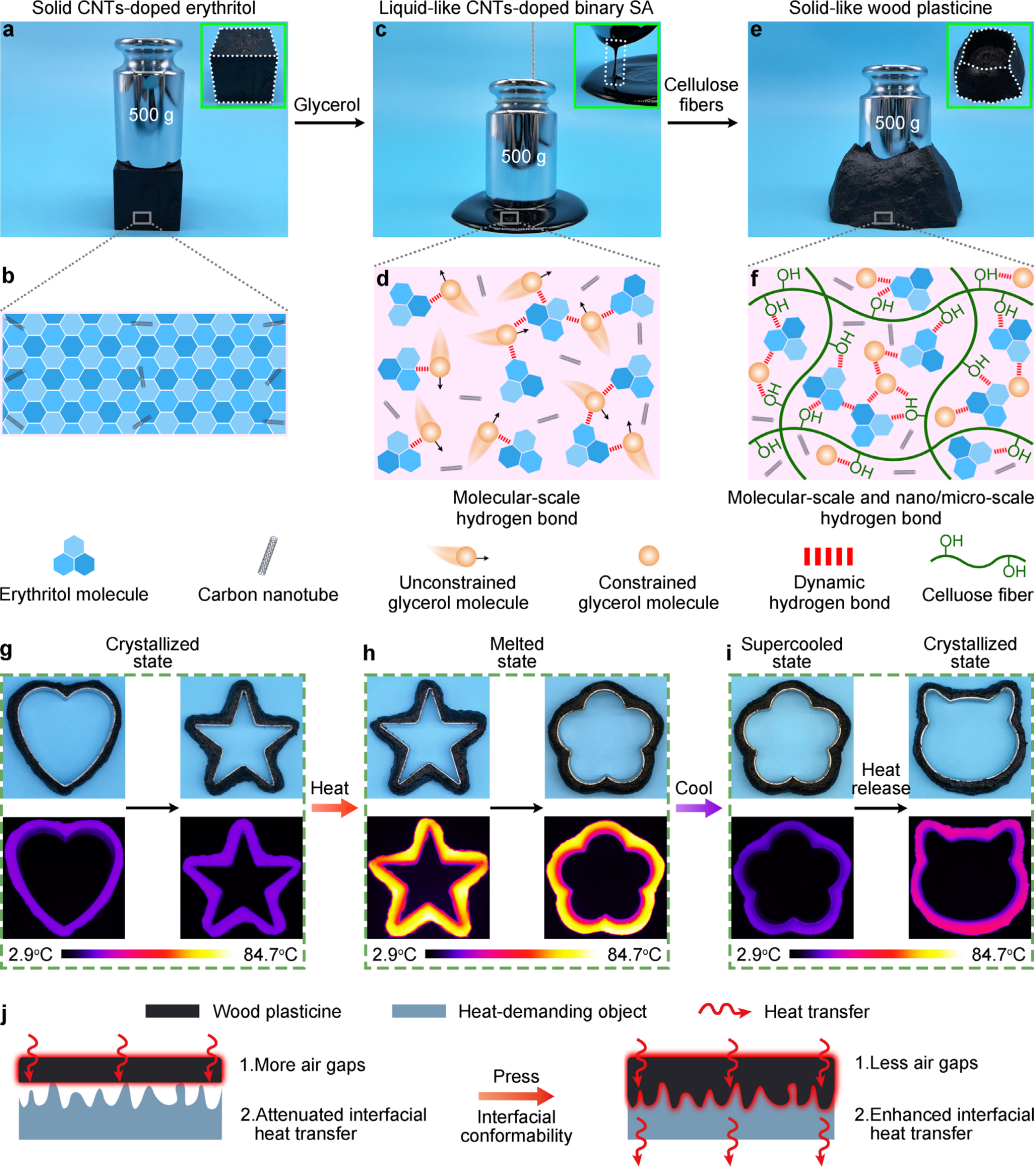
The compositional design strategy, multiscale hydrogen bonding networks, controlled phase change, malleability, and heat transfer characteristics of wood plasticine. Photographs of (a) CNTs‐doped erythritol, (c) CNTs‐doped binary SA (the mixture of erythritol and glycerol is referred to as “binary SA”), and (e) wood plasticine under a 500 g load. The corresponding insets illustrate the shape stability (solid behavior) of CNTs‐doped erythritol, the thread‐forming ability (liquid‐like behavior) of CNTs‐doped binary SA, and the shape retention (solid‐like behavior) of wood plasticine after the removal of the load. Schematic illustrations of (b) erythritol molecules doped with CNTs, (d) molecular‐scale hydrogen bonding networks between erythritol molecules andunconstrained glycerol molecules, and (f) multiscale hydrogen bonding networks among erythritol molecules, constrained glycerol molecules, and cellulose fibers. Geometric conformability of wood plasticine to (g) heart‐shaped and five‐pointed star‐shaped forms (crystallized state), (h) five‐pointed star‐shaped and flower‐shaped forms (melted state), and (i) flower‐shaped and cat‐head‐shaped forms (during controlled supercooling‐crystallization phase change). (j) Comparison of non‐conformal and conformal contact between wood plasticine and rough surface during heat transfer, highlighting the enhanced interfacial heat transfer induced by conformability.

Herein, by sequentially incorporating glycerol and cellulose fibers into erythritol doped with photothermal carbon nanotubes (CNTs) to establish molecular‐scale and nano/micro‐scale hydrogen bonding networks, respectively, we fabricate a wood plasticine with controlled phase‐change behavior and malleability (Figure 1a–f). The molecular‐scale interaction increases the nucleation energy barrier of wood plasticine to prevent spontaneous crystallization, maintaining it in a supercooled state. The synergistic effects of molecular‐scale and nano/micro‐scale hydrogen bonding networks enable the constructed multiscale hydrogen bonding networks to undergo reversible disruption and reconstruction (dynamic behavior) under external force (Figure 1f). This characteristic confers superior malleability on the wood plasticine in its crystallized, melted, and supercooled states (Figure 1g–i). When subjected to an external force, supercooled wood plasticine undergoes a geometric configuration transformation accompanied by a controlled supercooling‐crystallization phase change. This phase change process, enabled by a reduced nucleation energy barrier, results in a temperature increase of approximately 16.4°C in the material (Figure 1i). Moreover, the malleable wood plasticine can establish excellent conformal contact with the rough surface of the heat‐demanding object under external pressure, which eliminates air gaps and enhances interfacial heat transfer performance (Figure 1j). Consequently, the contact thermal resistance is reduced by 28.7%, resulting in a value of 88.1 ± 6.1 mm^2^ W^−1^ K^−1^. The developed wood plasticine achieves energy‐closed‐loop heating through efficient harvesting, long‐term storage, and on‐demand utilization of solar heat, advancing the heat‐demanding applications like thermoelectric conversion and battery/personal thermal management.

## 2 Results and Discussion

### 2.1 Synergistic Effects of Molecular‐Scale and Nano/Micro‐Scale Hydrogen Bonding Networks Within Wood Plasticine

**FIGURE 2:**
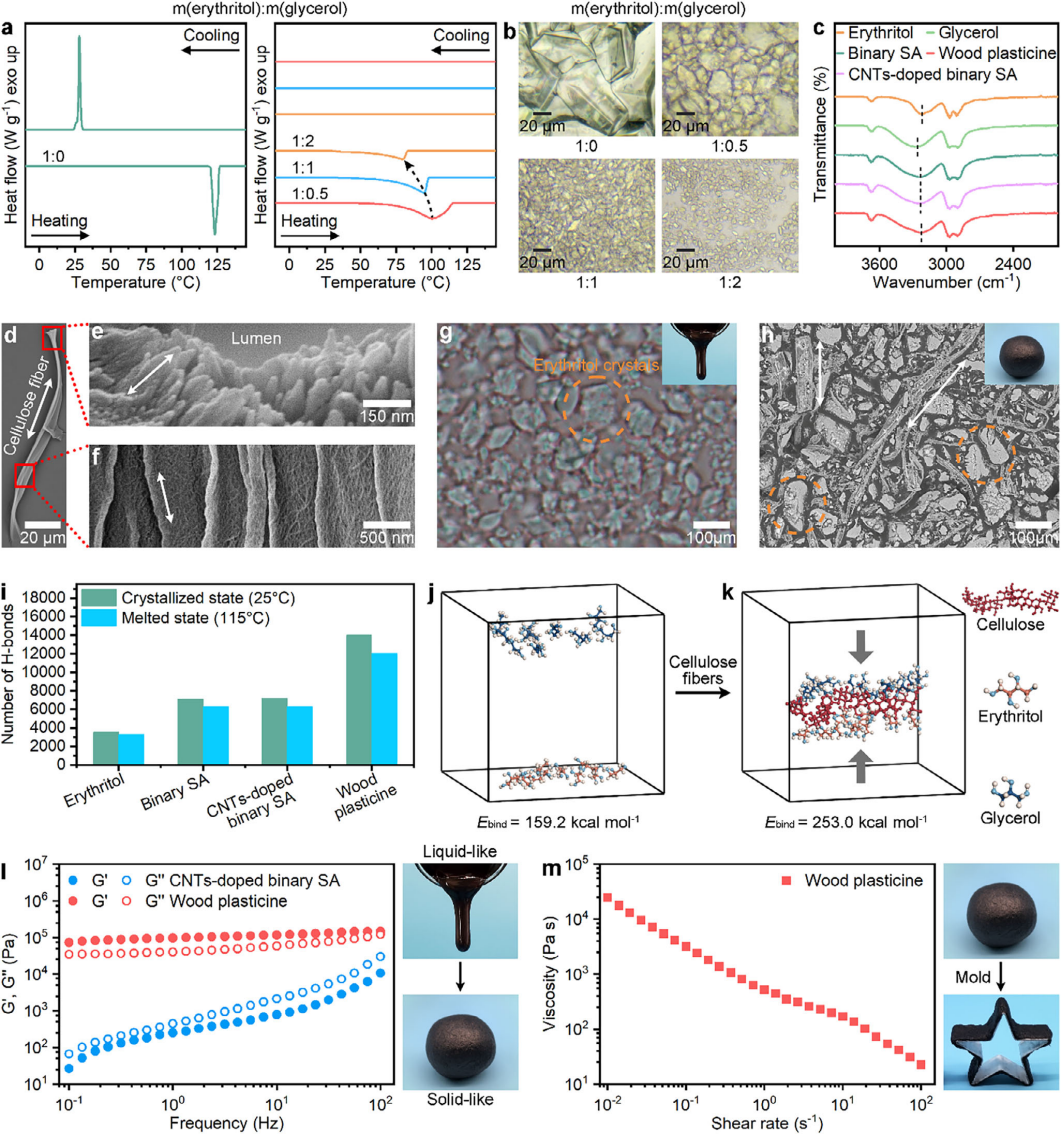
Synergistic effects of molecular‐scale and nano/micro‐scale hydrogen bonding networks within wood plasticine. (a) DSC curves and (b) OM images of erythritol and binary SA during the heating and cooling process at different mass ratios of erythritol to glycerol (1:0, 1:0.5, 1:1, and 1:2). (c) FT‐IR spectra of erythritol, glycerol, binary SA, CNTs‐doped binary SA, and wood plasticine. (d) SEM image depicting the micro‐scale structure of a single cellulose fiber separated from wood aerogel. (e, f) High‐magnification SEM images revealing the nano‐scale structure of the single cellulose fiber. (g) OM image of binary CNTs‐doped binary SA (inset: photograph of CNTs‐doped binary SA). (h) SEM image of wood plasticine (inset: photograph of wood plasticine). (i) Number of hydrogen bonds (H‐bonds) of erythritol, binary SA, CNTs‐doped binary SA, and wood plasticine simulated by MD. Binding energy simulation models of (j) CNTs‐doped binary SA and (k) wood plasticine after MD simulation. The CNTs in the simulated model have been hidden. The whole model is presented in Figure S18a,b. (l) Frequency sweep measurements of CNTs‐doped binary SA and wood plasticine in the crystallized state (25°C); Photographs showing the transformation from liquid‐like behavior to solid‐like behavior. (m) Shear rate sweep of wood plasticine in the crystallized state (25°C); Photographs illustrating the programmability of wood plasticine.

The phase change material (PCM) selected in this study is erythritol, which is rich in hydroxyl groups [23]. To achieve the controlled phase‐change behavior of erythritol by enhancing its supercooling capability, a binary sugar alcohol (SA) composite is fabricated through the physical blending of erythritol with glycerol. A differential scanning calorimeter (DSC) is used to characterize the melting point, melting enthalpy, and supercooling characteristics of erythritol and binary SA. The melting temperature of erythritol is 120.3°C ± 1.5°C, with a corresponding melting enthalpy of 322.7 ± 1.7 J g^−1^ (Figure 2a; Table S1). As the mass ratio of erythritol to glycerol decreases from 1:0.5 to 1:2, both the melting temperature and melting enthalpy of binary SA show a decreasing trend. Specifically, the melting temperature decreases from 82.5 ± 1.3°C to 65.5 ± 0.8°C, while the melting enthalpy decreases from 170.1 ± 1.1 to 68.1 ± 1.3 J g^−1^ (Figure 2a; Table S1). The erythritol releases all stored latent heat through the spontaneous melting‐crystallization phase change in the temperature range of 23°C–30°C (Figure 2a). Unlike the behavior of erythritol, the binary SA maintains at supercooled state in the same temperature range (from 23°C to 30°C) to achieve long‐term latent heat storage (Figure 2a). Optical microscopy (OM) is used to investigate the crystallization behavior of erythritol and binary SA. Figure S1a,b demonstrates OM images of erythritol upon cooling from its melted state at 140°C. A great number of erythritol crystals are observed to form within a cooling period of 1 min at room temperature. On the contrary, no crystals are observed in the OM images of binary SA upon cooling to room temperature from the melted state at 140°C, which is consistent with the supercooled state characterized by DSC. As the mass ratio of erythritol to glycerol decreases from 1:0.5 to 1:2, the time for the binary SA to remain supercooled gradually increases from 6 to 96 h (Figure S1c–h). Moreover, the crystal size of erythritol in binary gradually decreases from 74.7 ± 9.8 to 8.1 ± 0.9 µm with the increasing glycerol content, which can be regarded as an indirect indication of the influence of glycerol on the crystallization behavior of erythritol (Figure 2b; Figure S1i and Table S1). Additionally, the supercooling‐crystallization phase change can be triggered on demand via mechanical stirring (Movie S1 and Figure S2). Meanwhile, the long‐stored latent heat is controllably released. Considering the trade‐off between energy density and duration of latent heat storage, the binary SA used in the following studies is prepared by physically blending erythritol with glycerol at a mass ratio of 1:1 (Figure S3 and Table S1).

Molecular‐scale hydrogen bonding networks are formed between erythritol and glycerol molecules, increasing the nucleation energy barrier of binary SA [17]. This increased energy barrier delays the spontaneous crystallization of erythritol, thereby facilitating long‐term latent heat storage [12, 18]. The Fourier transform infrared spectroscopy (FT‐IR) and molecular dynamics (MD) simulations are employed to systematically characterize the formation of molecular‐scale hydrogen bonding networks (Figure 2c; Figure S4a–d). The broad characteristic peaks at 3000–3600 cm^−1^ derive from the stretching vibration of hydroxy groups in the molecular structure of erythritol, glycerol, and binary SA. The stretching vibration peak of the hydroxyl groups of binary SA moves from 3280 to 3237 cm^−1^ compared with that of glycerol, which indicates the formation of hydrogen bonding networks between erythritol molecules and glycerol molecules [17]. The MD simulations reveal that the number of hydrogen bonds in binary SA, irrespective of whether they are in the crystallized or melted state, is twice as high as that observed in erythritol (Figure S4e; Figure 2i). These findings are consistent with the results obtained from FT‐IR analysis.

The all‐cellulose wood aerogel, composed of nano‐ and micro‐scale cellulose fibers, is directly prepared from natural balsa wood using a top‐down approach (Figure S5a–h and S6a–c) [24, 25, 26]. All‐cellulose wood aerogel is referred to as wood aerogel in the following context. A single cellulose fiber with excellent flexibility and bendability is easily separated from the wood aerogel through mild mechanical exfoliation, owing to the complete removal of binder (lignin‐hemicellulose matrix) (Figure S7a,b) [27]. The scanning electron microscope (SEM) image clearly illustrates the nano/micro‐scale structures of a single cellulose fiber (Figure 2d–f; Figure S8). Whether at the nanoscale or microscale, cellulose fibers demonstrate highly aligned characteristics.

Based on the existing controlled phase change property induced by molecular‐scale hydrogen bonding networks, nano/micro‐scale hydrogen bonding networks are further constructed to enable the malleability of CNTs‐doped binary SA by incorporating nano/micro‐scale cellulose fibers derived from wood aerogel [28, 29, 30]. Specifically, the interactions among cellulose fibers within the aerogel are first effectively weakened by infiltrating the liquid‐like CNTs‐doped binary SA into the hierarchically porous structure of the wood aerogel. Then, a malleable wood plasticine is prepared by kneading the infiltrated wood aerogel, during which cellulose fibers are exfoliated from the wood aerogel and are uniformly dispersed within the CNTs‐doped binary SA (Figures S9–S12). In addition to the previously mentioned balsa wood, cellulose fibers used to construct the nano/micro‐scale hydrogen bonding networks can also be derived from various wood species, such as basswood, beech, poplar, spruce, and pine, as well as from processing residues (e.g., wood chips, flours, and shavings) generated from such woods. This demonstrates the potential for resource diversification of cellulose fibers (Figures S13 and S14). CNTs are utilized as photothermal additives to endow wood plasticine with photothermal capability. The microstructure of CNTs‐doped binary SA is observed using an OM image. The erythritol crystals with uniform particle size are uniformly dispersed in the amorphous glycerol (Figure 2g). After the kneading process, the aligned cellulose fibers are separated from the bulk wood aerogel and uniformly distributed in the erythritol crystals and the amorphous glycerol (Figure 2h; Figure S15a,b). The FT‐IR spectrum is employed to characterize the nano/micro‐scale hydrogen bonding networks between binary SA and cellulose fiber (Figure 2c). Compared to binary SA, the hydroxyl stretching vibration peak of CNTs‐doped binary SA exhibits negligible shifting, suggesting that the addition of CNTs has an insignificant impact on hydrogen bond formation. The hydroxyl stretching vibration peak of wood plasticine shifts from 3238 to 3227 cm^−1^ in comparison to that of CNTs‐doped binary SA, due to the formation of nano/micro‐scale hydrogen bonding networks induced by the uniform dispersion of cellulose fibers. MD simulation is used to further demonstrate the effects of CNTs and cellulose fibers on hydrogen bond formation at the nano/micro scale. The MD snapshots reveal that the introduction of CNTs has no impact on the homogeneous distribution of erythritol molecules and glycerol molecules, regardless of whether the binary SA is in its crystallized or melted state (Figure S4a–d and S16a,b). Additionally, there is no significant difference in the number of hydrogen bonds between binary SA and CNTs‐doped binary SA for both their crystallized and melted states (Figure 2i). Unlike the effect of CNTs, cellulose fibers separated from wood aerogel result in twice as many hydrogen bonds in wood plasticine as in both binary SA and CNTs‐doped binary SA, regardless of whether these materials are in the crystallized or melted state (Figure S17a,b; Figure 2i). Moreover, upon the incorporation of cellulose, the binding energy of the entire material system increases significantly from 159.2 to 253.0 kcal mol^−1^ (Figure 2j,k; Figure S18a,b). This shows that the addition of cellulose fibers can strengthen the internal interactions within the entire material system, which is consistent with the increase in the number of hydrogen bonds [31].

The malleability‐induced geometric conformability, as reflected by programmable and self‐healing properties of wood plasticine, is evaluated through rheological characterizations. The linear viscoelastic regions of CNTs‐doped binary SA and wood plasticine are extremely narrow in both their crystallized and melted states (Figure S19a,b). Therefore, the 0.1% strain is utilized for conducting frequency sweep tests and shear rate sweep [32]. The storage modulus (G’) is always lower than the loss modulus (G’) for CNTs‐doped binary SA in both its crystallized and melted states, demonstrating its liquid‐like behavior (Figure 2l; Figure S20) [33]. The liquid‐like properties of CNTs‐doped binary SA are attributed to the presence of liquid glycerol within it. In contrast, the wood plasticine exhibits an elastic solid‐like behavior, as evidenced by the G’ being greater than the G’ (Figure 2l; Figure S20) [34]. The rheological behavior of CNTs‐doped binary SA and wood plasticine exhibits distinct differences, which is attributed to the increased number of hydrogen bonds in the wood plasticine (Figure 2i) [35]. The wood plasticine exhibits typical shear‐thinning behavior in both its crystallized and melted states (Figure 2m; Figure S21). Under external force, the wood plasticine exhibits an extremely low viscosity due to the disruption of dynamic multiscale hydrogen bonding networks within it, enabling its conformal adaptation to the rough surfaces of geometrically diverse objects, such as heart‐shaped, five‐pointed star‐shaped, flower‐shaped, and cat‐head‐shaped forms (Figure 1g–i and Figure 2m) [36]. After the shaping process, there is a substantial increase in the viscosity of wood plasticine owing to the reconstruction of previously disrupted dynamic multiscale hydrogen bonding networks, which ensures its shape retention [37]. Therefore, this shear‐thinning property endows the wood plasticine with programmable property. The wood plasticine demonstrates a reversible fusion‐division process in both its crystallized and melted states when subjected to external force, evidencing its self‐healing property (Figure S22a,b). Additionally, the wood plasticine demonstrates an exceptionally low compression modulus, with values of 10.9 ± 2.0 MPa in the crystallized state and 2.0 ± 0.5 MPa in the melted state, highlighting its inherent softness (Figure S23).

### 2.2 Photothermal Conversion, Interfacial Heat Transfer, and Long‐Term Latent Heat Storage Performances of Wood Plasticine

**FIGURE 3:**
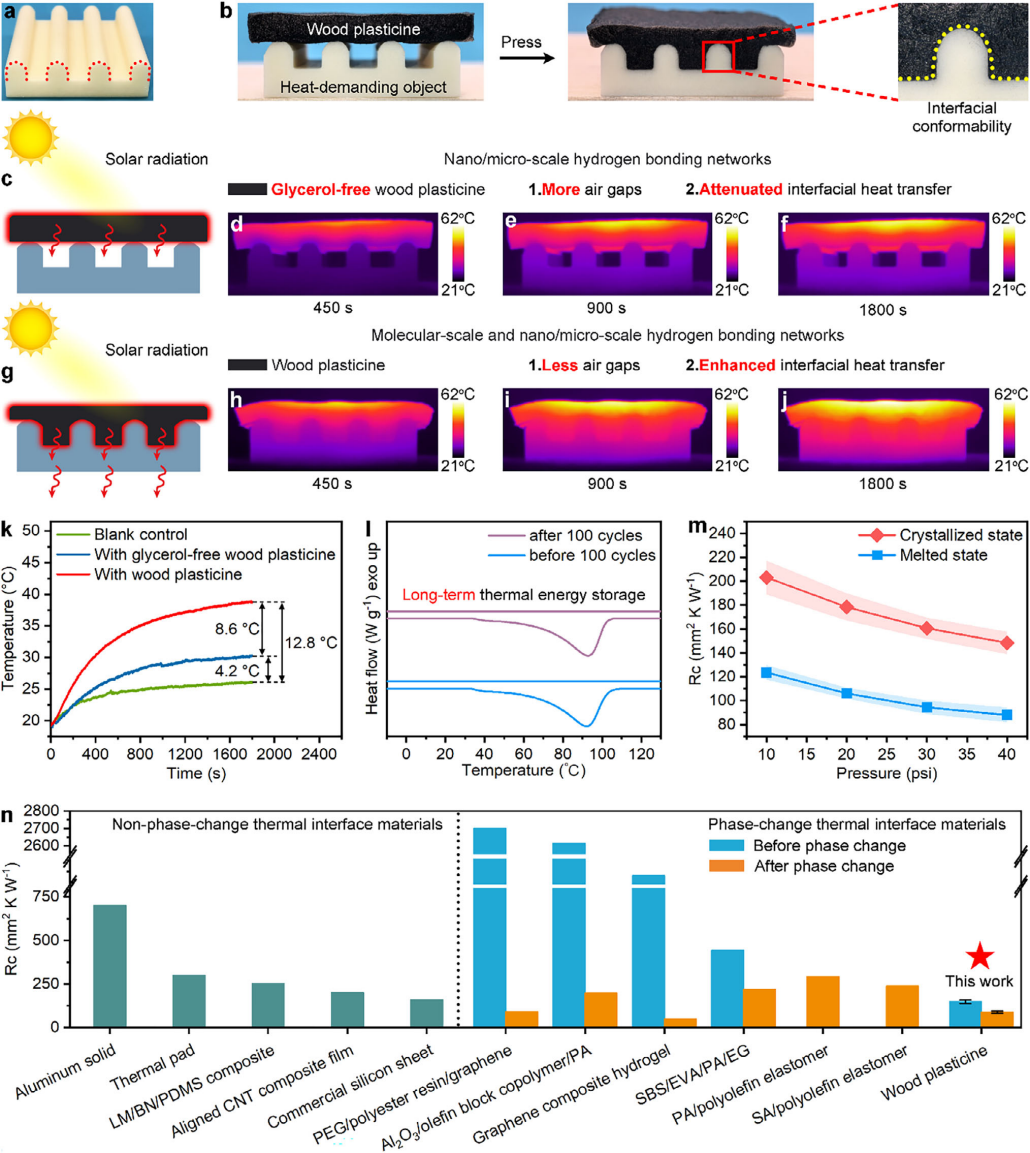
Photothermal conversion, interfacial heat transfer, and long‐term latent heat storage performances of wood plasticine. (a) Photograph of a heat‐demanding object with an arch‐like rough surface. (b) Photographs illustrating conformal contact between wood plasticine and a heat‐demanding object under external pressure. (c) Schematic diagram of a heat‐demanding object coated with glycerol‐free wood plasticine under solar radiation. Infrared thermal images of heat‐demanding object coated with glycerol‐free wood plasticine under varied durations of simulated solar radiation: (d) 450 s, (e) 900 s, and (f) 1800 s. (g) Schematic diagram of heat‐demanding object coated with wood plasticine under solar radiation. Infrared thermal images of heat‐demanding object coated with wood plasticine under varied durations of simulated solar radiation: (h) 450 s, (i) 900 s, and (j) 1800 s. (k) Time‐temperature curves of heat‐demanding object uncoated with glycerol‐free wood plasticine or wood plasticine, coated with glycerol‐free wood plasticine, and coated with wood plasticine under simulated solar radiation. (l) DSC curves of wood plasticine before and after 100 thermal charging–discharging cycles. (m) Contact thermal resistance (*R*_c_) of crystallized and melted wood plasticine at different pressures. (n) Comparative analysis of contact thermal resistance (*R*_c_) between wood plasticine and previously reported thermal interface materials (TIMs), including both non‐phase‐change and phase‐change TIMs. *Note*: liquid metal (LM), boron nitride (BN), polydimethylsiloxane (PDMS), carbon nanotube (CNT), polyethylene glycol (PEG), paraffin (PA), styrene‐butadiene‐styrene polymer (SBS), ethylene vinyl acetate (EVA), expanded graphite (EG), and stearic acid (SA).

The photothermal conversion ability of wood plasticine gradually improves as the mass fraction of CNTs increases from 0% to 0.2% (Figure S24a–d). Under one solar intensity irradiation, when the mass fraction of CNTs is 0.1% or 0.2%, the temperature of wood plasticine increases by 18°C in both cases, reaching a maximum of 43.8°C. In this article, the mass fraction of CNTs in the wood plasticine is 0.1%, unless stated otherwise. The malleable wood plasticine, possessing hydrogen bonding networks at both the molecular and nano/micro scales, exhibits remarkable conformal adaptability to the arch‐like rough surface of a heat‐demanding object under external pressure (Figure 3a,b). In contrast, the CNTs‐doped binary SA, which contains only molecular‐scale hydrogen bonding networks, exhibits excellent flowability on the arch‐like rough surface due to its liquid‐like behavior (Figure 2l; Figures S20 and S25). Moreover, the wood plasticine that incorporates only nano/micro‐scale hydrogen bonding networks, referred to as glycerol‐free wood plasticine, undergoes near‐brittle fracture on the arch‐like rough surface under external pressure (Figure S26a,b) [30]. Consequently, it is the synergistic effects of molecular‐scale and nano/micro‐scale hydrogen bonding networks that endow the wood plasticine with excellent malleability.

The influence of molecular‐scale and nano/micro‐scale hydrogen bonding networks on the malleability of wood plasticine directly leads to the differences in its interfacial heat transfer performance. The CNTs‐doped binary SA containing only molecular‐scale hydrogen bonding networks should have served as a logical control. However, owing to its inherent flowability, the CNTs‐doped binary SA is unsuitable for testing and further practical applications. As illustrated in the infrared thermal images, both glycerol‐free wood plasticine and wood plasticine successfully convert simulated solar energy into solar heat, enabling targeted heating of the heat‐demanding object (Figure S27a–d; Figure 3c–j). As the duration of solar illumination increases, the average temperature of the heat‐demanding object gradually rises and ultimately stabilizes at a constant value of 38.8°C after 1800 s (Figure 3k). The application of glycerol‐free wood plasticine and wood plasticine can elevate the average temperature of bare heat‐demanding object by 4.2°C and 12.8°C, respectively. The solar heat generated by the glycerol‐free wood plasticine cannot be effectively transferred to the heat‐demanding object owing to significant interfacial heat loss caused by the presence of air gaps (Figure 3c–f) [38]. Conversely, in the presence of molecular‐scale hydrogen bonding networks, the interfacial heat transfer between the wood plasticine and the heat‐demanding object is markedly enhanced under external pressure (Figure 3g–j). Specifically, the cross‐sectional temperature gradient between the wood plasticine and the heat‐demanding object becomes more uniform, indicating that a negligible temperature difference between them [39]. The enhanced interfacial heat transfer is attributed to the improved interfacial conformability, arising from the synergistic effects of molecular‐scale and nano/micro‐scale hydrogen bonding networks. Therefore, the average temperature of the heat‐demanding object treated with wood plasticine is 8.6°C higher than that of the object treated with glycerol‐free wood plasticine (Figure 3k). Moreover, the compression‐induced enhanced interfacial heat transfer method also enables targeted heating of the object with a square‐like rough surface, demonstrating the universality of this method for geometrically diverse heat‐demanding objects with complex and variable rough surfaces (Figure S28a–o).

During the targeted heating of the object, the wood plasticine concurrently undergoes the crystallization‐melting phase change, storing solar heat as latent heat. In the absence of sunlight, the thermally charged wood plasticine undergoes supercooling and maintains long‐term latent heat storage for up to 48 h (Figure S29a–d). This suggests that the wood plasticine inherits the long‐term heat storage characteristic of its internal binary SA component. Notably, the wood plasticine can stably store latent heat for a duration of up to 12 months at temperatures as low as −40°C (Figure S30a–d). The long‐term latent heat storage property of wood plasticine is further investigated using DSC (Figure 3l). The wood plasticine exhibits a melting temperature of 75.9°C ± 1.9°C and a melting enthalpy of 114.1 ± 1.1 J g^−1^ upon heating. No crystallization peak is observed during the subsequent cooling process. These results suggest that no spontaneous latent heat release occurs when the thermally charged wood plasticine is removed from the heat source. Furthermore, after undergoing 100 thermal charging‐discharging cycles, the wood plasticine maintains its excellent long‐term latent heat storage characteristic. Notably, glycerol‐free wood plasticine lacks the capability for long‐term latent heat storage, indicating that the molecular‐scale hydrogen bonding networks are a critical factor in enabling this capability (Figure S31).

After the phase change, the thermal conductivity of wood plasticine decreases from 0.421 ± 0.002 to 0.351 ± 0.003 W m^−1^ K^−1^ (a reduction of only 16.7%), ensuring efficient thermal charging during photothermal conversion (Figure S32 and Table S2). The contact thermal resistance is a crucial parameter for evaluating the interfacial heat transfer between wood plasticine and heat‐demanding objects (Figure S33). The essence of compression‐induced enhanced interfacial heat transfer lies in the minimization of contact thermal resistance, which is achieved by significantly reducing the air gaps between wood plasticine and heating‐demand objects under applied pressure. As the pressure increases, the contact thermal resistance between wood plasticine and heating‐demand objects progressively decreases, ultimately stabilizing at 148.3 ± 9.4 mm^2^ K W^−1^ in the crystallized state and 88.1 ± 6.1 mm^2^ K W^−1^ in the melted state (Figure 3m; Table S3). This phenomenon is attributed to the improved conformability between the two surfaces during the compression process [40]. Additionally, after the phase change, the erythritol crystals in the wood plasticine progressively transform from a crystallized solid state to a melted liquid state. This transformation leads to a decrease in the compressive modulus (Figure S23), improving the softness and interfacial conformability of wood plasticine [41]. Therefore, the contact thermal resistance decreases as the temperature increases, analogous to the effect of pressure, and ultimately approaches a relatively stable value of 88.1 ± 6.2 mm^2^ K W^−1^ (Figure S34 and Table S4). Notably, the contact thermal resistance of wood plasticine, both before and after its phase change, is significantly lower compared to previously reported thermal interface materials (TIMs), encompassing both non‐phase‐change TIMs (such as aluminum solid, thermal pad, LM/BN/PDMS composite, aligned CNT composite film, and commercial silicon sheet) and phase‐change TIMs (including PEG/polyester resin/graphene, Al_2_O_3_/olefin block copolymer/PA, graphene composite hydrogel, SBS/EVA/PA/EG, PA/polyolefin elastomer, and SA/polyolefin elastomer) (Figure 3n; Tables S5 and S6). In conjunction with its malleability‐induced geometric conformability, photothermal wood plasticine possesses the potential to provide sustainable solar heat with minimal heat loss in complex and variable heat‐demanding scenarios.

### 2.3 Controlled Latent Heat Release Performance of Wood Plasticine

**FIGURE 4:**
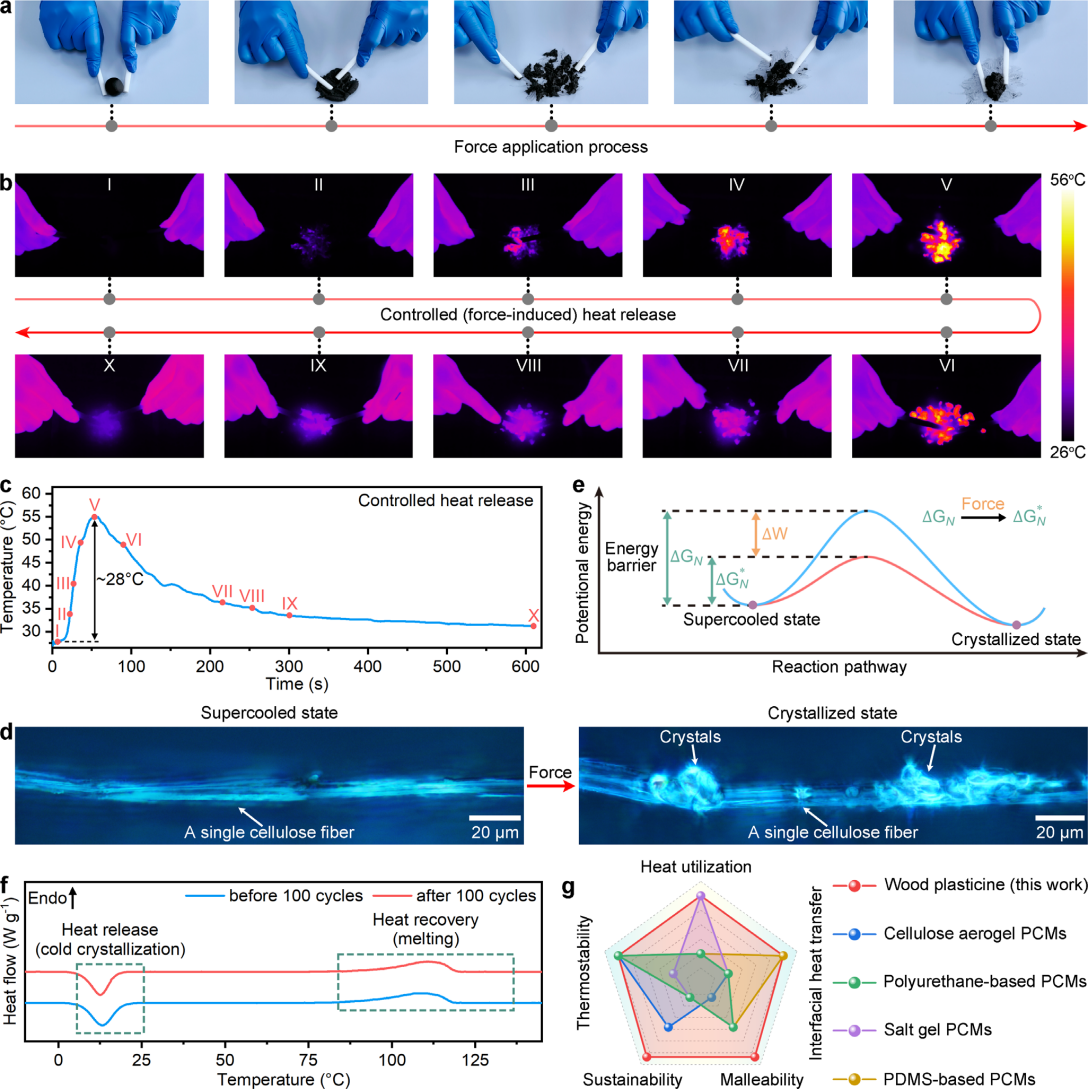
Controlled latent heat release performance of wood plasticine. (a) Photographs depicting the application of continuous force to wood plasticine. (b) Infrared thermal images illustrating the temperature changes of wood plasticine under external force. (c) Time‐temperature curve of wood plasticine derived from infrared thermal images. (d) OM images illustrating the force‐induced crystallization process of wood plasticine. (e) Schematic diagram illustrating the effective reduction of nucleation energy barrier by an external force. (f) DSC curves illustrating latent heat release and subsequent latent heat recovery of wood plasticine before and after 100 thermal charging‐discharging cycles. (g) Radar plot illustrating the comparison of cellulose aerogel PCMs, polyurethane‐based PCMs, salt gel PCMs, PDMS‐based PCMs, and the wood plasticine proposed in this study.

After the targeted heating of heat‐demanding object is completed, the latent heat stored in the supercooled wood plasticine can be released on demand through the application of external force (Figure 4a). The temperature variations of wood plasticine are recorded by an infrared thermal camera under external force (Movie S2; Figure 4b). The temperature of wood plasticine increases from 28°C to 56°C (a maximum rise of 28°C) within 54 s, after which it gradually decreases due to latent heat dissipation (Figure 4c). The latent heat release process is accompanied by the phase change of wood plasticine from its supercooled state to its crystallized state. As shown by the OM images, erythritol crystals are observed to grow on the surface of a single cellulose fiber separated from the wood plasticine after the complete release of latent heat induced by external force (Figure 4d). Additionally, the gradual decrease in transmittance of binary SA is employed to visually demonstrate the macroscopic growth of erythritol crystals during the supercooling‐crystallization phase change (Movie S1 and Figure S2). This phase change from the supercooled state to the crystallized state is attributed to a reduction of the nucleation energy barrier between these two states under external force (Figure 4e). The nucleation energy barrier is reflected through the Gibbs free energy of nucleation using Equation 1:

(1) $$\Delta G_{N}=-\frac{4\pi }{3}r^{3}\Delta \mu_{v}+4\pi r^{2}\gamma_{s}$$

where Δ*G*_N_ is the Gibbs free energy of nucleation, r is the radius of embryos, Δ*µ*_v_ is the volume term in the Gibbs free energy, γ_s_ is the solid–liquid interfacial energy of erythritol [12]. The external force provides external energy to offset the increased nucleation energy barrier induced by the molecular‐scale hydrogen bonding networks, as demonstrated by Equation 2:

(2) $$\Delta G_{N}^{*}=-\frac{4\pi }{3}r^{3}\Delta \mu_{v}+4\pi r^{2}\gamma_{s}+\Delta W$$

where Δ*G* ^*^_N_ is the Gibbs free energy of nucleation and ΔW is the extra energy [42]. Consequently, the energy barrier between supercooled and crystallized states of wood plasticine is reduced under external force compared to the absence of external force, enabling controlled latent heat release. To accurately measure the latent heat of crystallization under external force, the wood plasticine is placed in the DSC furnace and reheated to promote its cold crystallization. Cold crystallization refers to the process wherein PCMs transition from their supercooled state to their crystallized state under thermal stimulation [18]. Consequently, similar to the effect of applying an external force, heating can also reduce the increased nucleation energy barrier resulting from molecular‐scale hydrogen bonding networks between the supercooled state and the crystallized state. The crystallization enthalpy of wood plasticine is 108.3 ± 1.2 J g^−1^, which is comparable to the melting enthalpy (Figure 4f). Furthermore, the wood plasticine that has released latent heat can recovery latent heat by absorbing heat from the surrounding environment. After 100 cycles of thermal charging and discharging, the wood plasticine still shows reliable performance for storing latent heat and releasing it on demand. Overall, when the wood plasticine is compared with other common composite PCMs, such as cellulose aerogel PCMs, polyurethane‐based PCMs, salt gel PCMs, and polydimethylsiloxane (PDMS)‐based PCMs, it exhibits superior performance in terms of heat utilization, interfacial heat transfer, malleability, sustainability, and thermostability. (Figure 4g; Table S7).

### 2.4 Thermoelectric Power Generation and Synchronous Long‐Term Latent Heat Storage via Photothermal Conversion

**FIGURE 5:**
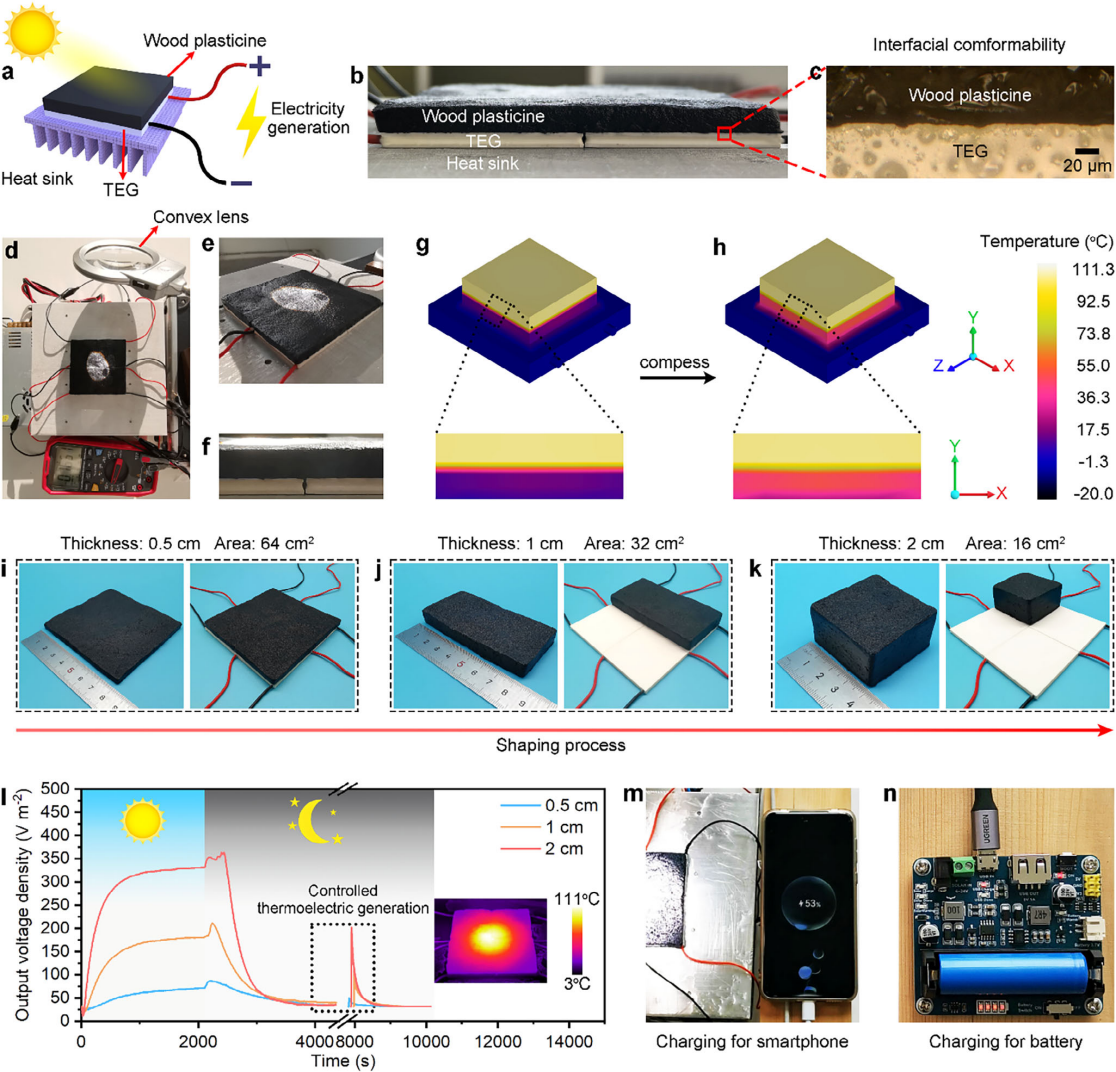
Thermoelectric power generation and synchronous long‐term latent heat storage via photothermal conversion. (a) Schematic diagram and (b) Photograph of solar‐thermal‐electric conversion apparatus, with the TEG sandwiched between the wood plasticine (hot side) and the heat sink (cold side). (c) OM image showing the conformal contact at the interface between the wood plasticine and the hot side of TEG. (d) Top view, (e) perspective view, and (f) side view of solar‐thermal‐electric conversion apparatus under focused sunlight; The measured focused solar intensity is 600 mW cm^−2^. Comparison of interfacial heat transfer performance between wood plasticine and TEG (g) before compression and (h) after compression. (i–k) Control over the height of the wood plasticine and the contact area between the wood plasticine and the TEG through a shaping process. (l) Customizable and controlled electrical output of solar‐thermal‐electric conversion apparatus via a shaping process and the application of an external force; Infrared thermal imaging of wood plasticine with a thickness of 0.5 cm and a contact area of 64 cm^2^ under focused sunlight (inset). Photographs depicting a large‐scale TEG utilized for charging (m) a smartphone and (n) a 14500 lithium‐ion battery.

As a proof‐of‐concept for mitigating the global electricity shortage, wood plasticine, leveraging its photothermal property and compression‐induced enhanced interfacial heat transfer capacities, is utilized as the hot side of a heat‐demanding thermoelectric generator (TEG) for green electricity generation (Figure 5a) [43]. In the home‐made solar‐thermal‐electric conversion apparatus, the TEG is sandwiched between the wood plasticine (hot side) and the heat sink (cold side) (Figure 5b; Figure S35). The OM image reveals the conformal contact at the interface between the wood plasticine and the hot side of TEG under external pressure (Figure 5c) [13]. Fewer air gaps are observed at the interface, thereby ensuring excellent interfacial heat transfer between the wood plasticine and the TEG. The convex lens is strategically positioned at an optimal height above the solar‐thermal‐electric conversion apparatus to focus ambient sunlight, maximizing the temperature of the TEG's hot side (Figure 5d–f). Finite element simulation software (ANSYS Icepak) is employed to visualize the interfacial heat transfer process between the wood plasticine and the TEG, as well as to validate the enhanced interfacial heat transfer induced by pressure (Figure 5g,h; Table S8). After the interfacial air gaps are eliminated by the compression of wood plasticine, the temperatures at the upper and lower ends of the interface increase by 15.1°C and 32.5°C, respectively (Figure S36a,b). Furthermore, the TEG in contact with pressed wood plasticine exhibits a temperature difference between its hot and cold sides that is 21°C higher than that of the TEG in contact with unpressed wood plasticine. These simulated results indicate that the compression process significantly enhances interfacial heat transfer between the wood plasticine and the TEG during the solar‐thermal‐electric conversion. For TEGs of any size, such as a large‐scale TEG assembled by connecting four smaller TEGs in series, the electrical output can be customized to meet diverse electrical requirements (Figure S37). This customizable electrical output is achieved by precisely adjusting the thickness of the wood plasticine and the contact area between the wood plasticine and the TEG through a meticulous shaping process (Figure 5i–k) [44]. The solar heat generated via photothermal conversion is conducted from the upper surface to the lower surface of wood plasticine along its thickness. As the thickness of wood plasticine reduces, the heat loss decreases during the heat transfer process [45, 46, 47]. For the same wood plasticine sample, as its thickness decreases, the contact area between the wood plasticine and the TEG increases accordingly. Additionally, the contact area between the wood plasticine and the TEG is equivalent to the heated area on the hot side of TEG. As the thickness of the wood plasticine is reduced 2 to 0.5 cm through a shaping process, the temperature on its lower surface increased from 49.2°C to 64.3°C (Figure S38a,b). Consequently, the output voltage density of solar‐thermal‐electric conversion apparatus increases from 72 to 331 V m^−2^ (Figure 5l). As illustrated in the infrared thermal imaging, the surface temperature of wood plasticine reaches 111°C, while the temperature of heat sink is 3°C (Figure 5l, inset). This significant temperature difference consequently results in a substantial temperature difference between the hot and the cold sides of TEG, enabling green electricity generation under focused ambient sunlight. By utilizing a boost converter, a large‐scale TEG comprising four smaller TEGs connected in series can generate sufficient electricity to charge a smartphone, a 14 500 lithium‐ion battery, and an LED (Figure 5m,n; Figure S39a–c) [48]. Meanwhile, the latent heat is effectively stored within wood plasticine through its crystallization‐melting phase change (Figure 3l). In the absence of sunlight, the latent heat remains stably stored in the wood plasticine without spontaneous heat loss, owing to its melting‐supercooling phase change. The stored latent heat can be controllably released to enable thermoelectric power generation without sunlight [49]. By appropriately adjusting the thickness of the wood plasticine from 2 to 0.5 cm, an output voltage density ranging from 50 to 202 V m^−2^ can be achieved (Figure 5l).

### 2.5 Controlled Latent Heat Release for Battery and Personal Thermal Management in the Absence of Sunlight

**FIGURE 6:**
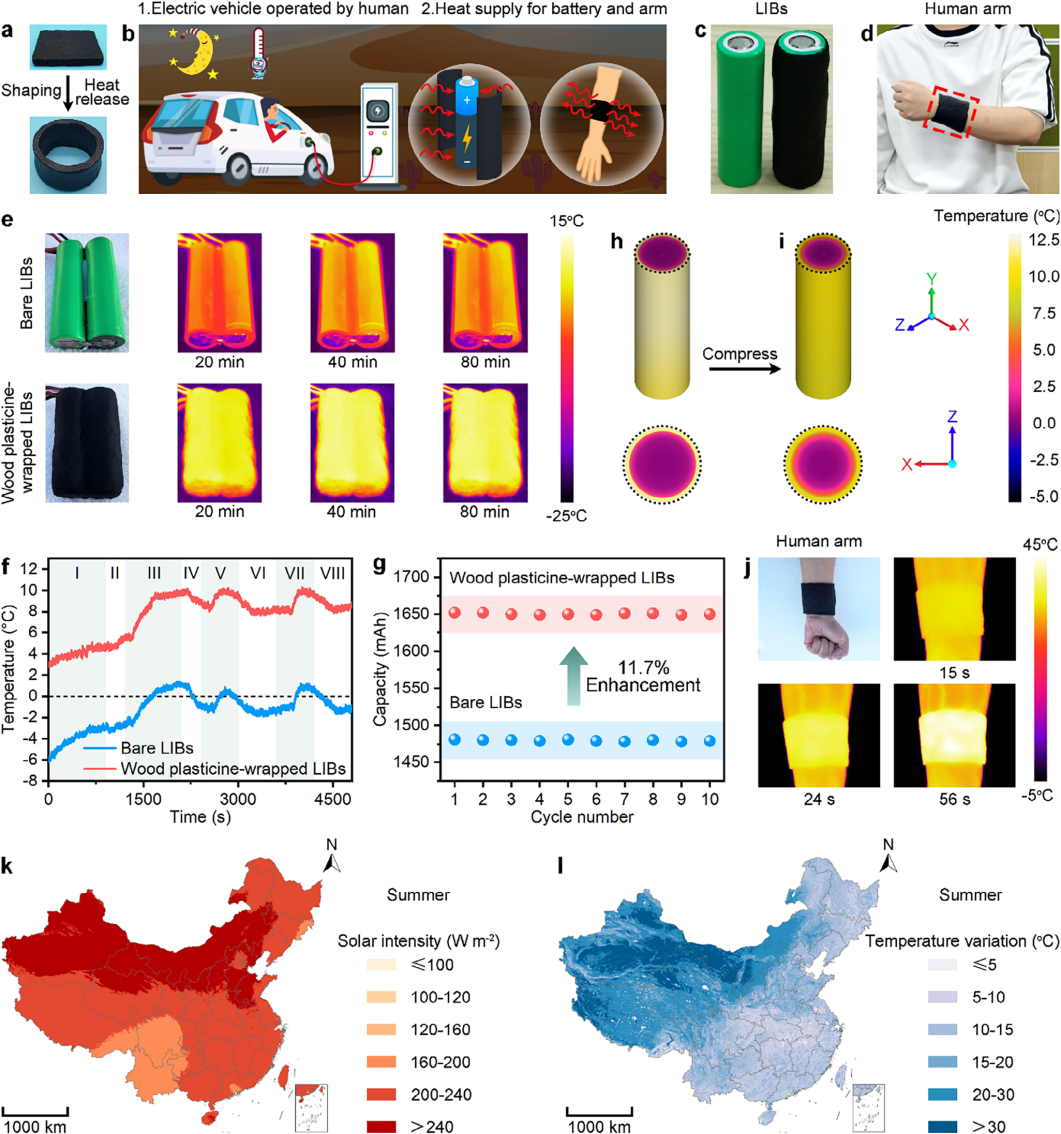
Controlled latent heat release for battery and personal thermal management in the absence of sunlight. (a) Transformation of wood plasticine from cuboid shape to tube shape companied with heating characteristic through shaping process. (b) Schematic representation of the integration among human‐operated electric vehicle, battery thermal management, and personal thermal management. Photographs showing (c) a lithium‐ion battery and (d) a human arm wrapped with exothermic wood plasticine. (e) Photographs of bare and wood plasticine‐wrapped LIBs, along with their corresponding infrared thermal images during charge–discharge cycling. Comparison of (f) time‐temperature curves and (g) effective charge capacity for bare and wood plasticine‐wrapped LIBs over 10 charge–discharge cycles. Comparison of interfacial heat transfer performance between wood plasticine and battery (h) before compression and (i) after compression. (j) Photograph of a wood plasticine‐wrapped human arm, together with its corresponding infrared thermal images. (k) Solar intensity distribution and (l) diurnal temperature variation distribution across different regions of China during summer.

Electric vehicles (EVs), powered by lithium‐ion batteries (LIBs), have achieved widespread adoption and rapid development due to their significant contributions to decarbonization [50]. However, when EVs are operated in low‐temperature environments (below 0°C), their LIBs usually undergo capacity degradation, reduced charging efficiency, and an elevated risk of internal short circuits (Figure S40) [51]. Furthermore, the low‐temperature environments may also pose significant health risks to drivers of EVs. As a proof‐of‐concept, both LIBs and the human arm are selected as heat‐demanding objects in low‐temperature environments without sunlight radiation. The cuboid‐shaped wood plasticine, used for thermoelectric generation under sunlight radiation, is reshaped into a tubular form to enable conformal wrapping around both the LIBs and the human arm (Figures 5d–f and 6a–d). During this shaping process, the energy barrier between the supercooled and the crystallized states of wood plasticine is reduced, allowing for the controlled release of its latent heat stored during solar‐thermal‐electric conversion (Figure 4e). The released latent heat is subsequently transferred to both the LIBs and the human arm, offering a promising solution to ensure that EVs can successfully complete travel tasks under low‐temperature environments, such as during cold nights.

The temperatures of bare and wood plasticine‐wrapped 18 650 LIBs are measured to evaluate the heating performance of the wood plasticine during charge–discharge cycling in low‐temperature environments (Figure 6e,f) [52, 53]. As illustrated in the infrared thermal images, the wood plasticine provides spatially uniform heating for the LIBs operating at low‐temperature environments, promoting an increase in the overall temperature of the LIBs (Figure 6e). This spatially uniform heating is attributed to the simultaneous formation of numerous erythritol crystal nuclei throughout the interior of the wood plasticine during the shaping process (Figure S2; Figure 4b) [54, 55]. The temperature of LIBs wrapped in the exothermic wood plasticine is consistently higher than that of bare LIBs (Figure 6f; Table S9). Owing to the heating characteristic of wood plasticine, when the temperature of bare LIBs reaches as low as around −6°C, the temperature of wrapped LIBs is maintained at about 2°C. At a charging rate of 4 C, the maximum temperature of wrapped LIBs reaches 10.3°C, representing an increase of 35.3°C compared to the low‐temperature room temperature. As a result, the effective charge capacity of LIBs wrapped with the exothermic wood plasticine is enhanced by approximately 11.7% over 10 charge–discharge cycles, in comparison to bare LIBs (Figure 6g) [53, 56, 57]. During the shaping process, which provides the necessary heat for the LIBs, applying appropriate pressure to the wood plasticine can enhance the interfacial heat transfer between the wood plasticine and the target LIBs, reducing heat loss to a minimum. This improvement is attributed to the conformal wrapping induced by applied pressure. As demonstrated by the simulation results obtained from ANSYS Icepak, the temperature of LIBs wrapped with pressed wood plasticine is 3.5°C higher than that of LIBs wrapped with unpressed wood plasticine (Figure 6h,i; Figure S41a,b and Table S10). Regarding the provision of heat to the human body, wood plasticine can uniformly increase surface body temperature, particularly in the arm region, by up to 18°C within 56 s during its controlled latent heat release process (Figure 6j; Figure S42).

The above results indicate that wood plasticine enables the cyclic harvesting, storage, and utilization of solar heat (Figure S43a–f). In its cuboid form, during the heat harvesting process, the waste heat can be utilized for heat‐demanding applications such as thermoelectric power generation (Figure S43a,b). The harvested latent heat can be stored stably within the wood plasticine over the long term (Figure S43c). After further molding the cuboid wood plasticine into a tubular structure, the material enables controlled release of long‐term stored solar heat and exhibits excellent interfacial heat transfer capability, thereby meeting complex and variable heat‐demanding requirements, such as battery and personal thermal management (Figure S43d,e). After releasing the latent heat, the wood plasticine can be reused for the next cycle of solar harvesting, storage, and utilization (Figure S43f). The cyclic harvesting, storage, and utilization of solar heat hold significant promise for long‐distance thermal transportation and multi‐scenario thermal applications, accompanied by minimal interfacial heat loss [58, 59]. In summer, in regions with abundant solar energy resources where the solar radiation intensity exceeds 240 W m^−2^ (Figure 6k; Figure S44a–c), this wood plasticine can efficiently harvest solar heat via photothermal conversion, store this heat in the form of latent heat, and ultimately achieve long‐term latent heat storage. Notably, some regions experiencing a year‐round diurnal temperature variation exceeding 30°C face severe challenges, including water scarcity, human health risks, and increased energy consumption (Figure 6l; Figure S45a–c) [51, 60]. In view of this, the latent heat stored in wood plasticine for a long time can be transported to these regions to address the aforementioned challenges. Upon arrival, the wood plasticine can release the long‐stored latent heat under external force, enabling heat supply for various heat‐demanding scenarios.

## 3 Conclusion

In summary, by implementing a multiscale hydrogen bonding networks construction strategy, this study endows the fabricated photothermal wood plasticine with controlled phase‐change and malleable characteristics. The molecular‐scale hydrogen bonding networks enable this wood plasticine to store the harvested solar heat for up to 48 h at room temperature while eliminating spontaneous heat loss. Under external force, the wood plasticine releases long‐stored solar heat through the supercooling‐crystallization phase change, increasing its temperature by 28°C at room temperature. The synergistic effects of molecular‐scale and nano/micro‐scale hydrogen bonding networks endow wood plasticine with exceptional malleability, enabling its geometric adaptability to complex and variable heat‐demanding scenarios (such as thermoelectric conversion, battery thermal management, and personal thermal management) through the efficient harvesting, long‐term storage, and controlled utilization of solar heat. Additionally, under external pressure, this malleability‐induced interfacial conformal conformability can the reduce contact thermal resistance by 28.7%. Under the guidance of multiscale hydrogen bonding network design strategy, the range of available raw materials can be expanded to include other hydroxyl‐rich phase change materials (such as D‐mannitol, polyethylene glycol, and fatty alcohols) as well as plant fibers (e.g., cotton, bamboo, and straw). This broadens the diversity and application potential of raw material sources. The study introduces an innovative research paradigm for the effective manipulation of solar heat through the construction of multiscale hydrogen bonding networks, thereby promoting widespread access to affordable and clean energy.

## Author Contributions

J.Z.Z., H.Y.Y., Y.X., and C.Y.W. conceived and designed this experiment. J.Z.Z., Y.F.L., and L.Q. performed the experiment and wrote the manuscript. J.Z.Z., Y.H.G., T.K.Y., W.B.Z., F.M.W., Y.D.L., and X.Y.J., and L.Y.K contributed to analyzing the data. All authors discussed the results and commented on the paper.

## Conflicts of Interest

The authors declare no conflicts of interest.

## Supporting information




**Supporting File 1**: advs75001‐sup‐0001‐SuppMat.docx

**Supporting File 2**: advs75001‐sup‐0002‐MovieS1.mp4

**Supporting File 3**: advs75001‐sup‐0003‐MovieS2.mp4

## Data Availability

The data that support the findings of this study are available from the corresponding author upon reasonable request.
