# Supplementary material for: A Wood Plasticine With Controlled Phase‐Change Behavior and Malleability for Energy‐Closed‐Loop and Conformally Adaptive Thermal Management
Source: Adv Sci (Weinh). 2026 Mar 25;13(32):e75001. doi: 10.1002/advs.75001 (PMC13252630; doi:10.1002/advs.75001)
Supplement: Supplementary file 1 — Supporting File 1: advs75001‐sup‐0001‐SuppMat.docx [file ADVS-13-e75001-s003.docx]

Supporting Information for

**A wood plasticine with controlled phase-change behavior and malleability for energy-closed-loop and conformally adaptive thermal management**

*Jiazuo Zhou,* *Yifan Liu, Lei Qiao, Yuehe Gu, Taikun Yao, Wenbo Zhang, Fangmiao Wang, Yudong Li,* *Xinyao Ji,* *Lingyi Kong,* *Haiyue Yang*, Yao Xiao *, and* *Chengyu Wang **

J. Zhou, Y. Liu, L. Qiao, Y. Gu, T. Yao, W. Zhang, F. Wang, Prof. Y. Li, X. Ji, Prof. H. Yang, Prof. C. Wang

Key Laboratory of Bio-based Material Science and Technology of Ministry of Education, Northeast Forestry University, Harbin 150040, P. R. China

E-mail: [haiyueyang@nefu.edu.cn](mailto:haiyueyang@nefu.edu.cn); [wangcy@nefu.edu.cn](mailto:wangcy@nefu.edu.cn)

L. Kong, Prof. Y. Xiao

College of Chemistry and Materials Engineering, Wenzhou University, Wenzhou 325035, P. R. China

E-mail: [xiaoyao@wzu.edu.cn](mailto:xiaoyao@wzu.edu.cn)

**Keywords:** phase change material, wood, controlled heat release, geometric conformability, interfacial heat transfer

**Experimental Section:**

*Materials and Chemicals*: Natural wood samples, including balsa wood, basswood, beech, poplar, spruce, and pine, are purchased from a local supplier in Heilongjiang Province, China. Sodium chlorite (NaClO_2_, 80%), sodium hydroxide (NaOH, 95%), erythritol (99%), and glycerol (99%, AR) are purchased from Shanghai Macklin Biochemical Co., Ltd. Glacial acetic acid (CH_3_COOH, AR) is obtained from Tianli Chemical Reagent Co., Ltd. Hydroxylated carbon nanotubes are purchased from Shenzhen Suiheng Technology Co., Ltd.

*Preparation of all-celluose wood aerogel*: The wood samples are subjected to a series of sequential chemical treatment processes. Initially, the wood samples are immersed in a 2% NaClO_2_ solution at pH 4.6 (adjusted using CH_3_COOH) at 85 ℃ for 24 hours to achieve delignification. In the meanwhile, the NaClO_2_ solution is refreshed every 12 hours to maintain its efficacy. Following this step, the delignified wood samples are thoroughly rinsed with deionized water multiple times, each rinse lasting one hour, until the pH of the rinse solution reaches neutrality. Subsequently, these wood samples are treated with an 8% NaOH solution at 85 ℃ for 24 hours, with the solution being replaced every 12 hours to ensure efficient reaction conditions. Afterward, the treated samples undergo multiple one-hour rinses with deionized water until the rinse solution achieves neutrality. Finally, the processed samples are dried in a freeze dryer for 48 hours to produce all-celluose wood aerogels.

*Preparation of* *binary sugar alcohol*: The erythritol is initially heated to a temperature of 170 ℃ until it is completely melted. Subsequently, binary sugar alcohol (SA) is prepared by mixing melted erythritol with glycerol at mass ratios of 1:0.5, 1:1, and 1:2. This mixing process is conducted under magnetic stirring at a speed of 500 rpm for 5 minutes, while maintaining the temperature at 170 ℃, to ensure molecular-level uniformity. Based on the different mass ratios of erythritol to glycerol, the obtained binary SA is categorized as binary SA (1:0.5), binary SA (1:1), and binary SA (1:2). Upon cooling the melted binary SA to room temperature, the supercooled binary SA is formed. The supercooled binary SA can be converted into crystallized binary SA through either mechanical stirring or long-term placement. In this study, the description of the phase state of binary SA refers specifically to the phase state of the erythritol within it.

*Preparation of* *carbon nanotubes-doped binary* *sugar alcohol*: Carbon nanotubes-doped binary sugar alcohol is prepared by mixing melted binary SA (1:1) with carbon nanotubes (CNTs) at mass fractions of 0.0125%, 0.025%, 0.05%, 0.1%, and 0.2%. This mixing process is carried out at a constant temperature of 170 ℃ using a magnetic stirrer operating at 500 rpm for 5 minutes to ensure homogeneous mixing. After cooling the mixture to room temperature, the supercooled CNTs-doped binary SA is obtained. This supercooled material can be transformed into crystallized CNTs-doped binary SA either by mechanical stirring or through prolonged placement. In this study, the description of the phase state of CNTs-doped binary SA refers specifically to the phase state of the erythritol within it.

*Preparation of wood* *plasticine*: The prepared all-cellulose wood aerogel is immersed in the melted CNTs-doped binary SA at a temperature of 170 ℃, with a CNTs mass fraction of 0.1%. To ensure complete infiltration, five vacuum/de-vacuum cycles are conducted. After cooling the infiltrated all-cellulose wood aerogel to room temperature, it is transformed into a wood plasticine through thorough mechanical kneading. Notably, the preparation process of wood plasticine involves mechanical kneading under external force, resulting in the obtained material that is in the crystallized state. For the preparation of a wood plasticine without photothermal ability, the CNTs-doped binary SA in the above-mentioned preparation process can be directly replaced with the binary SA (1:1), while the preparation method remains unchanged. In this study, the description of the phase state of wood plasticine refers specifically to the phase state of the erythritol within it.

**Characterization**

The temperatures of the samples are measured using an infrared thermal camera (FLIR, E76, USA). The latent heat storage and release characristics of the samples are evaluated using a differential scanning calorimeter (DSC, PerkinElmer, 8000, USA) within the temperature range of -10 ℃ to 145 ℃, with a heating and cooling rate of 5 ℃ min^-1^ under a nitrogen flow of 20 mL min^-1^. The crystal growth in the samples is monitored using a metallurgical microscope (Optec, MIT500, China). Additionally, this metallurgical microscope is employed to characterize the microstructure of a single cellulose fiber and the interfacial conformability between wood plasticine and heat-demanding object. Fourier transform infrared spectroscopy (FT-IR, PerkinElmer, Waltham, MA, USA) is utilized to analyze the hydrogen bonding interactions and the chemical compositions of the samples within the wavenumber range of 1000-4000 cm^-1^. The morphologies of the samples are examined and analyzed using a scanning electron microscope (SEM, Thermoscientific, Apreo S, USA). The rheological measurements of crystallized and melted samples are conducted using a rotational rheometer (Anton Paar MCR 302, Austria) equipped with a 25 mm parallel plate at respective temperatures of 25 ℃ and 115 ℃, corresponding to their physical states.The strain amplitude sweep tests are conducted over a range of 0.1% to 100% strain at a fixed frequency of 1 Hz. The frequency sweep tests are carried out over a range of 0.1 Hz to 100 Hz at a constant strain of 0.1%. The shear rate sweep tests are performed within the range of 0.01 to 100 s^−1^. The alternate step strain test is executed within the strain range of 0.1% to 50% at a fixed frequency of 1 Hz for three cycles. For all these tests, the samples are placed between the two plates of the rheometer with a gap of 1 mm. The 18650 lithium-ion battery pack is employed to assess the thermal management performance of wood plasticine in low-temperature environments. The charge-discharge cycling of lithium-ion battery pack, as well as its capacity testing, are carried out using smart chargers (HOTA, D6 Pro, China). The X-ray diffractometer (XRD, Shimadzu, 6100, Japan) is used to analyze the crystal structure of the samples wthin the 2θ range of 10° to 80°, using a scanning speed of 5° min^-1^. The relative contents of cellulose, hemicellulose, and lignin are determined in accordance with the GB/T 2677.8-1994, GB/T 2677.10-1995, and GB/T 744-1989 standards. The X-ray microtomography analysis is performed using the SkyScan 1272 (Bruker, Germany) to obtain detailed 3D microstructural information. Compressive and three-point bending tests are performed using a universal testing machine (UTM2503, Suns, China), with a constant loading and unloading rate of 5 mm min^-1^. The absorbance of wood plasticine are measured using a UV-VIS-NIR spectrometer (Lambda 1050+, PerkinElmer, USA) equipped with a 150 mm integrating sphere over a wavelength range of 200-2500 nm. Thermal conductivity is measured using a thermal constant analyzer (Hot Disk, TPS 2500 s, Sweden).

**Molecular dynamics simulation**

Molecular dynamics simulation are performed using Materials Studio 2019 software. The COMPASS II force field is utilized. For the blend system, erythritol, glycerol, carbon nanotubes, and cellulose are blended into a box measuring 69.6 × 69.6 × 69.6 nm³, followed by the equilibrium process being carried out. To eliminate the stress generated during the modeling process, geometric optimization is performed on the model. To further optimize the structure, the mixtures are initially equilibrated at 298 K in the canonical (NVT) ensemble for 300 ps using the Berendsen thermostat. Subsequently, a multistep equilibration process lasting 500 ps is performed in the NPT ensemble to ensure thorough equilibration of the system. After the equilibration process, a 20 ps molecular dynamics simulation is conducted to facilitate further analysis.

**Binding energy simulation**

To simulate the interfacial configuration among erythritol, glycerol, carbon nanotube, and cellulose, molecular dynamic simulations are performed using Materials Studio 2019 software. The structures of erythritol, glycerol, carbon nanotubes, and cellulose are constructed and geometrically optimized using the Forcite module. The entire system is modeled with minimum initial energy under COMPASS II force field at 298 K.

**Solar intensity distribution and diurnal temperature variation distribution**

Solar intensity distribution and diurnal temperature variation distribution across different regions of China are obtained using ArcGIS Pro 3.3.2.^[1, 2]^ China’s administrative divisions: this map is based on the standard map provided by the Map Technology Review Center of the Ministry of Natural Resources (Review Number: GS(2019)1831). The boundary of the base map remains unaltered.


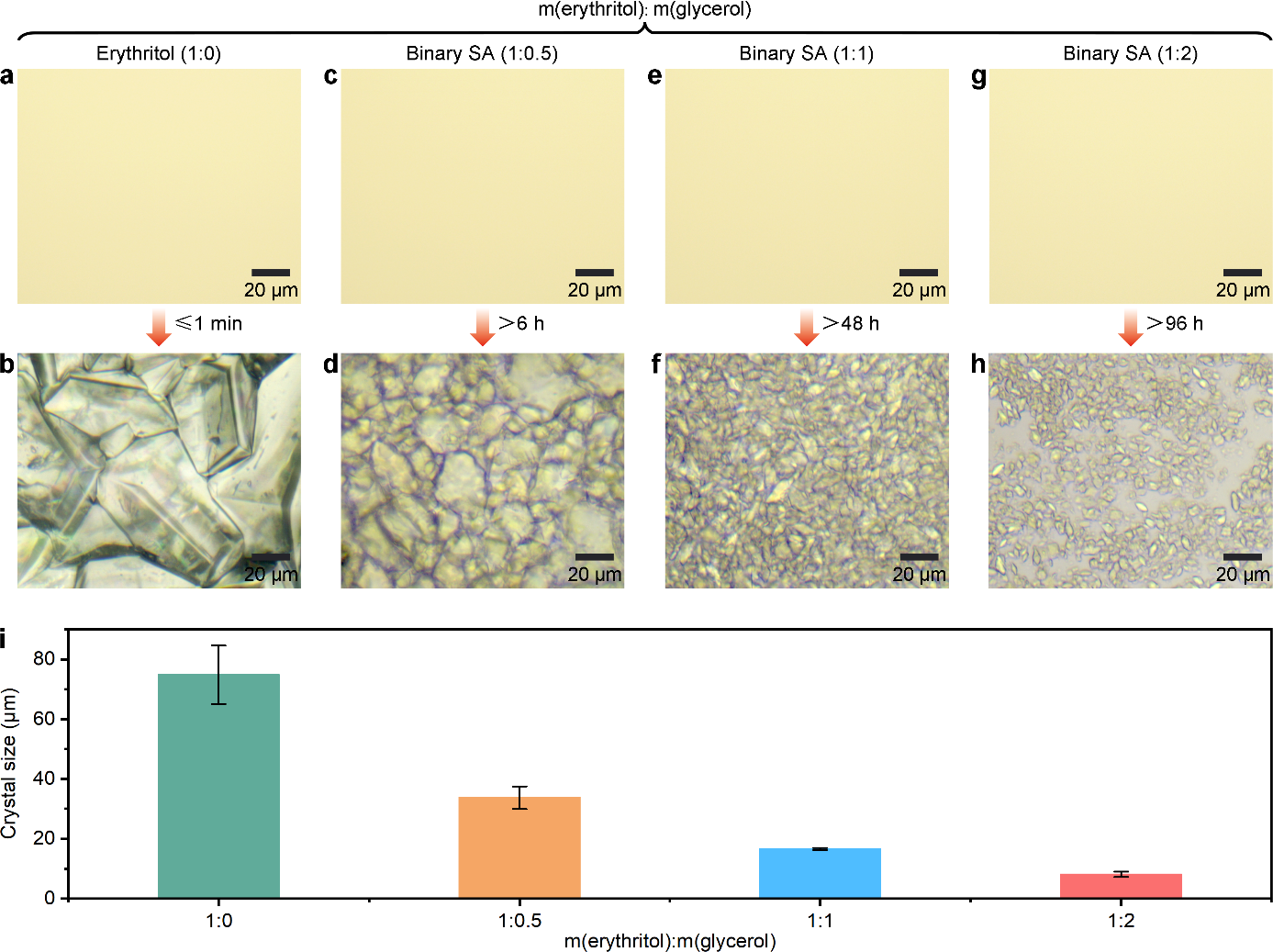


**Figure S1.** Optical microscope (OM) images of erythritol in the a) melted state and b) crystallized state. OM images of binary SA (1:0.5) in the c) melted state and d) crystallized state. OM images of binary SA (1:1) in the e) melted state and f) crystallized state. OM images of binary SA (1:2) in the g) melted state and h) crystallized state. i) Size of erythritol crystals at different glycerol content.


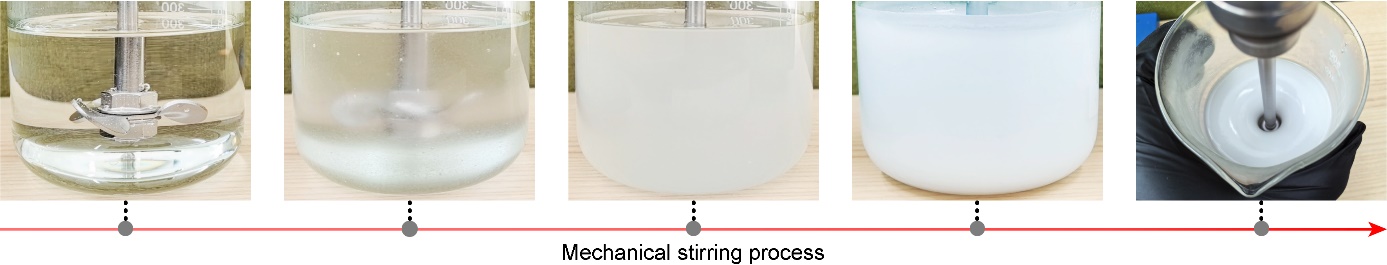


**Figure S2.** Controlled supercooling-crystallization phase change induced by mechanical stirring.

The implementation of mechanical stirring can effectively lower the nucleation energy barrier, which results in the controlled supercooling-crystallization phase change. With the gradual growth of erythritol crystals, the scattering of light at the interface between erythritol crystals and amorphous glycerol gradually increase. This phenomenon leads to a gradual decrease in the transmittance of binary SA.^[3]^ The final binary SA exhibits a milky white appearance.


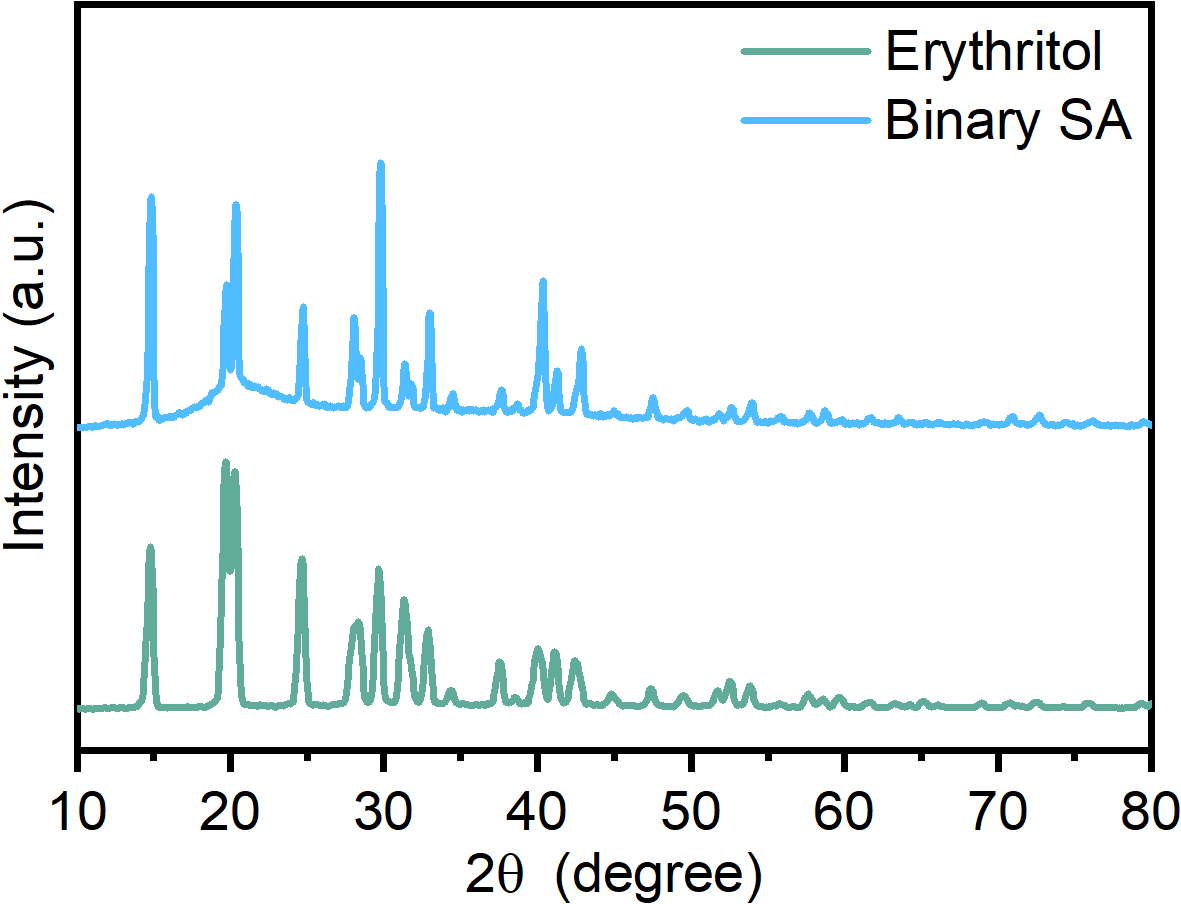


**Figure S3.** XRD patterns of erythritol and binary SA.

The XRD pattern of binary SA exhibits distinct diffraction peaks resembling those observed in the XRD pattern of erythritol. Consequently, the addition of glycerol prolongs the latent heat storage duration of amorphous erythritol below its crystallization temperature without affecting its phase change from amorphous state to crystallized state, which is in accordance with the observed crystal growth under OM.


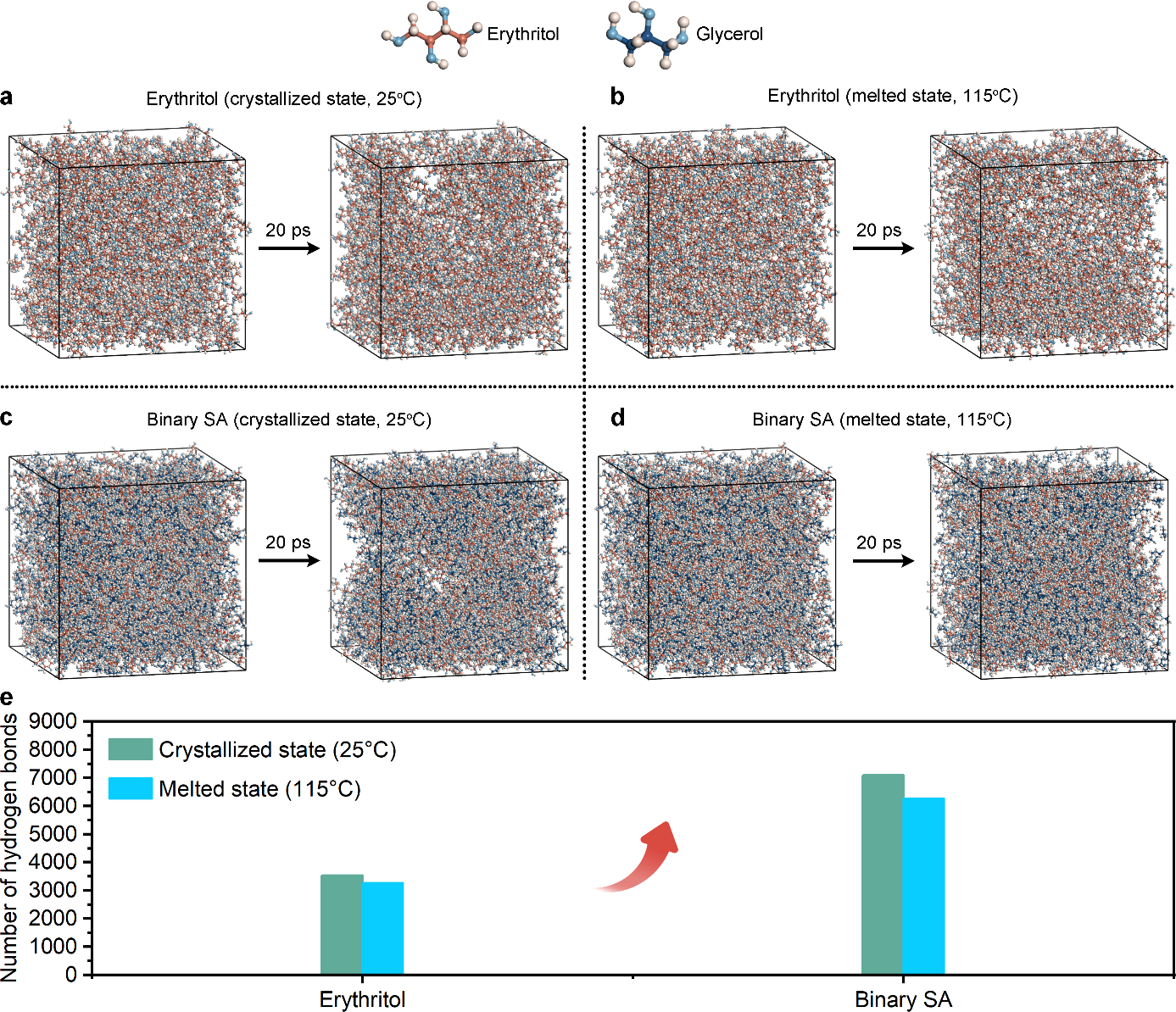


**Figure S4.** MD snapshots of erythritol in the a) crystallized state (25 ℃) and b) melted state (115 ℃). MD snapshots of binary SA in the c) crystallized state (25 ℃) and d) melted state (115 ℃). e) Number of hydrogen bonds in erythritol and binary SA as determined by MD simulations.

The molecular dynamics (MD) simulations show that the glycerol molecules are uniformly distributed among erythritol molecules, regardless of whether the binary SA is in the crystallized or melted state (**Figure S4a-d**, Supporting Information). Moreover, the number of hydrogen bonds in binary SA is significantly higher than that in erythritol following the addition of glycerol (**Figure S4e**, Supporting Information), which is consistent with the experimental results obtained from FT-IR analysis (Figure 2c). The formed molecular-scale hydrogen bonding networks delay the spontaneous crystallization of erythritol, thus achieving long-term latent heat storage.^[4, 5]^

**
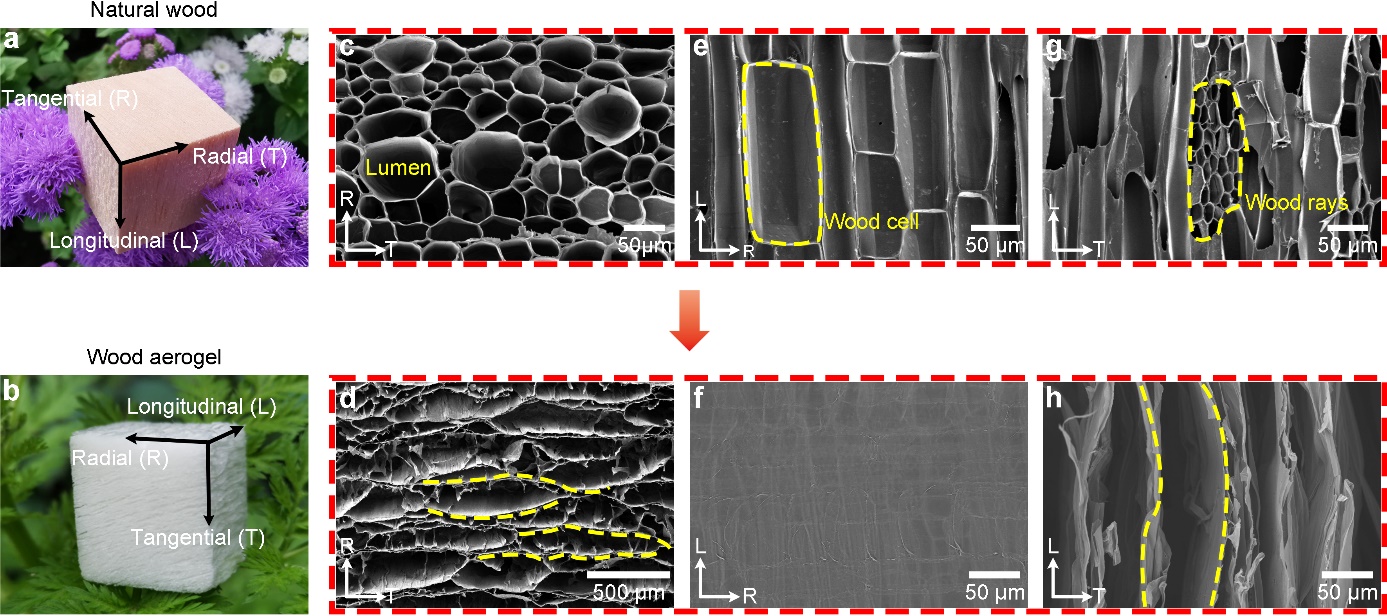
**

**Figure S5.** Photographs of a) natural wood and b) wood aerogel. Cross-section SEM images of c) natural wood and d) wood aerogel. Radial section SEM images of e) natural wood and f) wood aerogel. Tangential section SEM images of g) natural wood and h) wood aerogel.

The composition of natural wood primarily consists of three biomacromolecules, including cellulose, hemicellulose, and lignin.^[6]^ Notably, cellulose accounts for a significant proportion of 40%-50% in natural wood.^[7]^ Natural balsa wood is selected as a representative example in this study. The all-cellulose wood aerogel is prepared through the nearly complete removal of lignin and hemicellulose from natural wood. All-cellulose wood aerogel is referred to as wood aerogel in the following context.

Tangential, radial and longitudinal directions of the natural wood and wood aerogel are illustrated in **Figure S5 a, b** (Supporting Information). The microstructure differences between natural wood and wood aerogel are compared using scanning electron microscopy (SEM). After the almost complete removal of lignin and hemicellulose, the well-preserved cellulose framework is easily repelled and compressed along the wood rays by ice crystals during the freeze-drying process.^[8]^ Therefore, the honeycomb-like porous structure of natural wood in the cross-section undergoes a transformation into the wavy-like lamellar structure stacked layer by layer (**Figure S5c, d**, Supporting Information). The radial section of natural wood exhibits distinct channel-like structures, which results from interconnected hollow wood cells (**Figure S5e**, Supporting Information). In contrast, the radial section of wood aerogel shows obvious planar structure due to the destruction of wood cells (**Figure S5f**, Supporting Information). In tangential section, the wood rays of the natural wood are destroyed to form a lamellar structure resembling that observed in the cross-section (**Figure S5g, h**, Supporting Information).

**
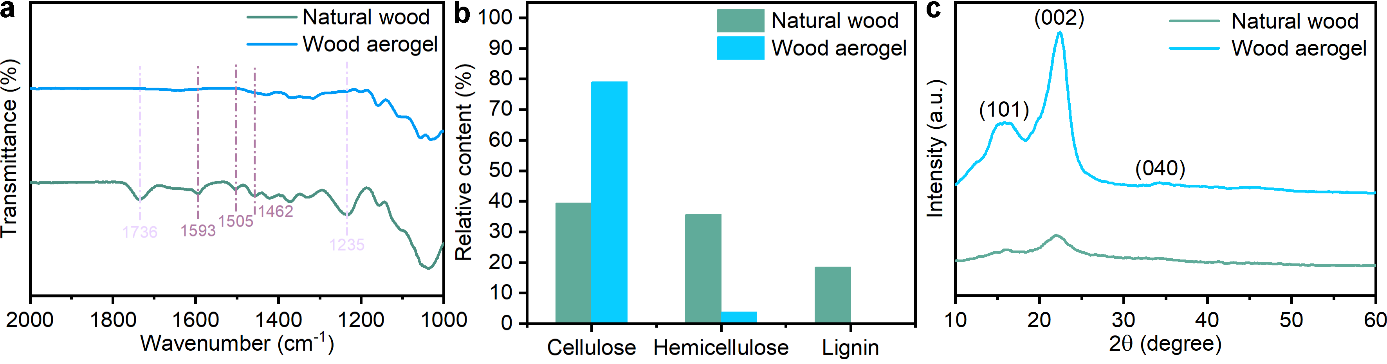
**

**Figure S6.** Comparison of a) FT-IR spectrum, b) chemical composition, and c) XRD pattern between natural wood and wood aerogel.

The Fourier transform infrared spectroscopy (FT-IR) spectrum of natural wood displays the characteristic absorption peaks associated with lignin at 1593 cm^-1^, 1505 cm^-1^, and 1462 cm^-1^ (**Figure S6a**, Supporting Information). Additionally, the characteristic absorption peaks at 1736 cm^-1^ and 1235 cm^-1^ belong to hemicellulose in natural wood. These characteristic peaks are nearly absent in the FT-IR spectrum of wood aerogel, indicating that cellulose is the predominant constituent of wood aerogel.^[9]^ The chemical composition analysis further confirms cellulose as the predominant constituent of wood aerogel (**Figure S6b**, Supporting Information). The crystal structure of cellulose in natural wood and wood aerogel is characterized using X-ray diffractometer (XRD) pattern (**Figure S6c**, Supporting Information). The diffraction patterns of both wood samples display characteristic peaks at 16.8°(101), 22.5°(002), and 34.9°(040) following the removal of lignin and hemicellulose, indicating that the cellulose crystal structure undergoes minimal changes.^[10]^

**
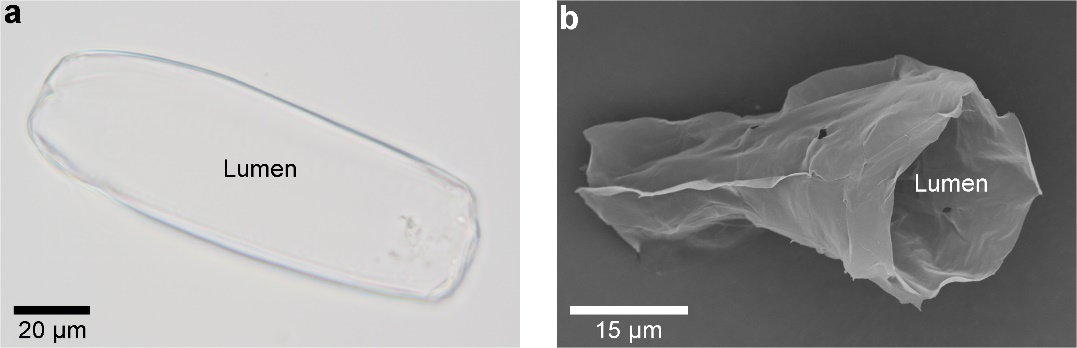
**

**Figure S7.** a) OM image and b) SEM image of a single cellulose fiber separated from wood aerogel.


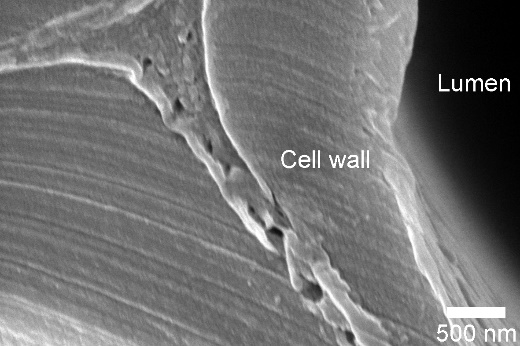


**Figure S8.** SEM image of natural wood cell wall.

Compared to the wood aerogel cell wall, the natural wood cell wall exhibits a lower degree of exposed cellulose nanofibers. (**Figure 2e**; **Figure S8**, Supporting Information).


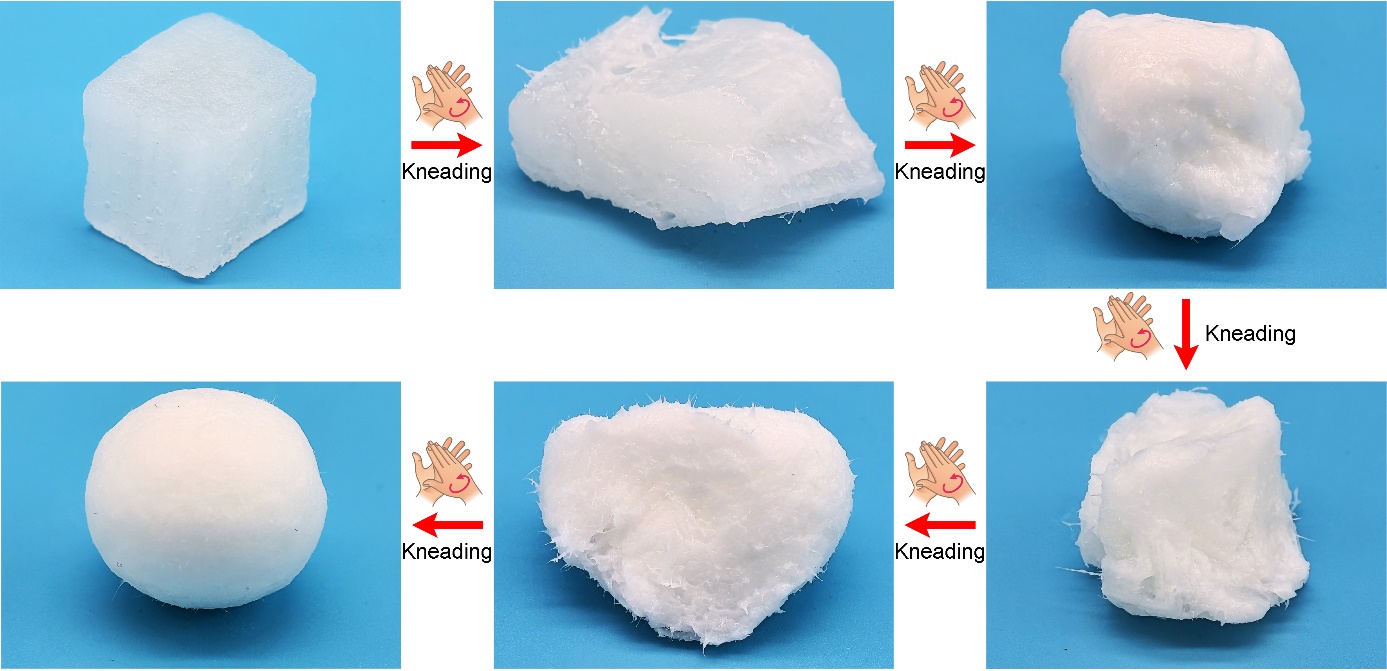


**Figure S9.** Kneading process for preparing a wood plasticine without photothermal ability.

A wood plasticine without photothermal ability is prepared by infiltrating binary SA into a wood aerogel, followed by thorough kneading.

In this study, natural wood is chosen as the raw material for cellulose extraction over other plants due to its high cellulose content, hierarchical porous structure, sustainability, and widespread global availability.^[6, 7]^ By nearly completely removing lignin and hemicellulose from natural wood, an all-cellulose wood aerogel that retains the native hierarchical porosity can be obtained. Consequently, utilizing wood aerogel as a base material for the preparation of wood plasticine not only imparts good plasticity to the resulting product but also retains the inherent advantages of wood aerogel, namely its sustainability and abundant availability. Moreover, the retained hierarchical porous structure facilitates the uniform impregnation of PCMs.


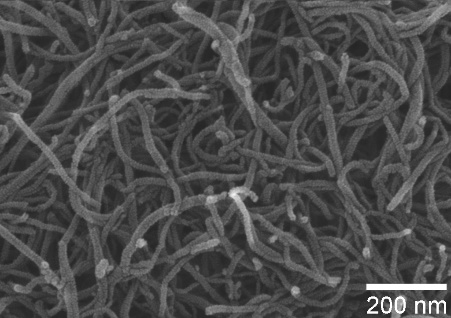


**Figure S10.** SEM image of CNTs.


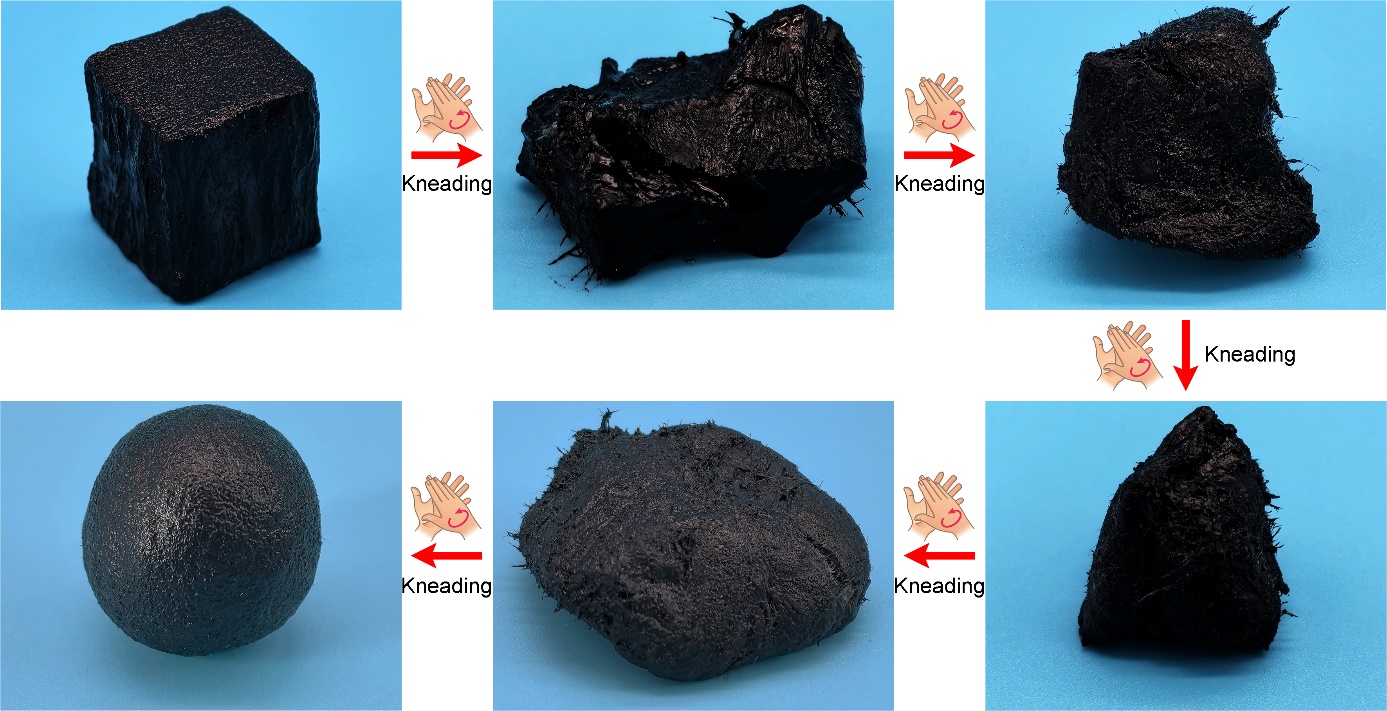


**Figure S11.** Kneading process for preparing a photothermal wood plasticine.

A photothermal wood plasticine is prepared by infiltrating CNTs-doped binary SA into a wood aerogel, followed by thorough kneading.

Actually, increasing the CNTs content in the binary SA can also transform the CNTs-doped binary SA into a plasticine-like material, owing to the abundance of surface hydroxyl groups on the CNTs. However, the synthesis and hydroxylation of CNTs involve high energy consumption, the use of toxic catalysts, and complex post-treatment processes. Moreover, CNTs are prone to aggregation, which hinders the effective impregnation of PCMs. In contrast, wood aerogel offers distinct advantages, such as sustainability, an inherent hierarchical porous structure that facilitates the homogeneous impregnation of PCMs, and abundant natural hydroxyl groups. Consequently, considering these benefits, we select wood aerogel as the matrix material.


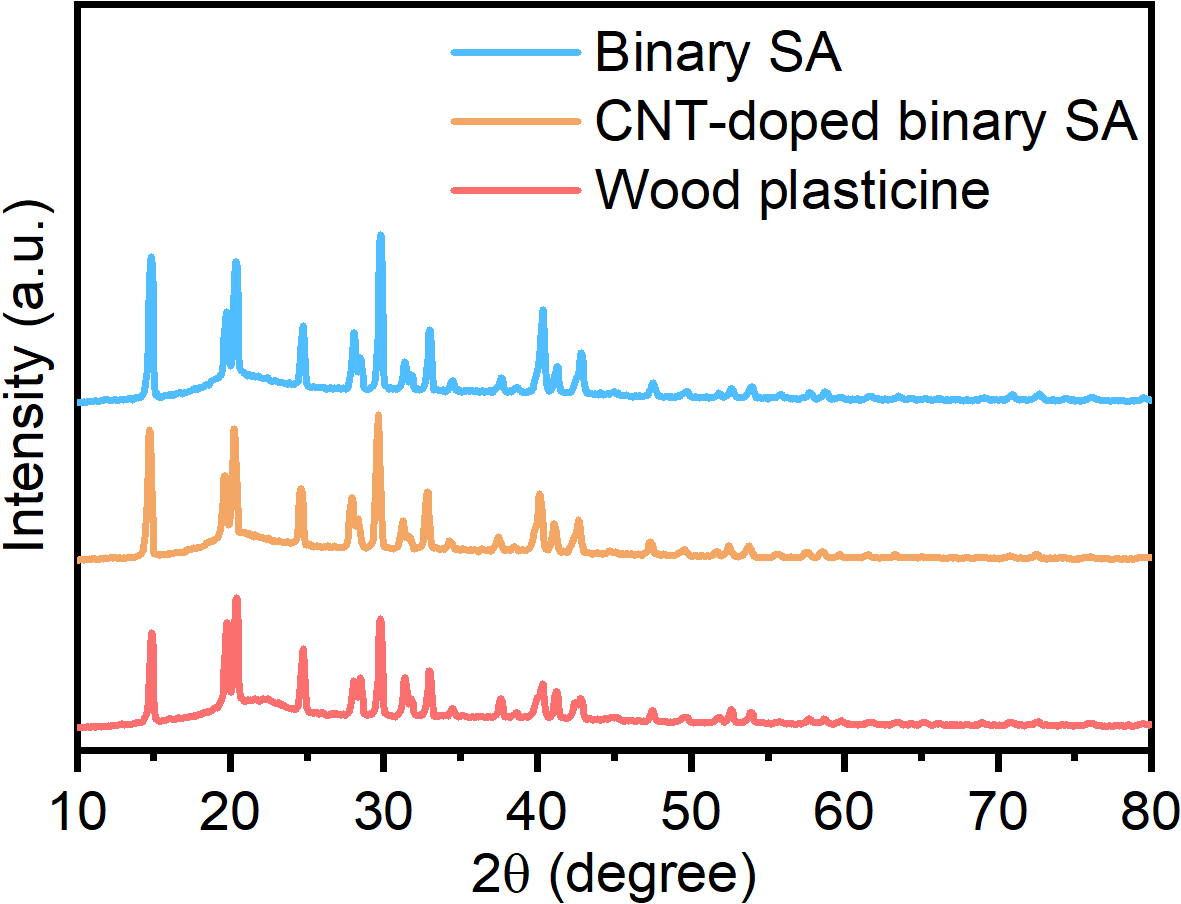


**Figure S12.** XRD patterns of binary SA, CNTs-doped binary SA, and wood plasticine.

The XRD pattern of CNTs-doped binary SA displays obvious diffraction peaks which closely resemble those observed in the XRD pattern of binary SA, suggesting that incorporating CNTs as a photothermal conversion additive has negligible influence on the crystal structure of binary SA. Furthermore, the XRD pattern of wood plasticine also displays obvious diffraction peaks that are similar to those observed in the XRD pattern of CNTs-doped binary SA. Consequently, the CNTs-doped binary SA is fully encapsulated within the hierarchically porous structure of cellulose fibers separated from wood aerogel, enabled by purely physical interactions.

**
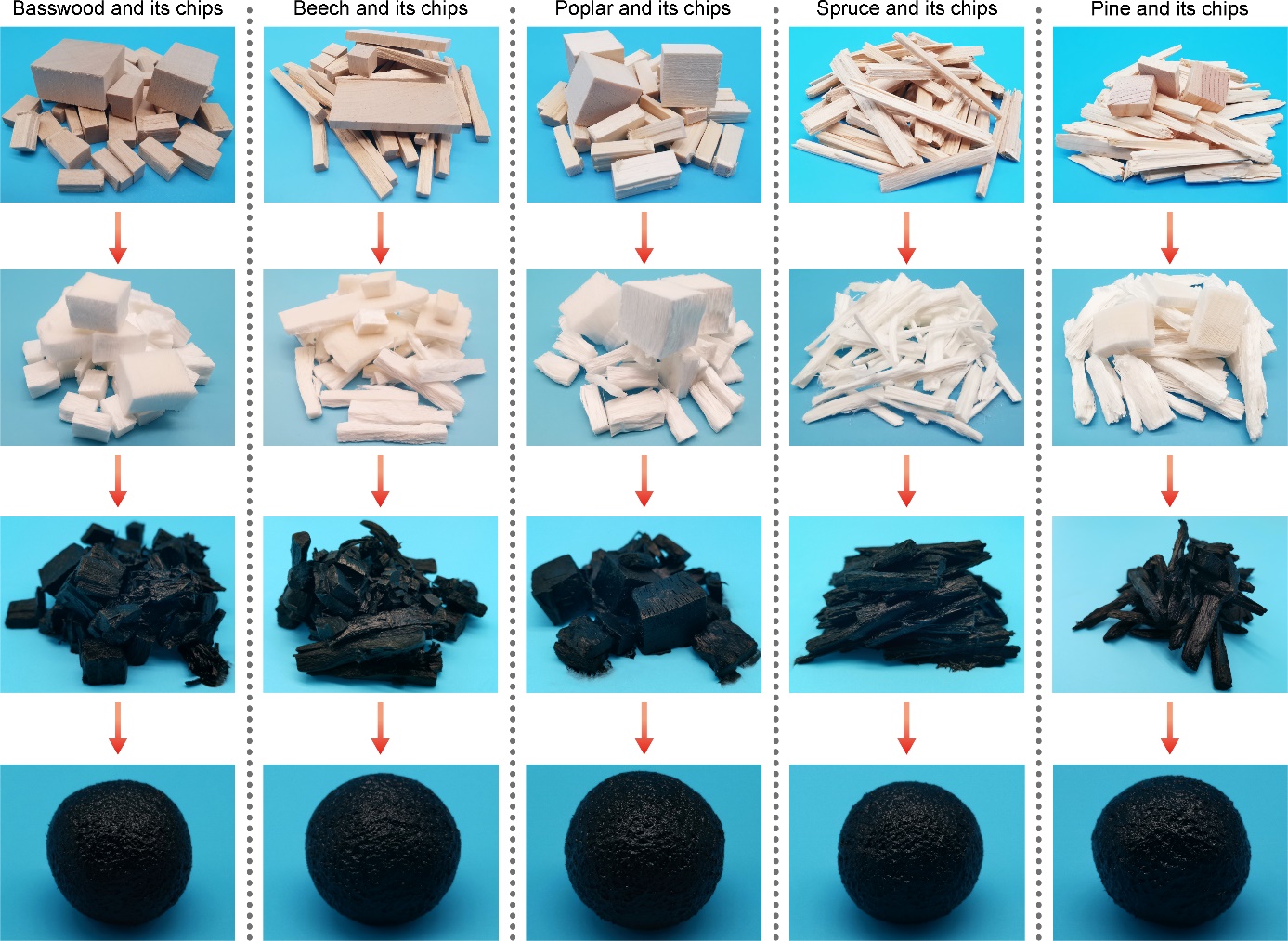
**

**Figure S13.** Photographs illustrating the fabrication process of wood plasticine derived from various wood types (basswood, beech, poplar, spruce, pine) and their chips. The process, similar to that employing balsa wood, consists of three key steps: near-total delignification and hemicellulose removal, followed by sufficient impregnation of CNTs-doped binary SA, and final mechanical kneading.

**
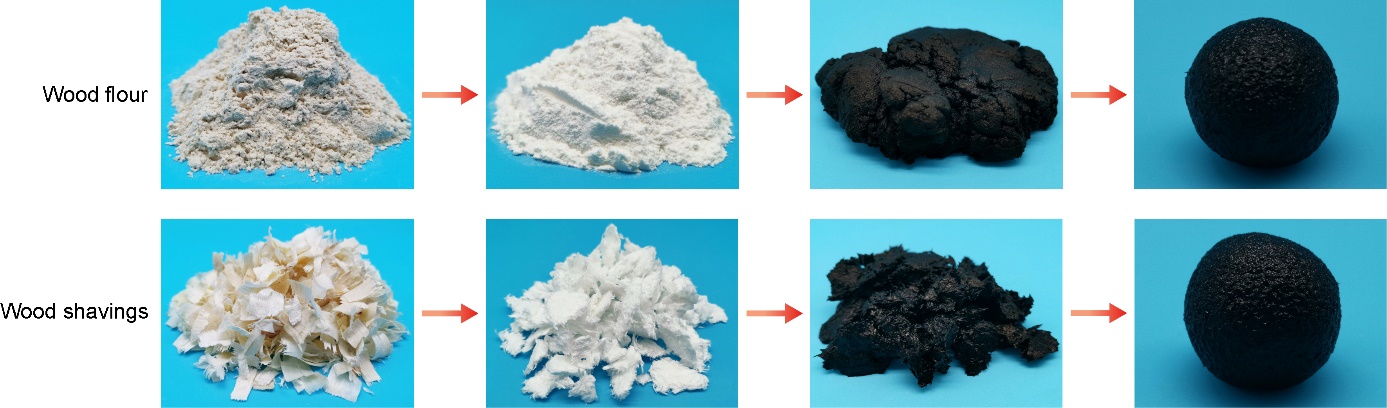
**

**Figure S14.** Photographs illustrating the fabrication process of wood plasticine derived from wood flours and shavings.

**
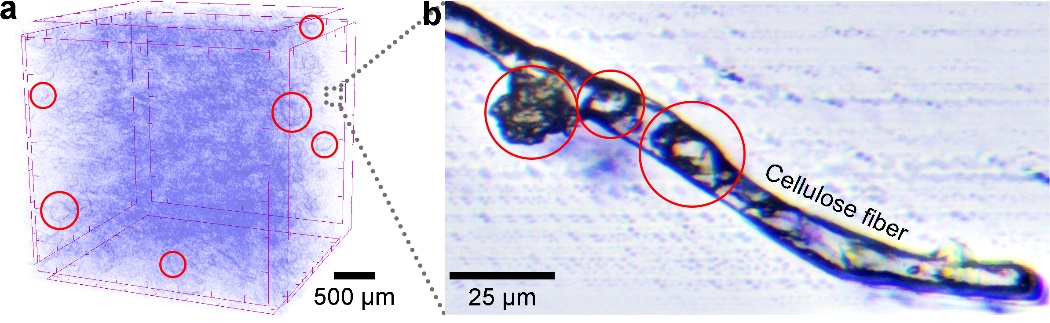
**

**Figure S15.** a) 3D reconstruction of wood plasticine derived from X-ray microtomography. b) OM image of a single cellulose fiber separated from wood plasticine.

The red circles in **Figure S15a, b** (Supporting Information) denotes erythritol crystals. A substantial quantity of cellulose fibers (purple) is uniformly dispersed within the erythritol crystals and the amorphous glycerol (**Figure S15a**, Supporting Information). Additionally, smaller erythritol crystals are observed within the lumen of a single cellulose fiber (**Figure S15b**, Supporting Information).

**
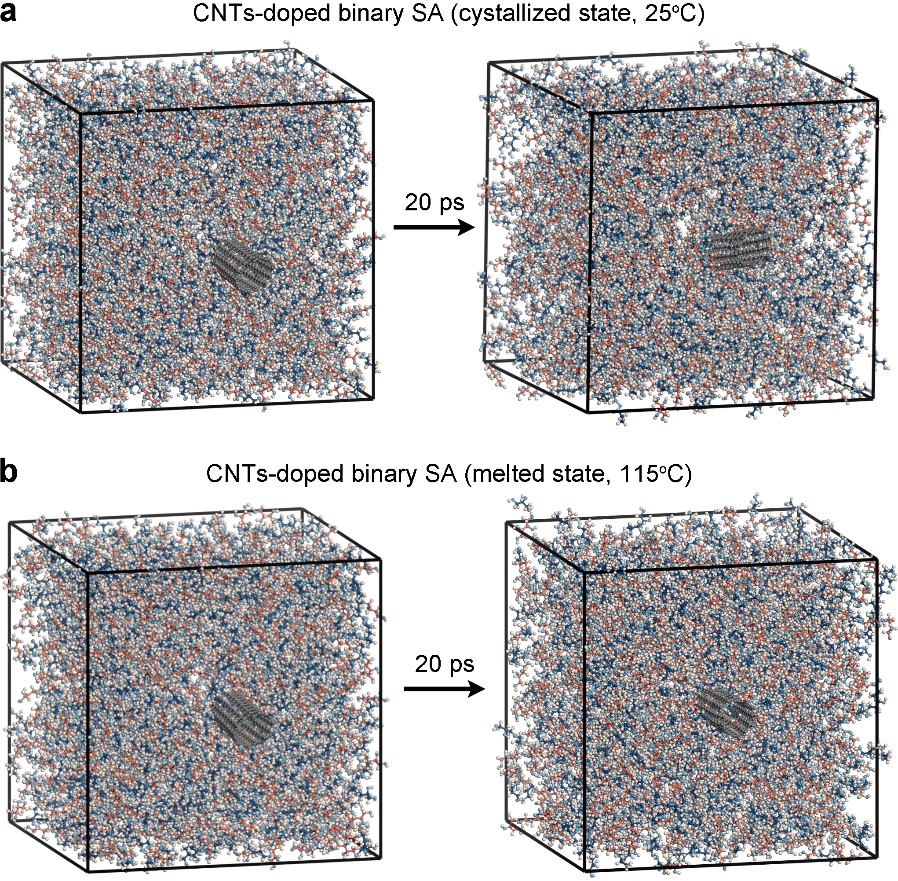
**

**Figure S16.** MD Snapshots of CNTs-doped binary SA in the a) crystallized state (25 ℃) and b) melted state (115 ℃). Note:To emphasize the carbon nanotube, the spatial position of the carbon nanotube is adjusted appropriately.

**
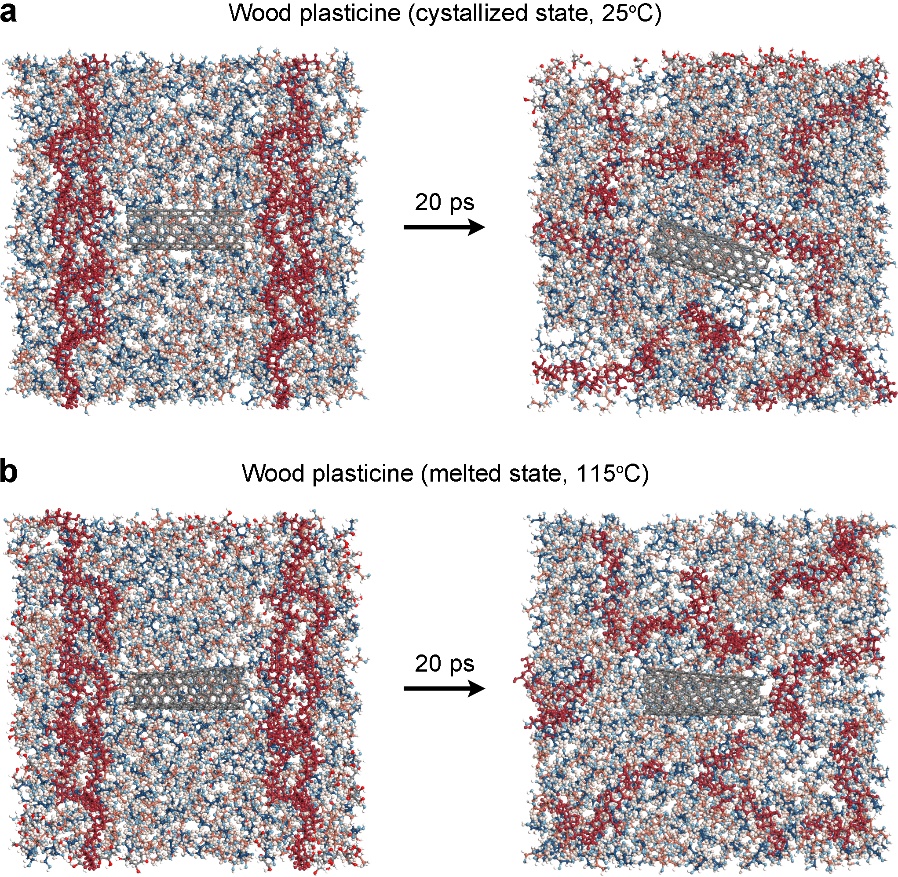
**

**Figure S17.** MD Snapshots of wood plasticine in the a) crystallized state (25 ℃) and b) melted state (115 ℃).

Cellulose molecules are uniformly dispersed in crystallized and melted CNTs-doped binary SA.

**
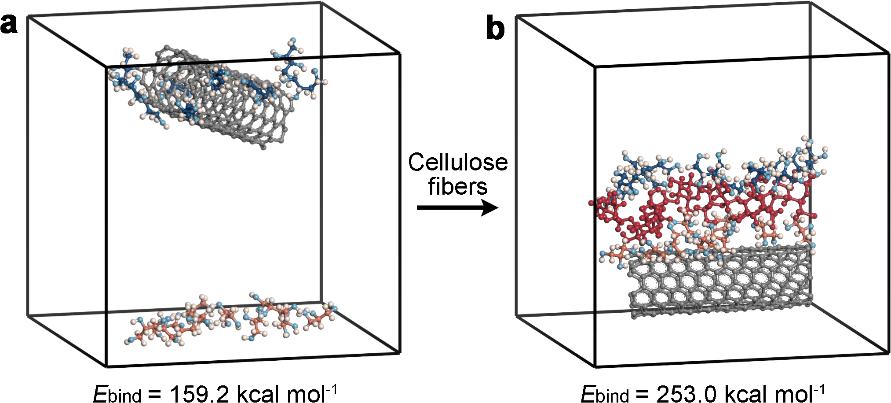
**

**Figure S18.** Binding energy simulation models of a) CNTs-doped binary SA and b) wood plasticine after MD simulation.

To more clearly illustrate the binding interactions among erythritol, glycerol, and cellulose fibers while considering that CNTs do not participate in hydrogen bond formation, the CNTs are hidden in the simulated model presented in **Figure 2j, k**. After optimizing the molecular structures and achieving conformational stabilization, the binding energy is calculated by employing Equations (1) and (2):

$\text{E}_{\text{bind}\text{ }\text{(CNTs-doped binary SA)}}\text{ = }\text{E}_{\text{erythritol}}\text{ + }\text{E}_{\text{glycerol}}\text{ + }\text{E}_{\text{CNTs}} \text{-}\text{ }\text{E}_{\text{total}}$ (1)

$\text{E}_{\text{bind (wood }\text{plasticine}\text{)}}\text{ = }\text{E}_{\text{erythritol}}\text{ + }\text{E}_{\text{glycerol}}\text{ + }\text{E}_{\text{CNTs}} \text{+} E_{\text{cellulose fibers}} \text{- }\text{E}_{\text{total}}$ (2)

where *E*_bind_ represents the binding energy among the various constituents within the entire material system, while *E*_erythritol_, *E*_glycerol_, *E*_CNTs_, and *E*_cellulose fibers_ denote the respective energies of erythritol, glycerol, CNTs, and cellulose fibers. Additionally, *E*_total_ signifies the total energy of the corresponding material system.

**
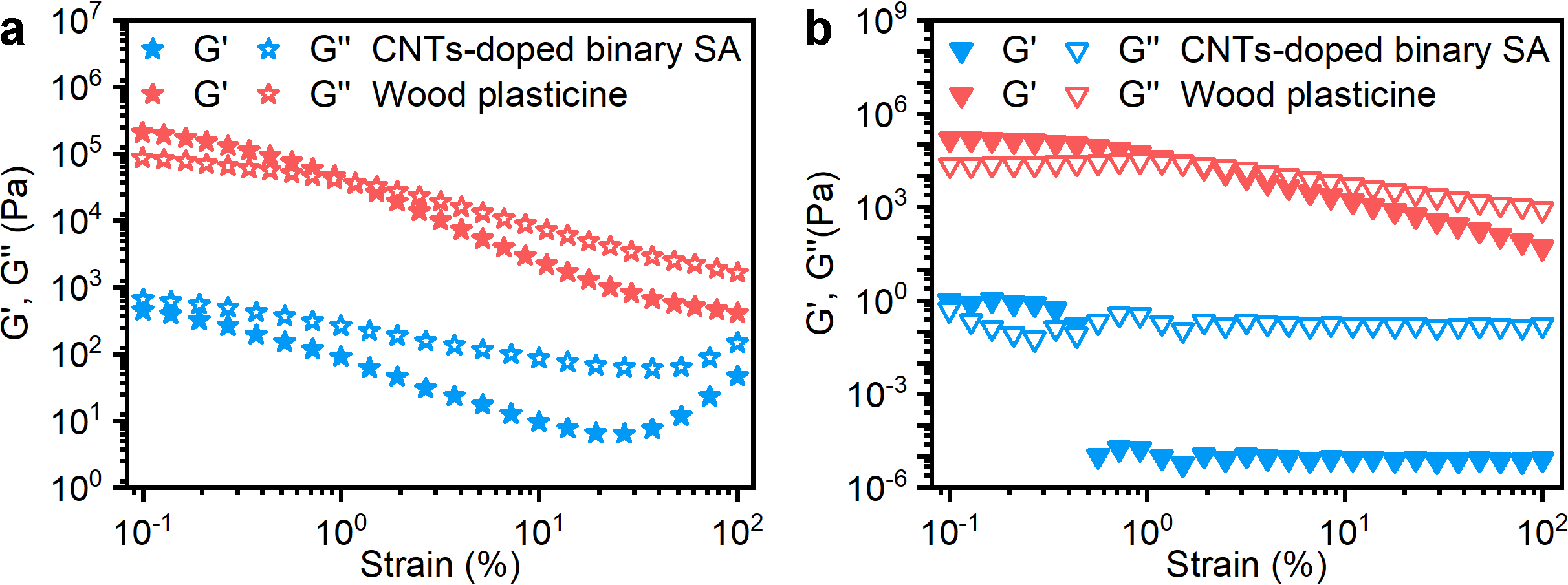
**

**Figure S19.** Strain sweep measurements of CNTs-doped binary SA and wood plasticine in the a) crystallized state (25 ℃) and b) melted state (115 ℃).

**
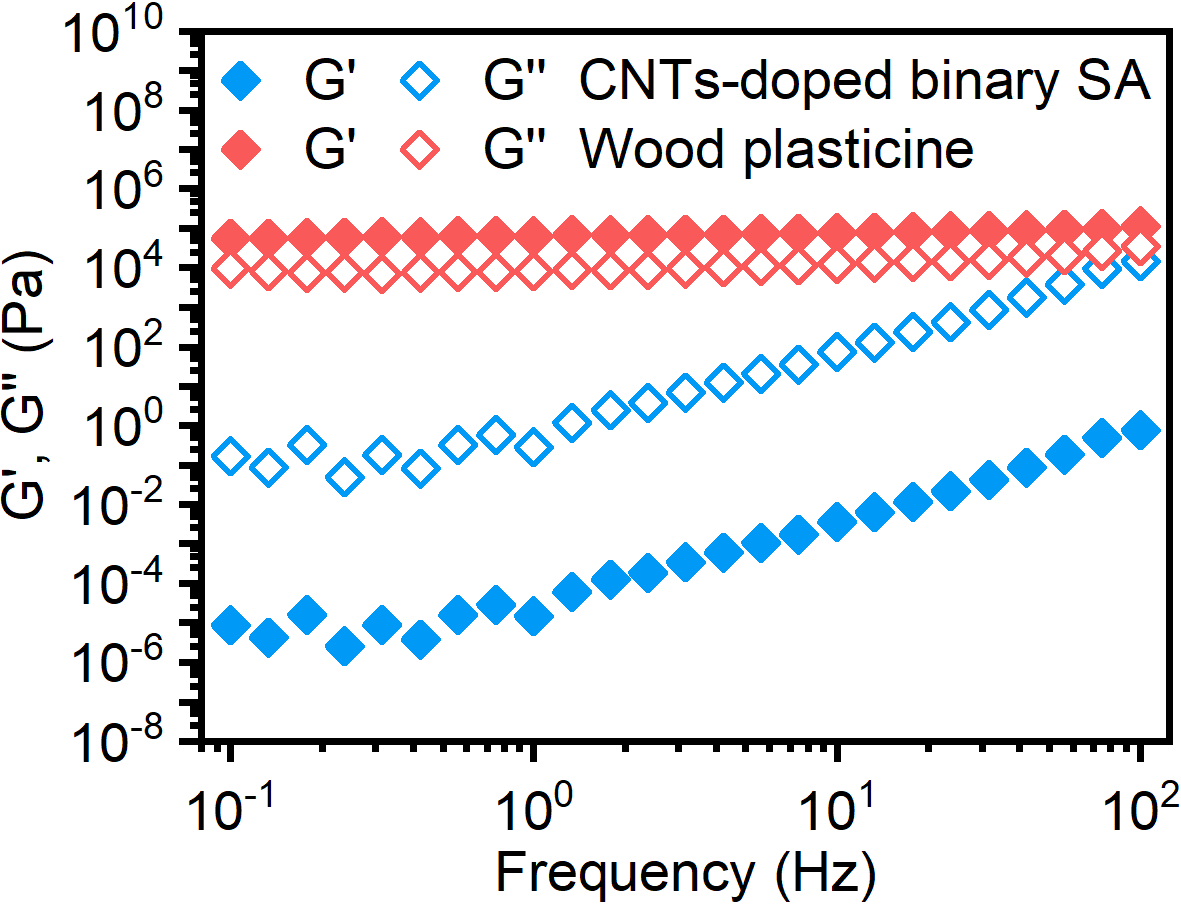
**

**Figure S20.** Frequency sweep measurements of CNTs-doped binary SA and wood plasticine in the melted state (115 ℃).


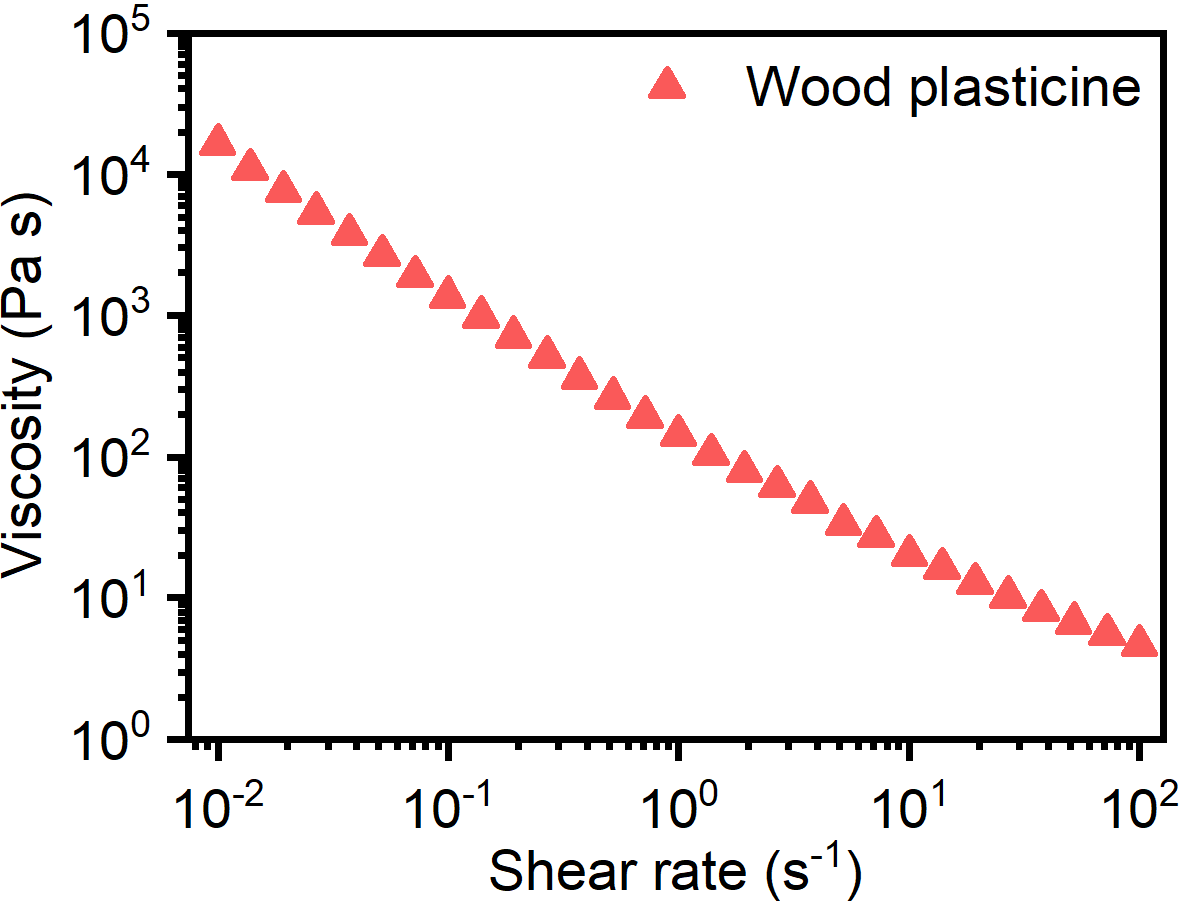


**Figure S21.** Shear rate sweep of wood plasticine in the melted state (115 ℃).

**
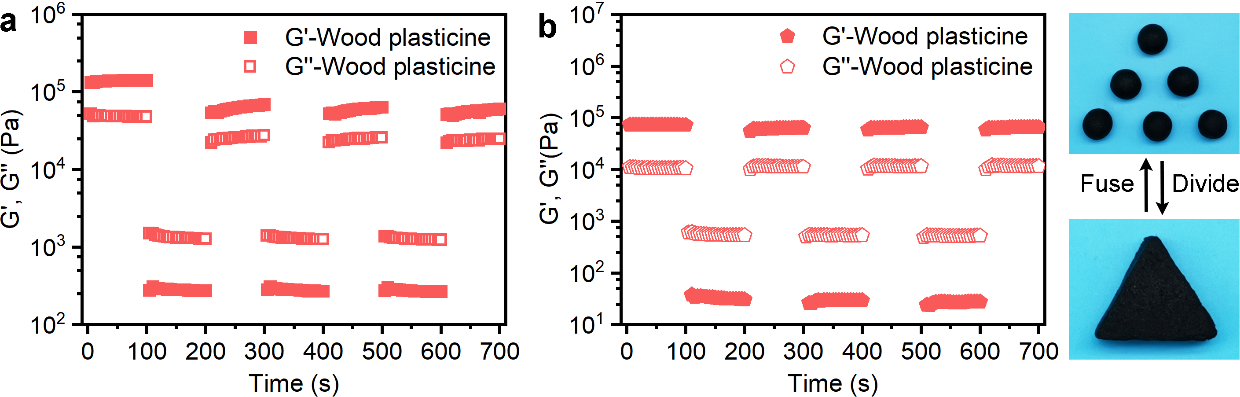
**

**Figure S22** Continuous step strain measurements of wood plasticine in the a) crystallized state (25 ℃) and b) melted state (115 ℃). Photographs demonstrating the self-healing property of wood plasticine.

The self-healing property is attributed to the multiscale hydrogen bonding interactions within wood plasticine.^[11]^ The self-healing time is assessed via continuous step-strain measurements performed over three shearing cycles, with strains varying from 0.1% to 50%. The results indicate that G’ is higher than G’’ at a strain of 0.1%, while G’’ surpasses G’ at a strain of 50%. The reversible conversion between G’ and G’’ occurs instantaneously during the shearing cycle, indicating the rapid self-healing capability of fractured wood plasticine.

**
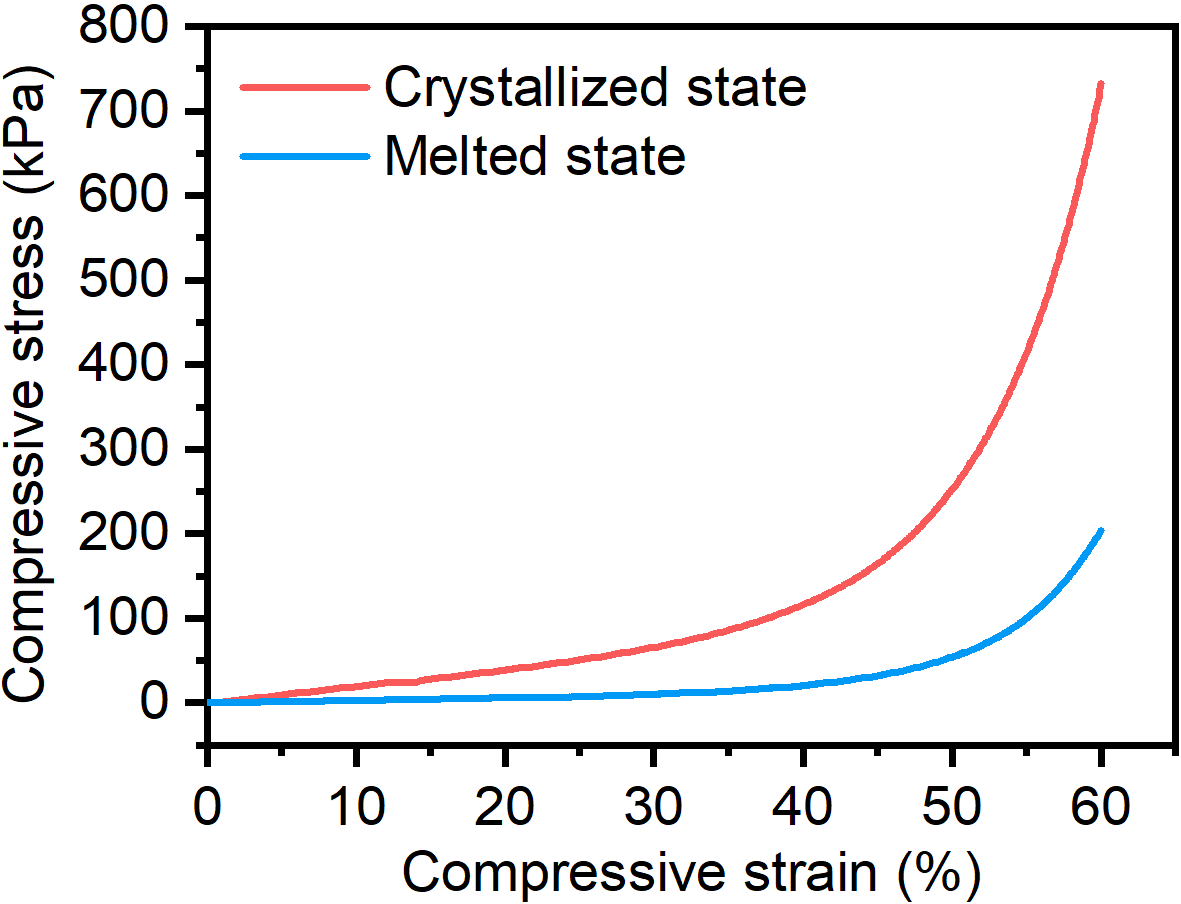
**

**Figure S23.** Compressive stress-strain curves of wood plasticine in the crystallized and melted states.

**
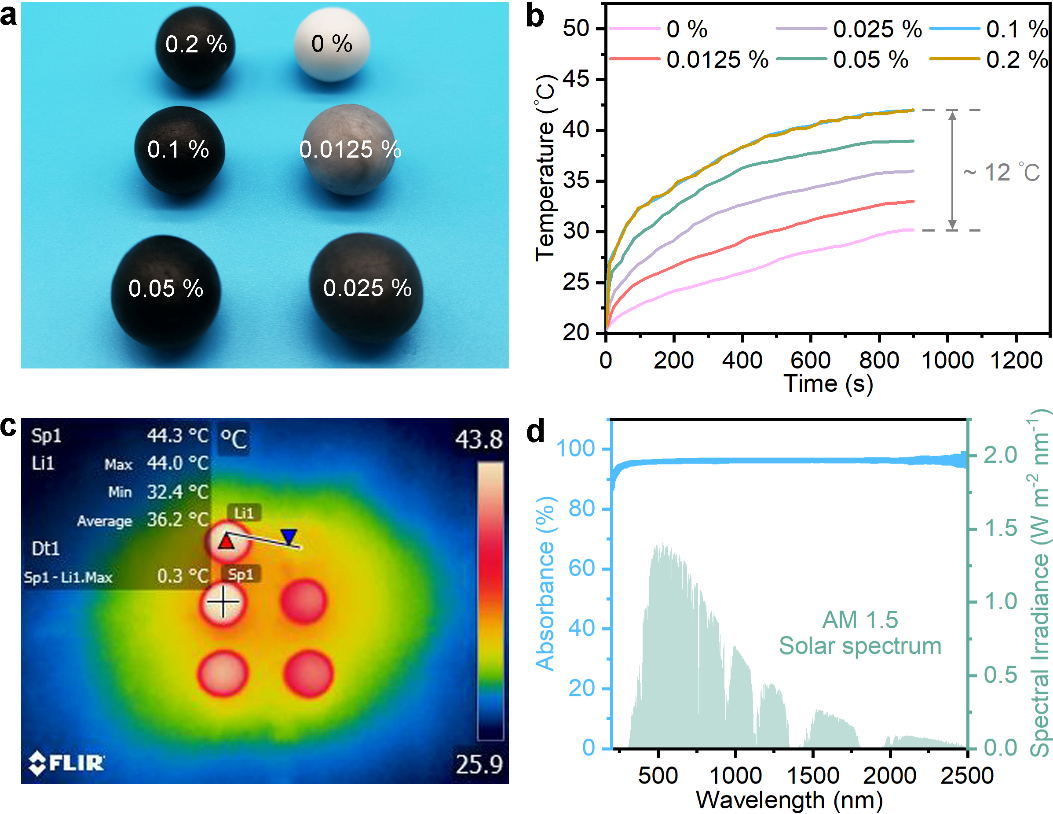
**

**Figure S24.** a) Photograph of wood plasticine with varying mass fractions of CNTs. b) Time-temperature curves and c) Infrared thermal image of wood plasticine with varying mass fractions of CNTs under one solar intensity irradiation. d) UV-VIS-NIR spectra of wood plasticine containing 0.1% mass fraction of CNTs; The grey shaded area represents the standard AM 1.5 solar spectrum.

Under one solar intensity irradiation, the temperature of wood plasticine gradually increases as the mass fraction of CNTs rises from 0% to 0.2%, indicating a progressive enhancement in its photothermal conversion ability (**Figure S24a, b**, Supporting Information). When the mass fraction of CNTs is either 0.1% or 0.2%, the maximum temperature of the wood plasticine consistently reaches 43.8 ℃ (**Figure S24b, c**, Supporting Information). The observed phenomenon is attributed to the transition of CNTs in wood plasticine from uniform dispersion to significant aggregation as the mass fraction of CNTs increases. The addition of CNTs can increase the temperature of pure wood plasticine by up to 12 ℃. In addition, the wood plasticine exhibits nearly 100% light absorption over the entire solar spectrum from 200 to 2500 nm, which is consistent with its excellent photothermal conversion ability (**Figure S24d**, Supporting Information).


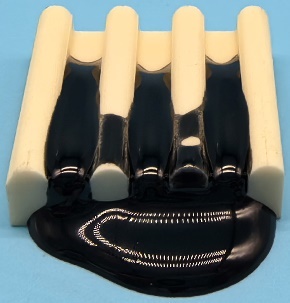


**Figure S25.** The flow behavior of CNTs-doped binary SA containing only molecular-scale hydrogen bonding networks on arch-like rough surface.

**
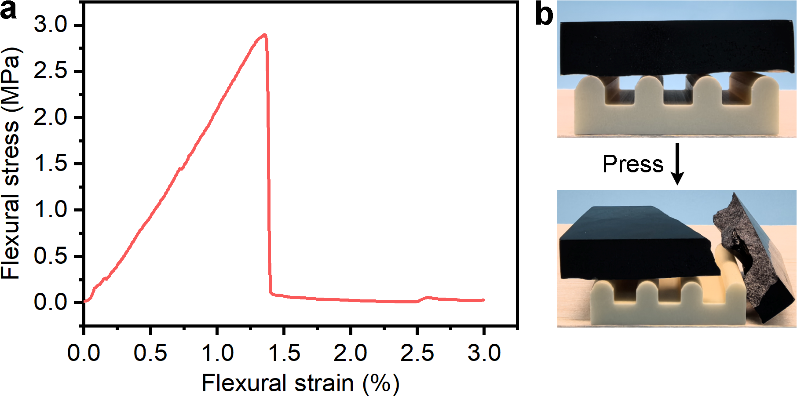
**

**Figure S26** a) Flexural stress–strain curve of glycerol-free wood plasticine incorporating only nano/micro-scale hydrogen bonding networks, as obtained from three-point test. b) Photographs illustrating the near-brittle fracture characteristic of glycerol-free wood plasticine on the arch-like rough surface under external pressure.

The glycerol-free wood plasticine incorporating only nano/micro-scale hydrogen bonding networks shows a flexural modulus of 175.3 ± 9.1 MPa and a flexural strength of 2.9 ± 0.3 MPa (**Figure S26a**, Supporting Information). Moreover, this glycerol-free wood plasticine exhibits a smooth extension fracture path that is nearly parallel to the applied stress, indicating its near-brittle fracture characteristic (**Figure S26b**, Supporting Information).

**
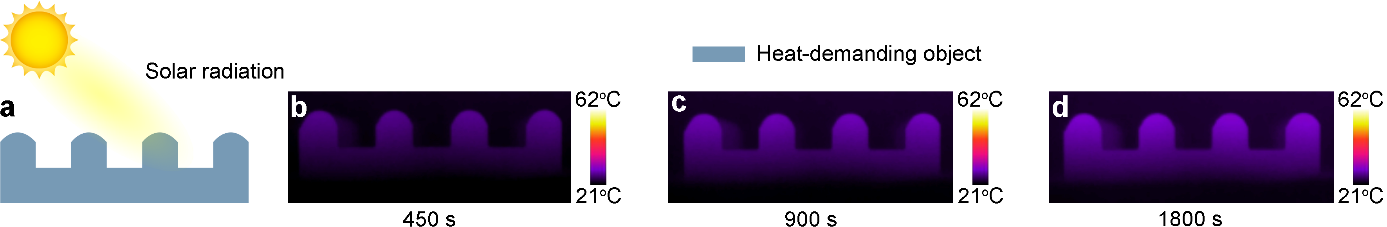
**

**Figure S27.** a) Schematic diagram of heat-demanding object uncoated with glycerol-free wood plasticine or wood plasticine under solar radiation. Infrared thermal images of heat-demanding object under varied durations of solar radiation: b) 450 s, c) 900 s, and d) 1800 s.

**
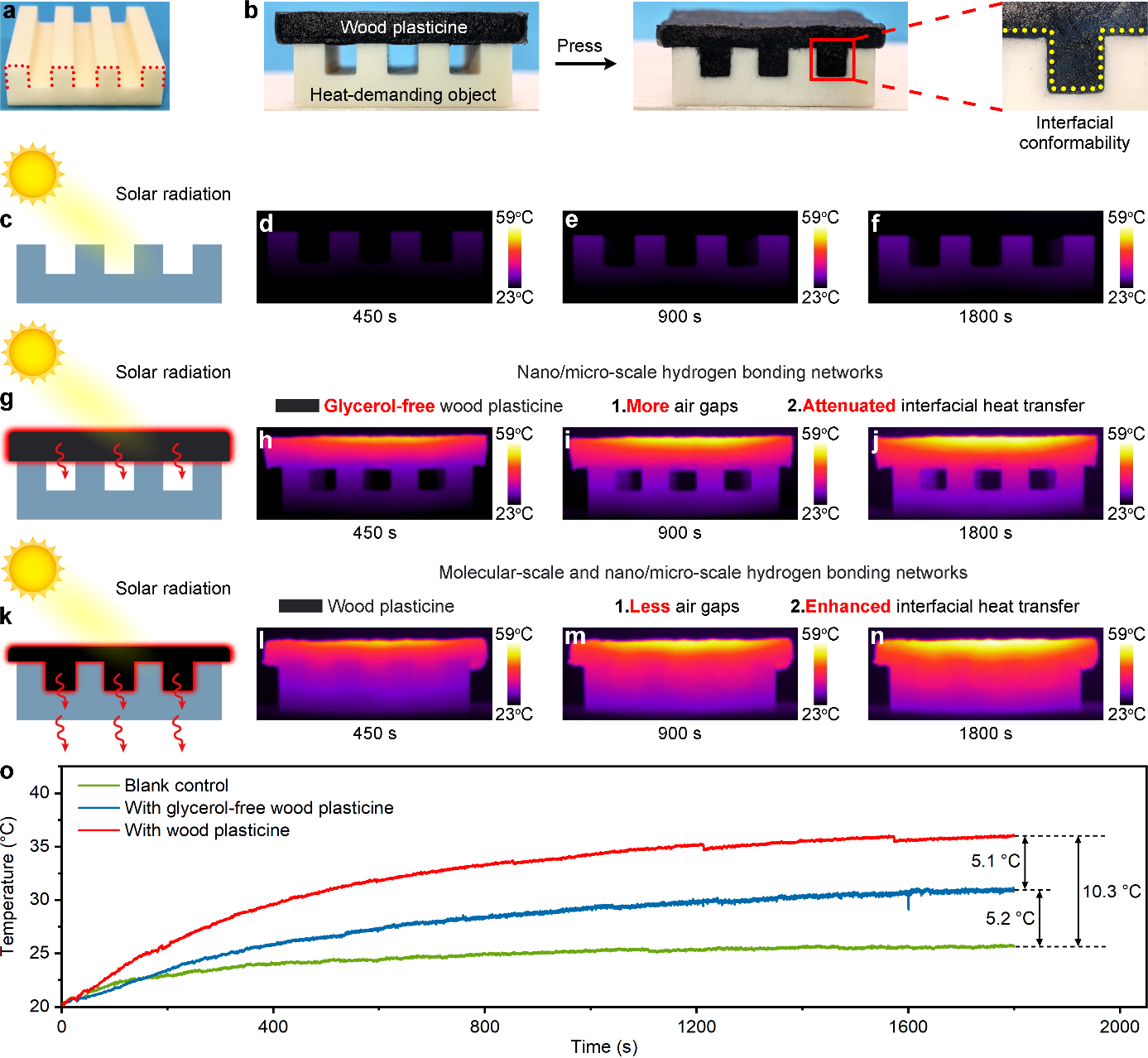
**

**Figure S28.** a) Photograph of heat-demanding object with square-like rough surface. b) Photographs illustrating conformal contact between wood plasticine and heat-demanding object under external pressure. c) Schematic diagram of heat-demanding object uncoated with glycerol-free wood plasticine or wood plasticine under solar radiation. Infrared thermal images of heat-demanding object under varied durations of solar radiation: d) 450 s, e) 900 s, and f) 1800 s. g) Schematic diagram of heat-demanding object coated with glycerol-free wood plasticine under solar radiation. Infrared thermal images of heat-demanding object coated with glycerol-free wood plasticine under varied durations of simulated solar radiation: h) 450 s, i) 900 s, and j) 1800 s. k) Schematic diagram of heat-demanding object coated with wood plasticine under solar radiation. Infrared thermal images of heat-demanding object coated with wood plasticine under varied durations of simulated solar radiation: l) 450 s, m) 900 s, and n) 1800 s. o) Time-temperature curves of heat-demanding object uncoated with glycerol-free wood plasticine or wood plasticine, coated with glycerol-free wood plasticine, and coated with wood plasticine under simulated solar radiation.

The malleable wood plasticine, possessing hydrogen bonding networks at both the molecular and nano/micro scales, exhibits remarkable conformal adaptability to the square-like rough surface of heat-demanding object under external compression (**Figure S28a, b**, Supporting Information). As illustrated in the infrared thermal images, both glycerol-free wood plasticine and wood plasticine successfully converts simulated solar energy into solar heat, enabling targeted heating of the heat-demanding object (**Figure S2****8c-n**, Supporting Information). As the duration of solar illumination increases, the average temperature of heat-demanding object gradually rises and ultimately stabilizes at a constant value after 1800 seconds (**Figure S28o**, Supporting Information). The application of glycerol-free wood plasticine and wood plasticine can elevate the average temperature of bare heat-demanding object by 5.2 ℃ and 10.3 ℃, respectively. Additionally, the average temperature of the heat-demanding object coated with wood plasticine is 5.1 ℃ higher than that of the object coated with glycerol-free wood plasticine.

**
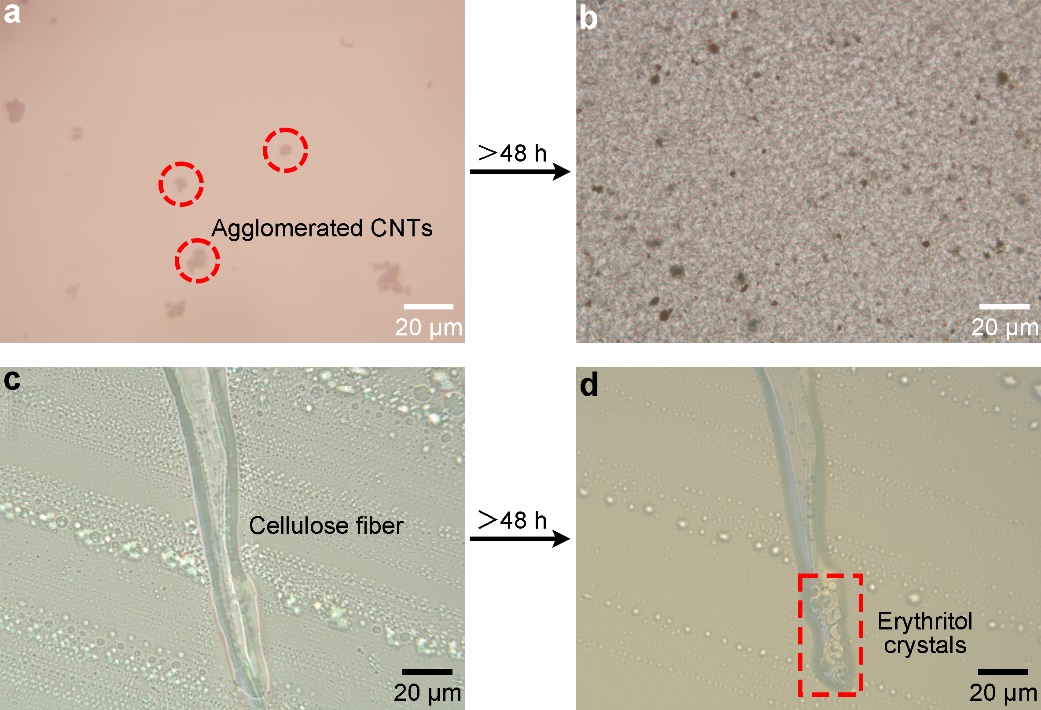
**

**Figure S29.** OM images of a) supercooled CNTs-doped binary SA, b) crystallized CNTs-doped binary SA, c) a single cellulose fiber separated from supercooled wood plasticine, and d) a single cellulose fiber separated from wood plasticine during supercooling-crystallization phase change.

The incorporation of CNTs and cellulose fibers has a negligible effect on the long-term latent heat storage properties of binary SA at room temperature. After 48 hours, a substantial amount of erythritol crystals is observed to form within the CNTs-doped binary SA and the wood plasticine at room temperature (**Figure S29a-d**, Supporting Information). The latent heat storage duration is still maintained at 48 hours.


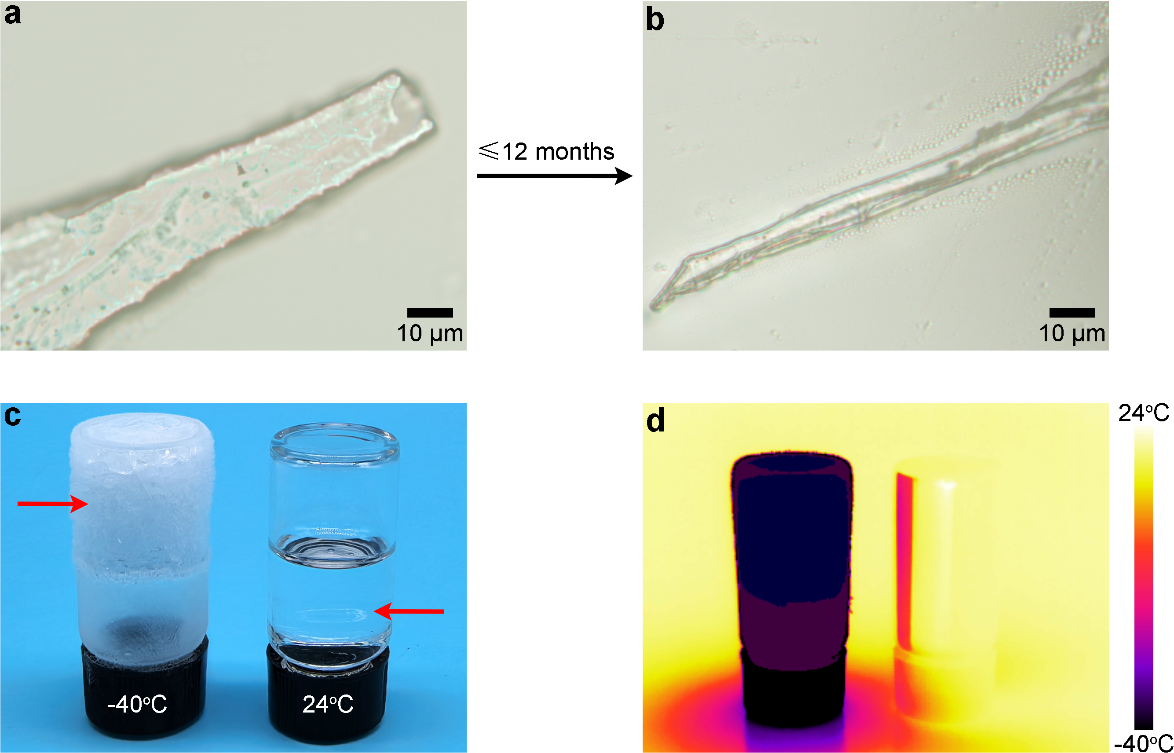


**Figure S30.** OM images of a) a single cellulose fiber separated from supercooled wood plasticine and b) another single cellulose fiber separated from the same supercooled wood plasticine after 12 months of storage at -40 ℃. c)Photograph illustrating the binary SA encapsulated in glass bottles stored at -40 ℃ and 24 ℃; The red arrows indicate the positions of binary SA at different temperatures. d) Corresponding infrared thermal image obtained from c).

When the ambient temperature is -40 ℃, the wood plasticine can maintain long-term latent heat storage for a duration of 12 months (**Figure S30a, b**, Supporting Information). At a low-temperature environment of -40 ℃, the molecular motion of erythritol in binary SA is significantly reduced, resulting in a marked suppression of both its nucleation and crystallization processes.^[5, 12, 13]^ To investigate the influence of temperature on molecular motion at the macroscopic level, the binary SA maintained at -40 ℃ and 24 ℃ are respectively encapsulated in two glass bottles. Subsequently, the glass bottles are inverted to assess the fluidity of the binary SA. The viscosity of binary SA at -40 ℃ is considerably greater than that observed at 24 ℃. Specifically, the binary SA maintained at -40 ℃ remains at the top of the glass bottle, whereas that maintained at 24 ℃ exhibits a flowing behavior along the inner wall until it gradually accumulates at the bottom of the glass bottle (**Figure S30c, d**, Supporting Information).


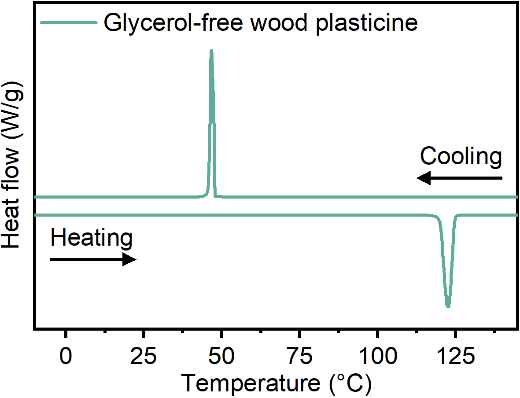


**Figure S31.** DSC curve of glycerol-free wood plasticine during the heating and cooling process, showing the spontaneous melting-crystallization phase change.

**
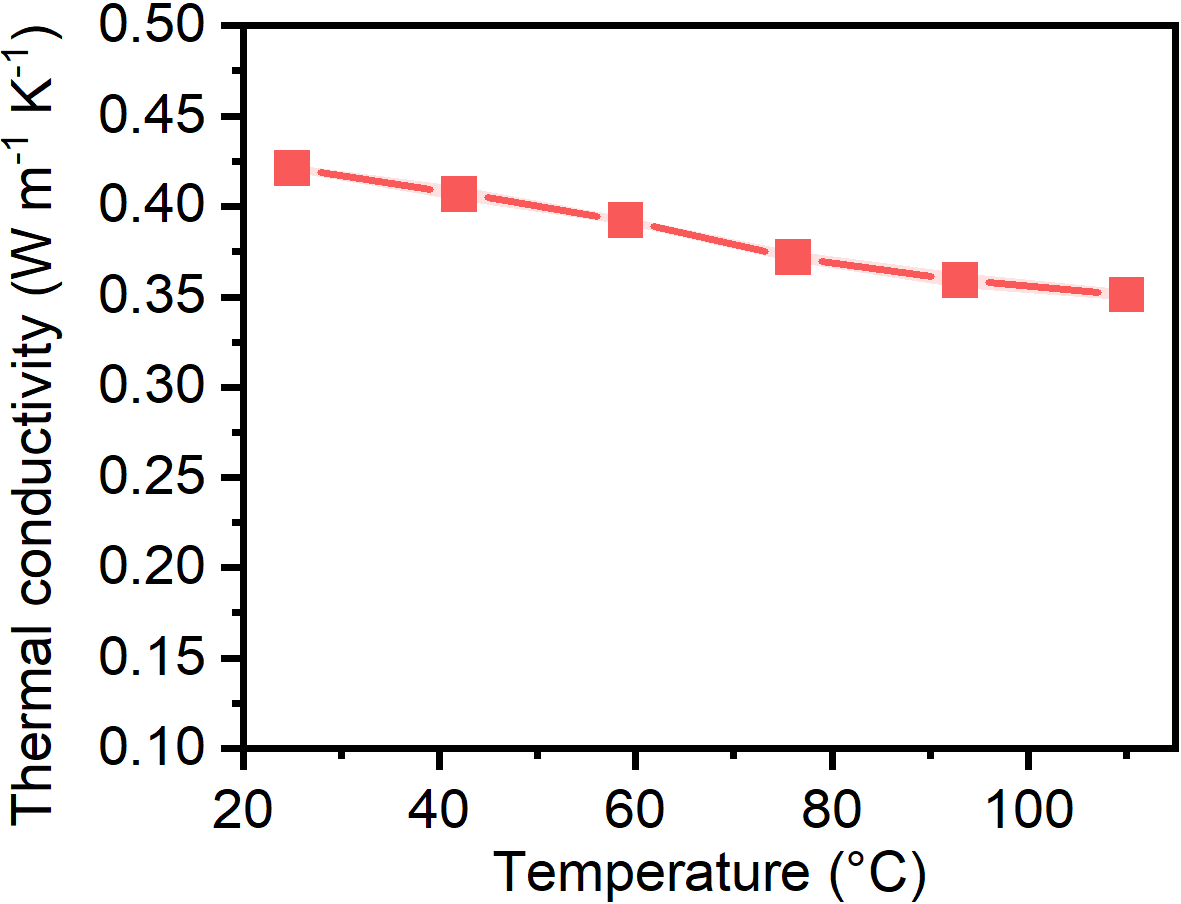
**

**Figure S32.** Thermal conductivity of wood plasticine at different temperatures.

**
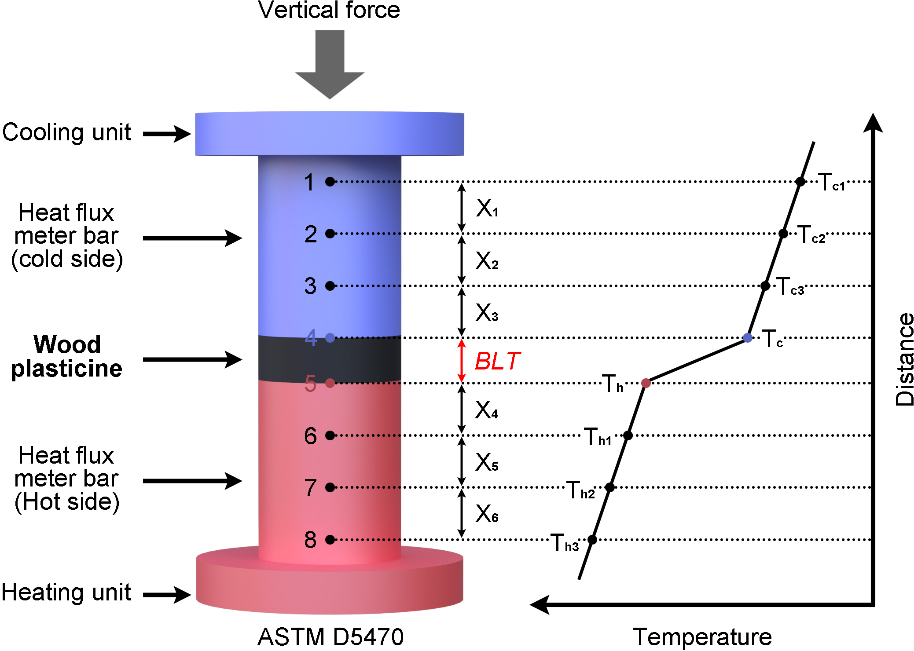
**

**Figure S33.** Schematic diagram illustrating the measurement principle of contact thermal resistance in accordance with the ASTM D5470 standard. The heat flux meter bar is constructed from copper.

The contact thermal resistance is a critical parameter for quantifying the impedance of heat transfer across the interface between two contacting surfaces. The contact thermal resistance is calculated using Equation (3):

*R*_contact_ **=** *R*_total_ - *R*_bulk_ (3)

where *R*_contact_ is the contact thermal resistance, *R*_total_ is the the total thermal resistance during the heat transfer process, and *R*_bulk_ is the bulk thermal resistance of thermal interface material (TIM). The *R*_total_ was quantified in accordance with the ASTM D5470 standard during the heat transfer process (**Figure S33**, Supporting Information). The points 1-8 represent the locations of the temperature measurement points along the heat flux meter bar. For this measuring equipment, the dimensions of X_1_ through X_6_ are uniformly set to 10 mm. T_c1_, T_c2_, and T_c3_ represent the temperatures at measurement points 1, 2, and 3, respectively, while T_h1_, T_h2_, and T_h3_ correspond to the temperatures at measurement points 6, 7, and 8, respectively. T_c_ and T_h_ represent the upper surface temperature and lower surface temperature of the tested wood plasticine, respectively. Q_c_ and Q_h_ represent the heat fluxes through the cold meter bar and hot meter bar during the testing process, respectively. Q_c_ and Q_h_ are calculated using Equations (4) and (5), respectively:

$\text{Q}_{\text{c}}\text{ }\text{=}{\text{ }\text{k}}_{\text{cu}}\text{ }\text{×}\text{ }\text{A}_{\text{cu}}\text{ }\text{×}\text{ }\frac{\text{T}_{\text{c3}}\text{ - T}_{\text{c1}}}{\text{X}_{\text{1}}\text{ }\text{+}\text{ }\text{X}_{\text{2}}}$ (4)

$\text{Q}_{\text{h}}\text{ }\text{=}{\text{ }\text{k}}_{\text{cu}}\text{ }\text{×}\text{ }\text{A}_{\text{cu}}\text{ }\text{×}\text{ }\frac{\text{T}_{\text{h3}}{\text{ -}\text{ }\text{T}}_{\text{h1}}}{\text{X}_{\text{5}}\text{ }\text{+}\text{ }\text{X}_{\text{6}}}$ (5)

where *k*_Cu_ and *A*_Cu_ represent the thermal conductivity and heat transfer area of the heat flux meter bar, respectively. The average heat flux can be determined utilizing Equation (6):

$\text{Q}\text{ }\text{=}\text{ }\frac{\text{Q}_{\text{c}}\text{ }\text{+}\text{ }\text{Q}_{\text{h}}}{\text{2}}$ (6)

When the measuring equipment attains a steady state, the relationship between the temperature at the test points and the distance between these points can be described by Equations (7) and (8):

$\text{k}_{\text{cu}}\text{ }\text{×}\text{ }\text{A}_{\text{cu}}\text{ }\text{×}\text{ }\frac{\text{T}_{\text{c3}}{\text{ }\text{-}\text{ }\text{T}}_{\text{c1}}}{\text{X}_{\text{1}}\text{ }\text{+}\text{ }\text{X}_{\text{2}}}\text{ }\text{=}{\text{ }\text{k}}_{\text{cu}}\text{ }\text{×}\text{ }\text{A}_{\text{cu}}\text{ }\text{×}\text{ }\frac{\text{T}_{\text{c}}{\text{ }\text{-}\text{ }\text{T}}_{\text{c3}}}{\text{X}_{\text{3}}}$ (7)

$\text{k}_{\text{cu}}\text{ }\text{×}\text{ }\text{A}_{\text{cu}}\text{ }\text{×}\text{ }\frac{\text{T}_{\text{h3}}{\text{ }\text{-}\text{ }\text{T}}_{\text{h1}}}{\text{X}_{\text{5}}\text{ }\text{+}\text{ }\text{X}_{\text{6}}} \text{=} \text{k}_{\text{cu}}\text{ }\text{×}\text{ }\text{A}_{\text{cu}}\text{ }\text{×}\text{ }\frac{\text{T}_{\text{h1}}{\text{ }\text{-}\text{ }\text{T}}_{\text{h}}}{\text{X}_{\text{4}}}$ (8)

The surface temperatures of the upper (T_c_) and lower (T_h_) surfaces of the tested wood plasticine are calculated using Equations (9) and (10).

$\text{T}_{\text{c}}\text{ }\text{=}\text{ }\text{T}_{\text{c3}}\text{ }\text{+}\text{ }\frac{\text{(}\text{T}_{\text{c3}} \text{-} \text{T}_{\text{c1}}\text{)}{\text{ }\text{×}\text{ }\text{X}}_{\text{3}}}{\text{X}_{\text{1}}\text{ }\text{+}\text{ }\text{X}_{\text{2}}}$ (9)

$\text{T}_{\text{h}}\text{ }\text{=}\text{ }\text{T}_{\text{h1}}\text{ }\text{-}\text{ }\frac{\text{(}\text{T}_{\text{h3}} \text{-} \text{T}_{\text{h1}}\text{)}{\text{ }\text{×}\text{ }\text{X}}_{\text{4}}}{\text{X}_{\text{5}}\text{ }\text{+}\text{ }\text{X}_{\text{6}}}$ (10)

The *R*_total_ and *R*_bulk_ can subsequently be determined using Equations (11) and (12):

$\text{R}_{\text{total}}\text{ }\text{=}\text{ }\text{A}_{\text{TIM}}\text{ }\text{×}\text{ }\frac{\text{T}_{\text{h}}\text{ }\text{-}\text{ }\text{T}_{\text{c}}}{\text{Q}}$ (11)

$\text{R}_{\text{bulk}}\text{ }\text{=}\text{ }\frac{\text{BLT}\text{ }}{\text{k}_{\text{TIM}}}$ (12)

where the *A*_TIM_ and *k*_TIM_ represent the area and thermal conductivity of the tested wood plasticine, respectively, while the bond line thickness (BLT) denotes the thickness of the tested wood plasticine under applied pressure. Consequently, the *R*_contact_ can also be expressed using Equation (13):

$\text{R}_{\text{contact}}\text{ }\text{=}\text{ }\text{A}_{\text{TIM}}\text{ }\text{×}\text{ }\frac{\text{T}_{\text{h}}\text{ }\text{-}\text{ }\text{T}_{\text{c}}}{\text{Q}}\text{ - }\frac{\text{BLT}\text{ }}{\text{k}_{\text{TIM}}}$ (13)


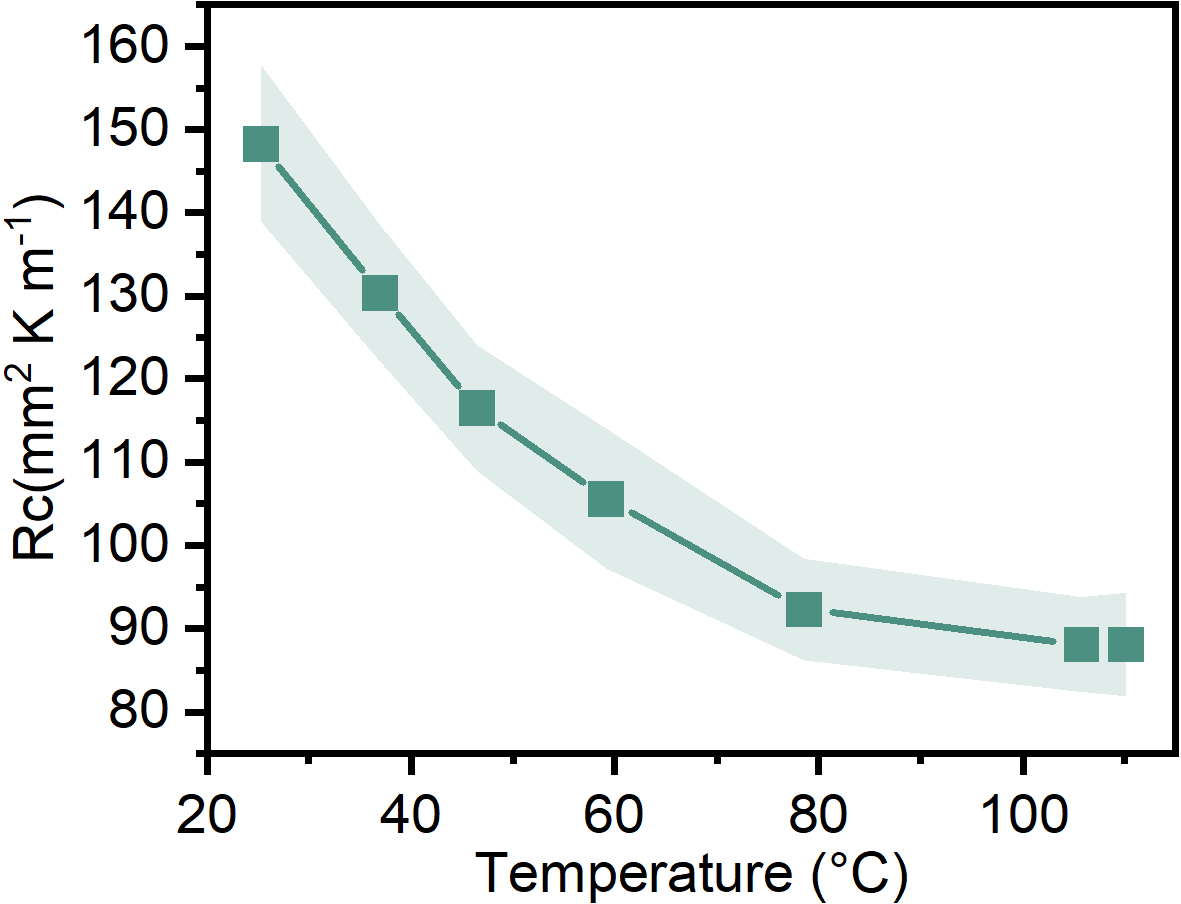


**Figure S34.** Contact thermal resistance (R_c_) of wood plasticine at different temperatures.

The contact thermal resistance of wood plasticine at 105 ℃ is nearly identical to that at 110 ℃, as this material has already undergone a complete phase change from its crystallized state to its melted state by 105 ℃.


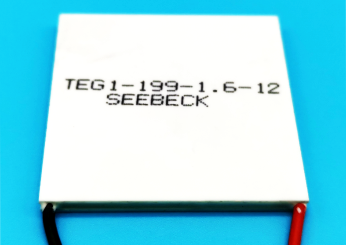


**Figure S35.** Photograph of commercial thermoelectric generator (TEG) (size: 4 cm × 4 cm × 0.3 cm).


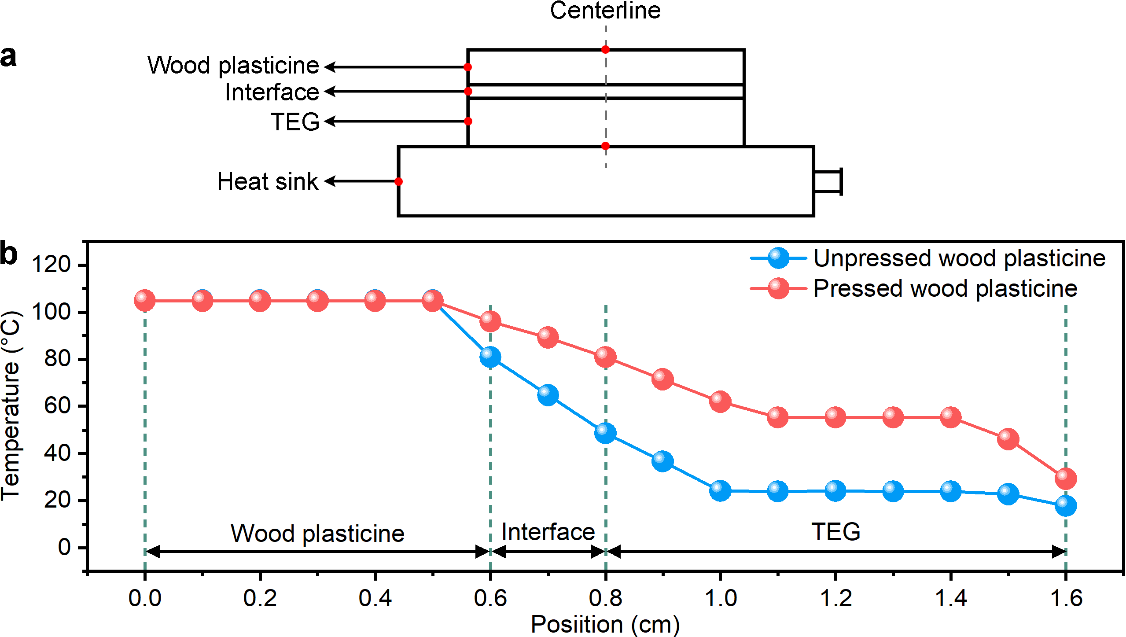


**Figure S36.** a) Schematic diagram of solar-thermal-electric conversion apparatus. b) Temperature distribution along the centerline from wood plasticine to TEG.


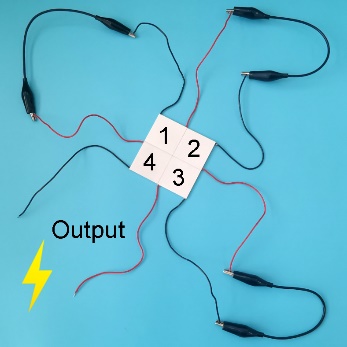


**Figure S37.** Photograph of a large-scale TEG consisting of four smaller TEGs connected in series (size: 4 cm × 4 cm × 0.3 cm).


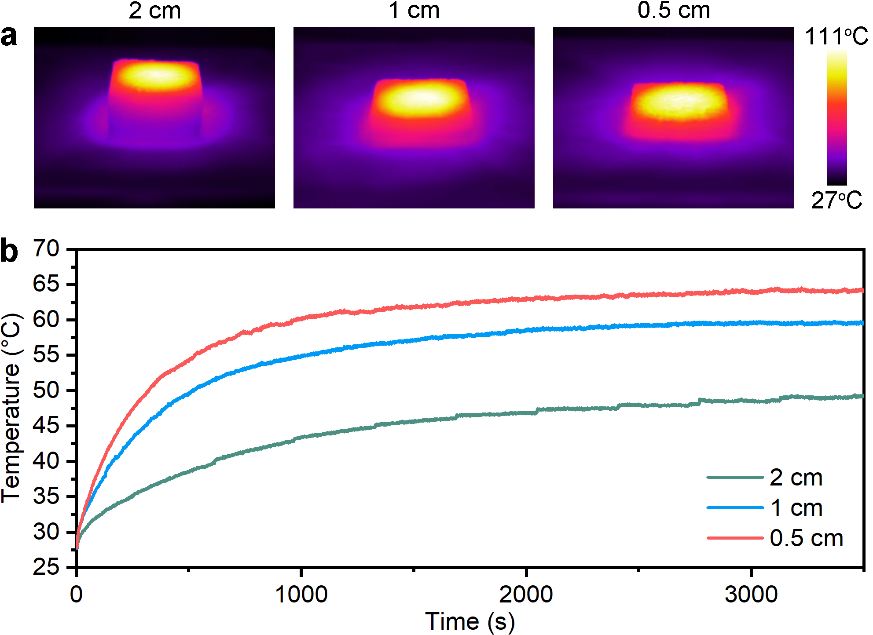


**Figure S38.** a) Infrared thermal images and b) time-temperature curves of wood plasticine with thicknesses ranging from 2 cm to 0.5 cm under focused ambient sunlight.


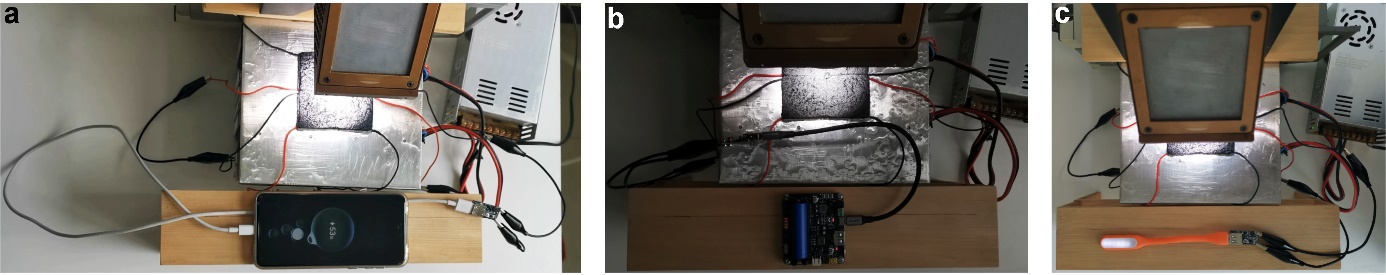


**Figure S39.** Photographs of solar-thermal-electric conversion apparatus for charging a) a smartphone, b) a 14500 lithium-ion battery, and c) an LED.

In consideration of the intermittency and fluctuation of sunlight, the xenon lamp source is utilized to replace concentrated sunlight, which provides a better representation of the solar-thermal-electric conversion process. The simulated solar intensity is set at 600 mW cm^-2^.


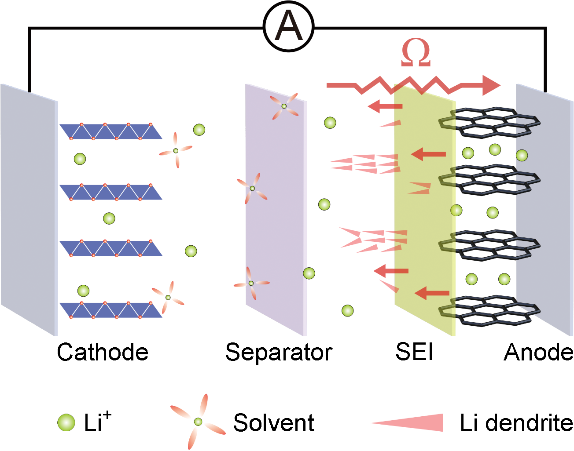


**Figure S40.** Schematic diagram of lithium-ion battery performance degradation under low-temperature environments.

Under low-temperature conditions, lithium-ion batteries usually experience capacity degradation, slower charging rates, and an elevated risk of internal short circuits. These issues stem from the increased viscosity of the electrolyte, reduced ion conduction rates within the electrolyte, non-uniform internal electrochemical reactions, and the formation of lithium dendrites.


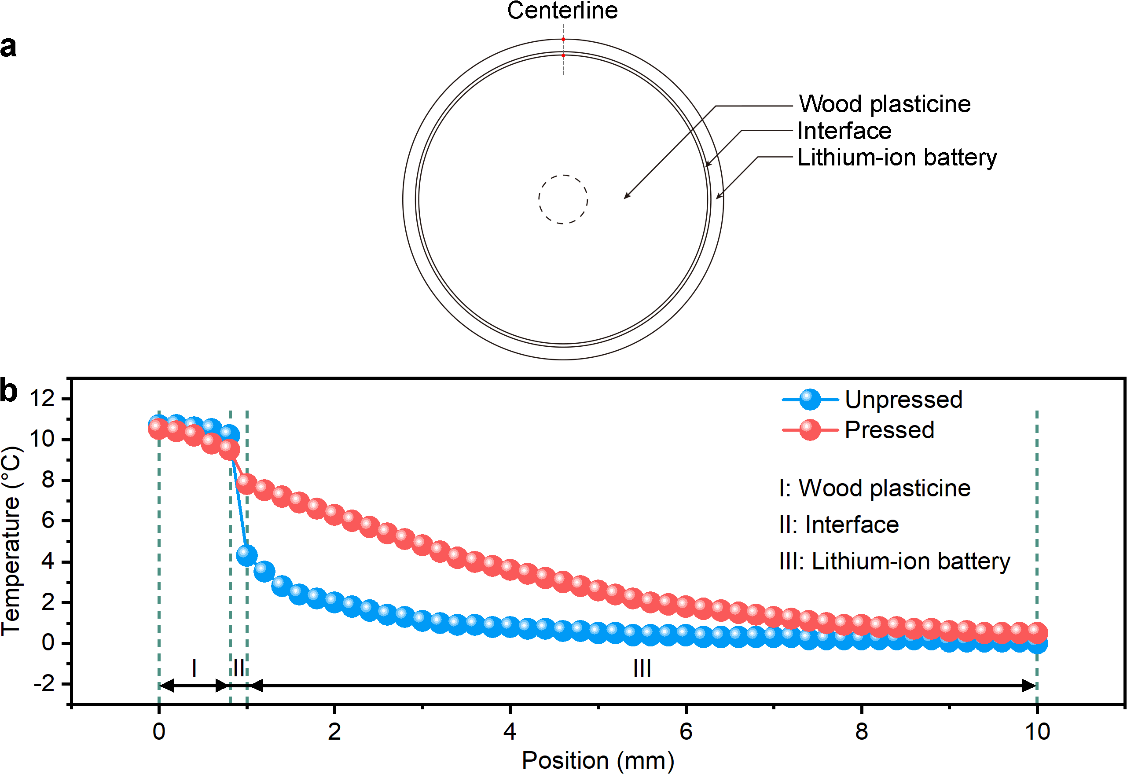


**Figure S41.** a) Schematic diagram of wood plasticine-wrapped lithium-ion battery. b) Temperature distribution along the centerline from the wood plasticine to the lithium-ion battery.


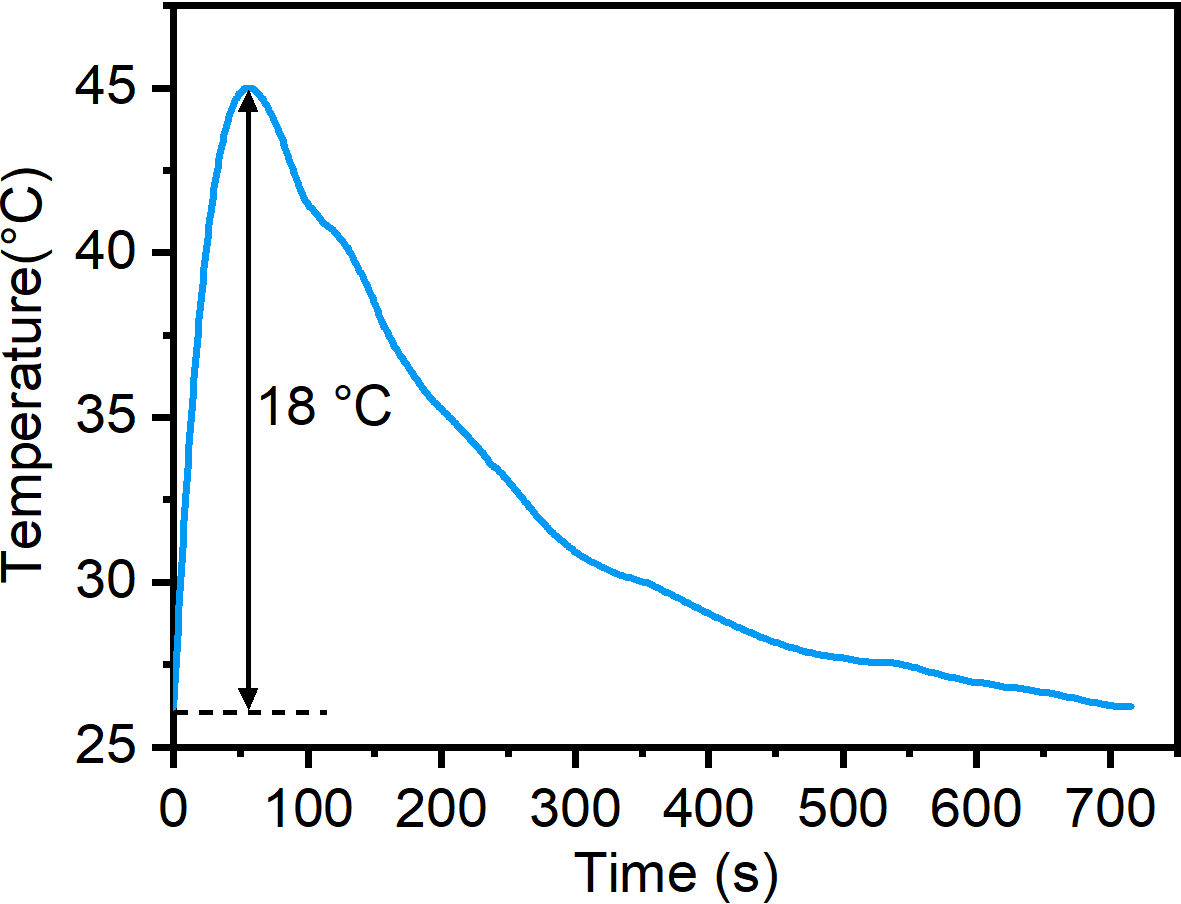


**Figure S42.** Time-temperature curve of wood plasticine during its heat supply to the human arm.


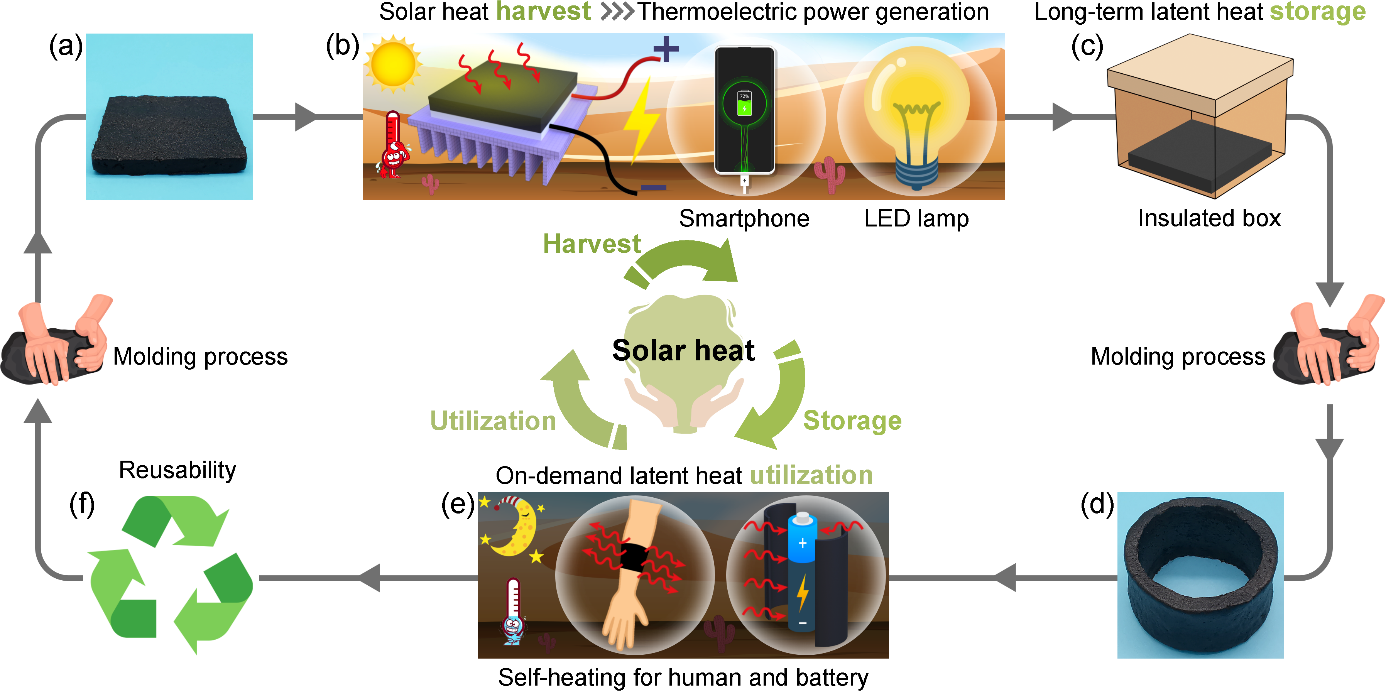


**Figure S43.** Schematic overview of wood plasticine for cyclic solar heat harvesting, storage, and utilization. a) Photograph of a cuboid wood plasticine. b) Schematic illustration of solar heat harvesting for thermoelectric conversion. c) Schematic illustration depicting long-term latent heat storage using wood plasticine. d) Photograph of a tube-shaped wood plasticine after the molding process. e) Schematic illustration showing the application of wood plasticine in battery and personal thermal management. f) Schematic illustration demonstrating the reusability of wood plasticine.


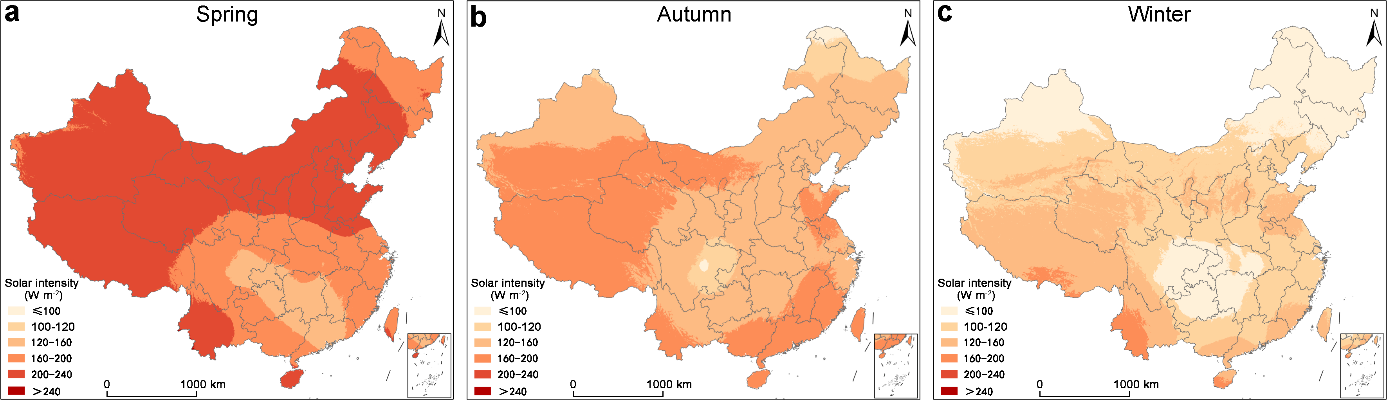


**Figure S44.** Solar intensity distribution across different regions of China during a) spring, b) autumn, and c) winter.

**Figure 6k** and **Figure S44** (Supporting Information) present a detailed analysis of the seasonal variations in solar radiation intensity distribution across various regions of China. The northwestern regions (including Xinjiang, Qinghai, Gansu, and Shaanxi), the northern regions (including Inner Mongolia, Shanxi, and Hebei) and Shandong province exhibit extremely high solar radiation intensity, exceeding 240 W m^-2^ during summer. This indicates a significant abundance of solar energy resources in these areas.

**
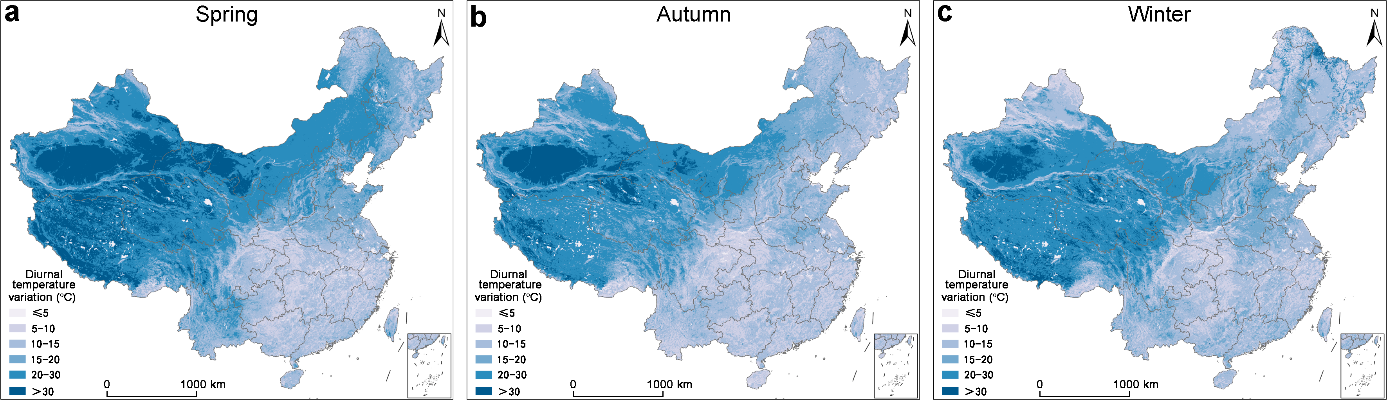
**

**Figure S45.** Diurnal temperature variation distribution across different regions of China during a) spring, b) autumn, and c) winter.

**Figure 6l** and **Figure S45** (Supporting Information) present a detailed analysis of the seasonal variations in diurnal temperature variations distribution across various regions of China. The annual diurnal temperature variation exceeds 30 ℃ in the Tibet, Xinjiang, and Qinghai regions.

**Table S1.** The influence of glycerol content on the thermal properties and crystal size of erythritol.

|  | *T*_m_ (℃) | Δ*H*_m_ (J g^-1^) | Crystal size (μm) |
| --- | --- | --- | --- |
| Erythritol (1:0) | 120.3 ± 1.5 | 322.7 ± 1.7 | 74.7 ± 9.8 |
| Binary SA (1:0.5) | 82.5 ± 1.3 | 170.1 ± 1.1 | 33.7 ± 3.8 |
| Binary SA (1:1) | 75.0 ± 1.1 | 126.5 ± 0.5 | 16.6 ± 0.4 |
| Binary SA (1:2) | 65.5 ± 0.8 | 68.1 ± 1.3 | 8.1 ± 0.9 |

**Table S2.** Thermal conductivity of wood plasticine at different temperatures.

| Temperature (℃) | Thermal conductivity (W m^-1^ K^-1^) |
| --- | --- |
| 25 | 0.421 ± 0.002 |
| 42 | 0.407 ± 0.004 |
| 59 | 0.392 ± 0.002 |
| 76 | 0.372 ± 0.003 |
| 93 | 0.359 ± 0.004 |
| 110 | 0.351 ± 0.003 |

**Table S3.** Contact thermal resistance of wood plasticine at different pressures.

| Pressure (psi) | R_c1_ (mm^2^ K W^-1^) | R_c2_ (mm^2^ K W^-1^) |
| --- | --- | --- |
| 10 | 203.1 ± 14.1 | 123.6±6.1 |
| 20 | 178.5 ± 11.5 | 106.3±4.5 |
| 30 | 160.7 ± 8.9 | 94.5±5.7 |
| 40 | 148.3 ± 9.4 | 88.1±6.1 |

R_c1_: contact thermal resistance before phase change; R_c2_: contact thermal resistance after phase change.

**Table S4.** Contact thermal resistance of wood plasticine at different temperatures.

| Temperature (℃) | R_c_ (mm^2^ K W^-1^) |
| --- | --- |
| 25.3 | 148.3 ± 9.4 |
| 36.9 | 130.4 ± 8.2 |
| 46.5 | 116.5 ± 7.6 |
| 59.2 | 105.6 ± 8.4 |
| 78.5 | 92.3 ± 6.1 |
| 105.8 | 88.1 ± 5.7 |
| 110.1 | 88.1 ± 6.2 |

**Table S5.** Contact thermal resistance of non-phase-change TIMs from previously reported literature.

|  | R_c_ (mm^2^ K W^-1^) | References |
| --- | --- | --- |
| Aluminum solid | 700 | ^[14]^ |
| Thermal pad | 300 | ^[14]^ |
| LM/BN/PDMS composite | 253 | ^[15]^ |
| Aligned CNT composite film | 200 | ^[16]^ |
| Commercial silicon sheet | 160 | ^[17]^ |

Liquid metal (LM), boron nitride (BN), polydimethylsiloxane (PDMS), and carbon nanotube (CNT).

**Table S6.** Contact thermal resistance of phase-change TIMs from previously reported literature.

|  | R_c1_ (mm^2^ K W^-1^) | R_c2_ (mm^2^ K W^-1^) | References |
| --- | --- | --- | --- |
| PEG/polyester resin/graphene | 2700 | 90 | ^[17]^ |
| Al_2_O_3_/olefin block copolymer/PA | 2614.7 | 197.3 | ^[18]^ |
| Graphene composite hydrogel | 1700.6 | 48.7 | ^[19]^ |
| SBS/EVA/PA/EG | 442 | 218 | ^[20]^ |
| PA/polyolefin elastomer | - | 291 | ^[21]^ |
| SA/polyolefin elastomer | - | 239 | ^[22]^ |
| Wood plasticine | 148.3 ± 9.4 | 88.1 ± 6.1 | This work |

R_c1_: contact thermal resistance before phase change; R_c2_: contact thermal resistance after phase change. Polyethylene glycol (PEG), paraffin (PA), styrene−butadiene−styrene polymer (SBS), ethylene vinyl acetate (EVA), expanded graphite (EG), and stearic acid (SA).

**Table S7.** Common composite PCMs mentioned in previous studies.

| Materials | References |
| --- | --- |
| Cellulose aerogel PCMs | ^[23-25]^ |
| Polyurethane-based PCMs | ^[26-28]^ |
| Salt gel PCMs | ^[29-31]^ |
| PDMS-based PCMs | ^[32-34]^ |

**Table S8.** The parameters of wood plasticine and heat sink used in ANSYS Icepak simulation.

|  | Size (cm^3^) | Materials | *k* (W m^-1^ K^-1^) |
| --- | --- | --- | --- |
| Wood plasticine | 4 × 4 × 0.8 | - | 0.421 |
| Heat sink | 6 × 6 × 1 | Copper | 401 |

To simulate the photothermal conversion in a real-world environment, the wood plasticine is modeled as a heat source in ANSYS Icepak with a power output of 30 W. The room temperature is maintained at 25 ℃, and the atmosphere is set to 1 atm. The temperature of cooling liquid at the inlet is -5 ℃ with a volume flow rate of 200 mL min^-1^.

**Table S9.** The charge-discharge rate of lithium-ion batteries and the corresponding charge-discharge time.

|  | Mode | Rate (C) | Time (min) |
| --- | --- | --- | --- |
| I | Charging | 1 | 15 |
| II | Discharging | 1 | 5 |
| III | Charging | 2 | 15 |
| IV | Discharging | 2 | 5 |
| V | Charging | 3 | 10 |
| VI | Discharging | 2 | 10 |
| VII | Charging | 4 | 10 |
| VIII | Discharging | 2 | 10 |

**Table S10.** The parameters of wood plasticine and lithium-ion battery used in ANSYS Icepak simulation.

|  | Height | Radius | Materials | *k* (W m^-1^ K^-1^) |
| --- | --- | --- | --- | --- |
| Wood plasticine | 6.5 | 0.9^a^, 1^b^ |  | 0.421 |
| Battery | 6.5 | 0.9 |  | 1 |

The exothermic process of wood plasticine under external force is modeled as a heat source with an output power of 10 W in ANSYS Icepak simulations. The ambient temperature is maintained at 0 ℃, while the atmospheric pressure is set to 1 atm. The superscripts “a” and “b” in the table denote the inner diameter and outer diameter, respectively.

**References：**

[1] https://worldclim.org/.

[2] https://cstr.cn/18406.11.Meteoro.tpdc.271252.

[3] C. Montanari, Y. Li, H. Chen, M. Yan, L. A. Berglund, *ACS Appl. Mater. Interfaces* **2019**, *11*, 20465.

[4] J. Chen, Y. Kou, S. Zhang, X. Zhang, H. Liu, H. Yan, Q. Shi, *Angew. Chem.-Int. Edit.* **2024**, *63*, e202400759.

[5] S. Yang, H.-Y. Shi, J. Liu, Y.-Y. Lai, Ö. Bayer, L.-W. Fan, *Nat. Commun.* **2024**, *15*, 4948.

[6] C. Chen, Y. Kuang, S. Zhu, I. Burgert, T. Keplinger, A. Gong, T. Li, L. Berglund, S. J. Eichhorn, L. Hu, *Nat. Rev. Mater.* **2020**, *5*, 642.

[7] J. Li, C. Chen, J. Y. Zhu, A. J. Ragauskas, L. Hu, *Accounts Mater. Res.* **2021**, *2*, 606.

[8] C. Chen, J. Song, S. Zhu, Y. Li, Y. Kuang, J. Wan, D. Kirsch, L. Xu, Y. Wang, T. Gao, Y. Wang, H. Huang, W. Gan, A. Gong, T. Li, J. Xie, L. Hu, *Chem* **2018**, *4*, 387.

[9] H. Guan, Z. Cheng, X. Wang, *ACS Nano* **2018**, *12*, 10365.

[10] J. Song, C. Chen, Z. Yang, Y. Kuang, T. Li, Y. Li, H. Huang, I. Kierzewski, B. Liu, S. He, T. Gao, S. U. Yuruker, A. Gong, B. Yang, L. Hu, *ACS Nano* **2018**, *12*, 140.

[11] B. Guo, Y. Liang, R. Dong, *Nat. Protoc.* **2023**, *18*, 3322.

[12] K. Turunen, M. R. Yazdani, S. Puupponen, A. Santasalo-Aarnio, A. Seppälä, *Appl. Energy* **2020**, *266*, 114890.

[13] S. Puupponen, A. Seppälä, *Sol. Energy Mater. Sol. Cells* **2018**, *180*, 59.

[14] J.-W. Zhao, R. Zhao, Y.-K. Huo, W.-L. Cheng, *Int. J. Heat Mass Transf.* **2019**, *140*, 705.

[15] Z. Wang, J. Li, N. Ye, H. Zhang, D. Yang, Y. Lu, *Compos. Sci. Technol.* **2023**, *233*, 109903.

[16] H. Huang, C. Liu, Y. Wu, S. Fan, *Adv. Mater.* **2005**, *17*, 1652.

[17] C. Liu, W. Yu, J. Yang, Y. Zhang, H. Xie, *Int. Commun. Heat Mass Transf.* **2021**, *127*, 105553.

[18] C. Liu, C. Chen, W. Yu, M. Chen, D. Zhou, H. Xie, *Int. J. Therm. Sci.* **2020**, *152*, 106293.

[19] J. Yang, W. Yu, C. Liu, H. Xie, H. Xu, *Compos. Sci. Technol.* **2022**, *219*, 109223.

[20] J. Deng, X. Li, R. Liang, C. Li, G. Zhang, D. Zhou, Q. Deng, Z. Wu, *ACS Appl. Energ. Mater.* **2023**, *6*, 1810.

[21] L.-M. Peng, Z. Xu, J. Yang, L. Bai, R.-Y. Bao, M.-B. Yang, W. Yang, *Chem. Eng. J.* **2023**, *455*, 140891.

[22] H. Huang, C. Wang, T. Wu, Z. Wu, J. Zheng, *Surf. Interfaces* **2024**, *55*, 105406.

[23] K. Liu, M. Sun, R. Guo, H. Wang, T. Chen, Y. Li, C. Wang, H. Yang, *Carbohydr. Polym.* **2024**, *344*, 122532.

[24] M. Song, J. Jiang, J. Zhu, Y. Zheng, Z. Yu, X. Ren, F. Jiang, *Carbohydr. Polym.* **2021**, *272*, 118460.

[25] X. Wei, F. Xue, X.-d. Qi, J.-h. Yang, Z.-w. Zhou, Y.-p. Yuan, Y. Wang, *Appl. Energy* **2019**, *236*, 70.

[26] Y. Zhang, P. Wu, Y. Meng, R. Lu, S. Zhang, B. Tang, *Chem. Eng. J.* **2023**, *464*, 142650.

[27] Y. Kou, K. Sun, J. Luo, F. Zhou, H. Huang, Z.-S. Wu, Q. Shi, *Energy Storage Mater.* **2021**, *34*, 508.

[28] X. Geng, M. Qin, Z. Shen, F. Xiong, J. Di, C. Yang, Y. Wang, S. Gao, S. Gao, Q. Wang, R. Zou, *Adv. Func. Mater.* **2024**, *35*, 2418848.

[29] Y. Liu, J. Zhou, Y. Li, Z. Wang, X. Sun, H. Yang, C. Wang, *Small* **2023**, *19*, 2305134.

[30] Y. Liu, J. Zhou, Y. Li, X. Sun, Z. Wang, H. Yang, C. Wang, *Adv. Func. Mater.* **2024**, *34*, 2400203.

[31] Y. Fang, X. Xiong, L. Yang, W. Yang, H. Wang, Q. Wu, Q. Liu, J. Cui, *Adv. Func. Mater.* **2023**, *33*, 2301505.

[32] Z.-j. Huang, R.-q. Wang, W.-j. Jiang, Y.-l. Liu, T.-y. Zhu, D.-x. Sun, J.-h. Yang, X.-d. Qi, Y. Wang, *Compos. Sci. Technol.* **2024**, *255*, 110736.

[33] S. Ki, S. Shin, S. Cho, S. Bang, D. Choi, Y. Nam, *Adv. Sci.* **2024**, *11*, 2310185.

[34] W.-j. Jiang, R.-q. Wang, T.-y. Zhu, M. Feng, D.-x. Sun, J.-h. Yang, X.-d. Qi, Y. Wang, *Chem. Eng. J.* **2024**, *479*, 147622.
